# Tanshinone IIA inhibits heat-induced growth of p53-mutant Huh-7 hepatocellular carcinoma by modulating osmotic homeostasis and glycolysis through targeting ALDH7A1

**DOI:** 10.1038/s41420-025-02795-0

**Published:** 2025-10-31

**Authors:** Hao Li, Shuguang Ju, Jiacheng Wang, Donglin Kuang, Pengfei Chen, Mengfan Zhang, Ruijie Qian, Chao Liang, Daqian Han, Xuhua Duan

**Affiliations:** Department of Interventional Radiology, The First Affiliated Hospital of Zhengzhou University, Zhengzhou, Henan Province China

**Keywords:** Glycobiology, Hepatocellular carcinoma

## Abstract

Thermal ablation offers minimally invasive treatment options for hepatocellular carcinoma (HCC) therapy. However, local recurrence due to sublethal temperatures enhances tumor cell survival. This study aims to investigate the tumor-promoting effects of hyperthermia on HCC cells, the role of tanshinone IIA (Tan IIA) in mitigating these effects, and the underlying mechanisms involved. We observed that temperature at 44 °C increased the aggressiveness of HCC cells, and Tan IIA inhibited cell viability and cell invasion, and induced cell cycle arrest and apoptosis of heat-pretreated HCC cells. ALDH7A1 was identified as a target of Tan IIA, and its altered expression resulted in dysregulation of cell viability, invasion, apoptosis, ATP production, glycolysis, osmolyte levels, and reactive oxygen species (ROS). Under hyperosmotic conditions, ALDH7A1 knockdown sensitized heated Huh-7 cells, while its overexpression promoted cell survival and invasion, with corresponding changes in energy metabolism and enzymatic products. Tan IIA and the specific ALDH7A1 inhibitor, 4-diethylaminobenzaldehyde, demonstrated similar effects on gene expression patterns, glycolysis, osmotic regulation, and ROS levels in heated Huh-7 cells. Moreover, Tan IIA is able to direct interact with ALDH7A1 protein. In vitro, Tan IIA combined with hyperosmotic stress significantly inhibited cell invasion and induced apoptosis in heat-induced Huh-7 cells and ALDH7A1 overexpression partially reversed the effects of Tan IIA. In vivo, Tan IIA combined with hyperosmotic stress or glycolysis inhibitor yielded better therapeutic efficacy for HCC. In conclusion, Tan IIA sensitizes HCC cells to sublethal heat by targeting ALDH7A1, leading to disrupted glycolytic and osmolytic balance, subsequently hindering tumor cell survival and increasing apoptosis. These findings highlight a potentially novel strategy for preventing or treating recurrent HCC post-thermal ablation using Tan IIA with hyperosmotic reagents.

## Introduction

Hepatocellular carcinoma (HCC) remains a major global health challenge, ranking as the sixth most common malignancy and the fourth leading cause of cancer-related deaths worldwide [1]. The aggressive nature of HCC, coupled with its late diagnosis and resistance to conventional therapies, underscores the urgent need for innovative treatment strategies [1]. Thermal ablation therapy using radiofrequency, microwave, or laser, defined as the exposure of tumor tissues to extremely elevated temperatures (50–100 °C), has garnered attention as one of the best strategies with minimal invasion to treat focal malignancy [2]. The biological rationale for thermal ablation lies in its ability to disrupt protein structures, enhance drug uptake, and induce apoptosis in cancer cells. However, thermal ablation faces setbacks in clinical practice. The local recurrence rate of HCC treated with radiofrequency ablation (RFA) is high, which varies from 18.2% to 46.6%, and the median time to recurrence was 13 months (IQR 6–23 months) [3, 4]. Furthermore, some preclinical studies have demonstrated that certain sublethal temperature ranges potentially promote HCC cell survival and proliferation, as well as metastasis and stemness of HCC cells [5, 6].

Tanshinone IIA (Tan IIA), a lipophilic diterpenoid derived from *Salvia miltiorrhiza*, has been studied for its anti-cancer properties [7]. Tan IIA exerts its anti-tumor effects through a variety of mechanisms, including the induction of apoptosis via p53/PTPN11/SHP2 pathway, and induction of cell cycle arrest via downregulation of cyclin D1, A, and E, and suppression of metastasis via inhibition of STAT3 activation [7, 8]. As_2_O_3_ (arsenic trioxide) is a famous chemotherapeutic drug especially for acute promyelocytic leukemia, and it can also inhibit tumor cell growth through multiple mechanisms, including destroying the cells, preventing their division, or preventing their spread [9]. 3-bromopyruvate (3-BrPA), a potent glycolytic inhibitor, has demonstrated significant potential as a promising anticancer drug via perturbation of various signaling and metabolic factors [10]. The ability of these drugs to target numerous signaling pathways makes them promising candidates for combination therapies.

Osmoregulation is required for cell survival of all cancers under comprehensive solid stress caused by tumor cell accumulation [11] or under hyperosmotic stress due to high glucose etc. in the tissue [12]. In normal or tumor cells, mitochondria directly senses the change of osmosis to induce Warburg-like metabolic remodeling by regulating pyruvate dehydrogenase phosphorylation [13]. Recently, a novel cancer therapy called “Targeted Osmotic Lysis” has gained the researchers’ attention for its reported potential clinical benefits in advanced carcinomas, including providing a cure or improved survival for some cases [14]. Meanwhile, some preclinical studies have pointed out that hyperosmotic stress reprograms tumor cells and perspectively makes them vulnerable to anticancer drugs [15, 16]. Therefore, we propose that increased osmosis is likely to inhibit tumor growth or sensitize the survived HCC cells to anti-tumor drugs after thermal ablation.

This study aims to identify a potential drug that can prevent or conquer the recurrence of HCC following thermal ablation, and then to elucidate the molecular mechanisms underlying this drug’s action, trying to propose more potential therapeutic strategy for recurrent HCC following thermal ablation. By integrating in vitro and in vivo models, our research may provide a comprehensive understanding of the potential therapeutic benefits of combining anti-tumor drug with hyperthermia in the treatment of HCC.

## Results

### Moderate high temperature enhanced viability of HCC cells, and Tan IIA showed stable anti-tumor effects on HCC cells with or without heat pretreatment

**Fig. 1:**
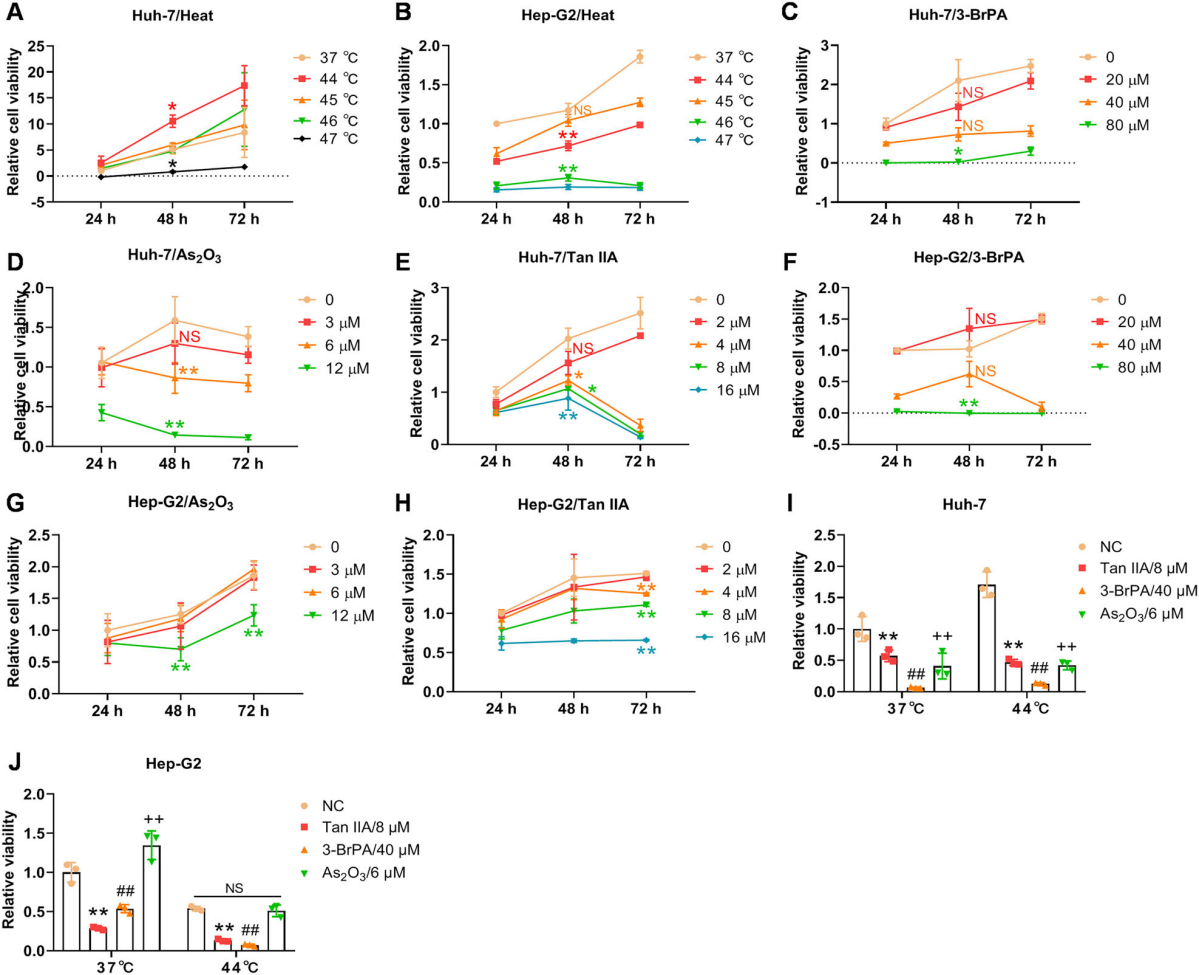
The effects of 3 antitumor drugs on hepatocellular carcinoma (HCC) cell lines pretreated with or without heat. **A**, **B** The viability of HCC cell lines Huh-7 and Hep-G2, pretreated with heat at various temperatures—37 °C, 44 °C, 45 °C, 46 °C, and 47 °C, respectively—was assessed after being reinoculated and cultured in new culture media at 37 °C. **C–H** The viability of Huh-7 and Hep-G2 cells following treatment with various concentrations of 3-BrPA (3-bromopyruvate), As_2_O_3_, or Tanshinone IIA (Tan IIA) as indicated. **I**, **J** The inhibitory effects of different antitumor drugs on HCC cells pretreated at 44 °C. For (**A**–**H**), *p < 0.05, **p < 0.01, compared with 37 °C at the same time point; the same color indicates the same group. For (**I**–**J**), **p < 0.01, Tan IIA/8 μM vs NC; ## *p* < 0.01, 3-BrPA/40 μM vs NC; ++ *p* < 0.01, As_2_O_3_/6 μM vs NC; all comparisons were performed on data at the same temperature. NS not significant.

In order to explore the temperatures that may stimulate the growth of HCC cells (Huh-7 and Hep-G2) and observe the impact of different temperatures on cell viability, we first treated cells at temperatures of 37, 45, 50, and 55 °C. The results showed that at temperatures of 50 and 55 °C, cell viability was greatly inhibited, while 45 °C had nearly no effect on cell viability compared to treatment at 37 °C (Fig. S1A, B). To sensitize the cells to temperature, we introduced three anti-cancer drugs (Tan IIA, 3-BrPA, and As_2_O_3_). The results showed that Tan IIA seemed to have a stronger inhibitory effect on both HCC cell lines treated at 45 °C compared to 37 °C; As_2_O_3_ at concentrations of 3 and 6 μM did not affect cell viability (thus a concentration of 12 μM was added later) (Fig. S1C, D). In addition, we observed that the cell viability of Hep-G2 cells pretreated at 45 °C was slightly higher than those pretreated at 37 °C (Fig. S1D). These results indicated that high temperature induction may not effectively kill HCC cells but even promotes their growth or proliferation, and Tan IIA can effectively inhibit the viability of HCC cells with or without heat pretreatment. Therefore, we further investigated the effects of more different high temperatures around 45 °C, including 44, 45, 46, and 47 °C. As a result, Huh-7 cells induced by 44 °C showed significantly enhanced viability, and 45 °C induced Hep-G2 cells had occasionally indistinct viability value at 48 h, although all the high temperatures were not able to increase the viability of Hep-G2 cells, compared with 37 °C (Fig. 1A, B).

To further confirm the anti-cancer effect of Tan IIA among these candidate drugs on HCC cells, we optimized drug concentrations and detected the effects of the three drugs on HCC cell viability under conditions with or without heat stimulation. The results showed that at a Tan IIA concentration of 4 μM, cell viability was not only inhibited at 48 h and 72 h but also showed a general reverse downward trend, especially in Huh-7 cells, while the other two drugs only inhibited the increase of cell viability as time went by (Fig. 1C-H). Therefore, the efficacy of Tan IIA may be better and more stable, and we further tested the effects of the three drugs on the viability of the HCC cells pretreated with a sublethal heat at 44 °C. As a result, Tan IIA and 3-BrPA seemed to have more stable inhibitory effects on cell viability with or without high temperature induction at 48 h, as shown in Fig. 1I-J, while As_2_O_3_ sometimes didn’t affect or even occasionally promoted the viability of Hep-G2 cells. Based on a comprehensive evaluation of factor,s including its anti-tumor effects, established research, and its connection to glycolysis and the interesting gene ALDH7A1, which is associated with hyperosmotic stress and a potential mechanism relevant to our clinical practice and other studies, we decided to focus on Tan IIA for further investigation.

According to our experience, the sensitivity of different cell lines or the same cell line in different states to temperatures within a specific range may vary significantly. Therefore, we briefly analyzed the transcription levels of proliferation markers PCNA and KI67 in Huh-7 cells after induction at different temperatures, including 37 °C, 42 °C, 43 °C, and 44 °C. The results showed that both 42 and 44 °C effectively stimulated the expression of cell proliferation markers PCNA and KI67 (Fig. S1E, F). The inhibitory effect of Tan IIA on proliferation in cells induced at 44 °C may be the most significant. Based on the above results, we chose to continue the following in-depth study using the temperature at 44 °C.

### The sublethal heat enhanced invasion and anti-apoptotic ability of HCC cells, and Tan IIA showed effective anti-tumor effects on Huh-7 cells

**Fig. 2:**
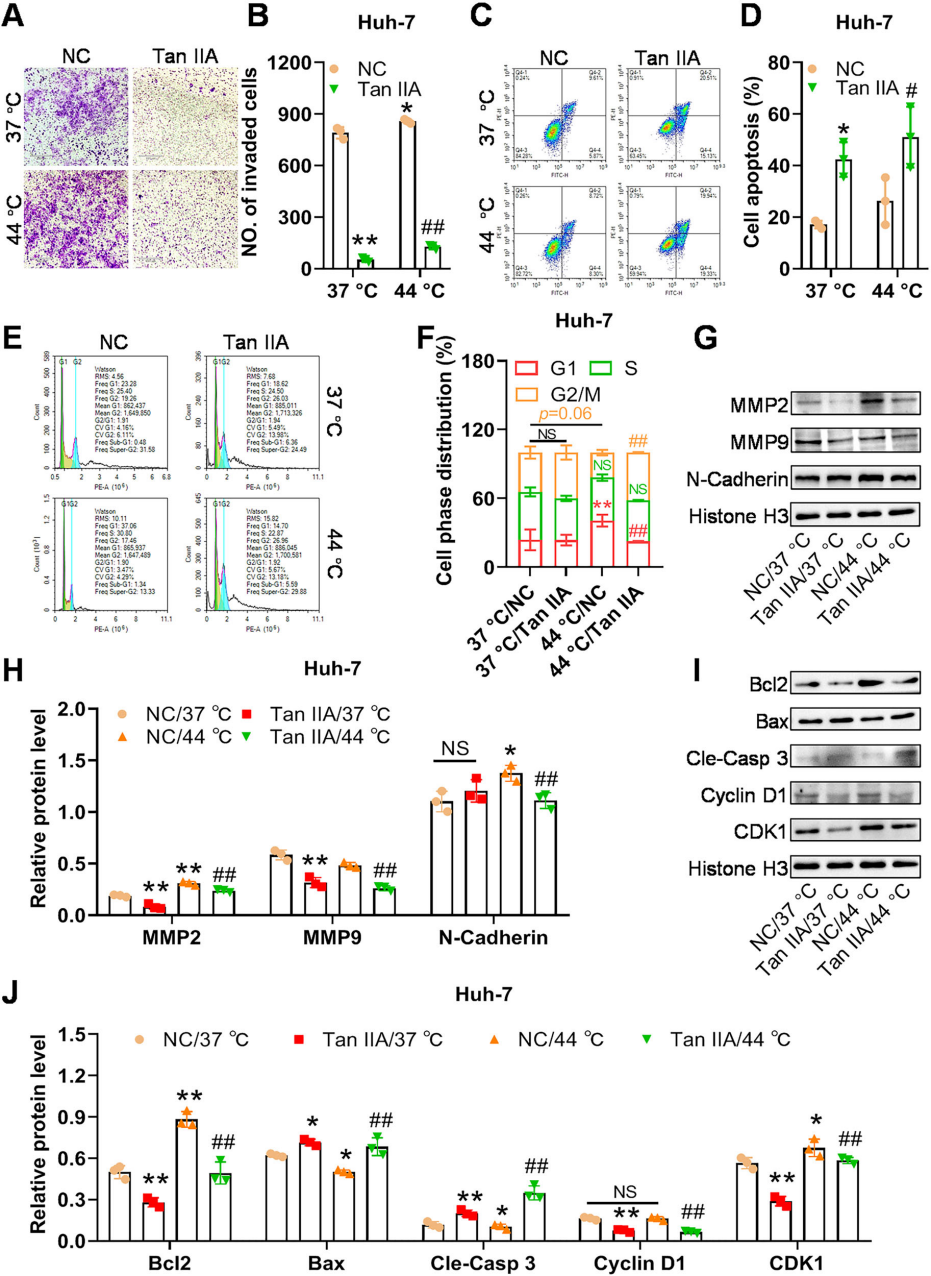
Tan IIA alleviates the heat-induced malignancy enhancement of HCC cells. **A**, **B** Heat pretreatment at 44 °C enhances the invasion of Huh-7 cells, and Tan IIA inhibits the invasion with or without heat pretreatment. **C**, **D** Heat pretreatment induces apoptosis of Huh-7 cells, and Tan IIA increases the apoptosis of the cells with or without the heat pretreatment. **E**, **F** Heat pretreatment increases the percentage of HCC cells in the G1 phase, and Tan IIA increases the cells in the G2/M phase, and decreases those in the G1 phase. **G**, **H** Heat pretreatment increases the protein level of the markers for cell invasion and/or EMT, MMP2 and N-cadherin, and Tan IIA decreases the protein levels of MMP-2/9 and N-cadherin. **I**, **J** Heat pretreatment increases the protein level of the anti-apoptotic marker Bcl2 and the G1-S transition markers cyclin D1 and CDK1, and decreases the apoptotic markers Bax and cleaved Caspase 3, whereas Tan IIA restores these effects. *p < 0.05, **p < 0.01, compared with NC at 37 °C; # *p* < 0.05, ## *p* < 0.05, compared with NC at 44 °C.

To explore the effects of the sublethal heat and Tan IIA on the function of HCC cells, we treated Huh-7 cells with the sublethal heat (44 °C) and/or Tan IIA and assessed changes in cell invasion, apoptosis, and cell cycle. The invasion capability of Huh-7 cells was moderately enhanced after induction by the sublethal heat (44 °C), and regardless of whether the sublethal heat was used as a stimulus, Tan IIA had a significant inhibitory effect on the invasion ability of Huh-7 cells (Fig. 2A, B). Induction at 44 °C appeared to lead to a certain degree of increased apoptosis, and the apoptotic induction ability of Tan IIA was not weakened by the sublethal heat stimulation (Fig. 2C, D). This result was not as expected, and we deemed that the injured cells could not be completely removed before reinoculation using simply removing the floating cells, thus leading to more apoptosis rather than less. Sublethal heat induction significantly increased the proportion of cells in the G1 phase, slightly (*p* = 0.06) decreased the proportion of cells in the G2 phase, but did not change that in the S phase (Fig. 2E, F). These results suggested that the sublethal heat has the ability to induce cell cycle arrest at G1/S transition, rather than promotes the cell division. Compared with the 44 °C/NC group, the 44 °C/Tan IIA group had a significantly lower percentage of cells in the G1 phase, and significantly higher percentage of cells in the G2/M phase (Fig. 2E, F), indicating a Tan IIA-induced a rescued G1/S transition, and a newly cell cycle arrest at G2/M transition or M phase. At the molecular level, after induction at 44 °C, the protein levels of MMP2, MMP9, and N-Cadherin were significantly increased; however, when Tan IIA was used to treat the heat-induced Huh-7 cells, the upregulation of MMP2, MMP9, and N-Cadherin was impeded (Fig. 2G, H). The sublethal heat also caused a significant decrease in Bax and cleaved Caspase 3, and a remarkable increase in Bcl2 protein levels (Fig. 2I, J), indicating a potential inhibitive effect of sublethal heat on the apoptosis of Huh-7 cells. The cyclin D1 protein level was not influenced, while the CDK1 protein level was slightly increased (*p* = 0.048) to some extent by sublethal heat (Fig. 2I, J), the latter of which was likely a contributor to cell cycle arrest at the G1 phase (Fig. 2E, F). When Tan IIA was applied, the protein levels of Bcl2, cyclin D1, and CDK1 were significantly decreased, and those of Bax and cleaved caspase 3 were significantly increased, regardless of pretreatment with heat or not (Fig. 2I, J). The reduction of CDK1 supported the result that G2/M cell cycle arrest, while the decrease in cyclin D1 was contrary to the lower number of cells at G1 phase, after Tan IIA treatment in the heated cells (Fig. 2E, F). This discrepancy indicated the participation of other undetected proteins that regulate cell cycle in heated Huh-7 cells after Tan IIA treatment. Additionally, it was observed that the classic cytoplasmic internal protein reference GAPHD or β-actin was significantly influenced by Tan IIA; therefore, stably expressed histone H3 was chosen to serve as the internal control for samples treated with Tan IIA. In summary, the sublethal heat impeded cell division, but enhanced the invasion, and had the potential to promote anti-apoptotic abilities of HCC cells, while Tan IIA exhibited a stable anti-tumor effect in HCC cells with or without heat induction.

### Tan IIA altered the gene expression profile in HCC cells and potentially targeted at ALDH7A1

**Fig. 3:**
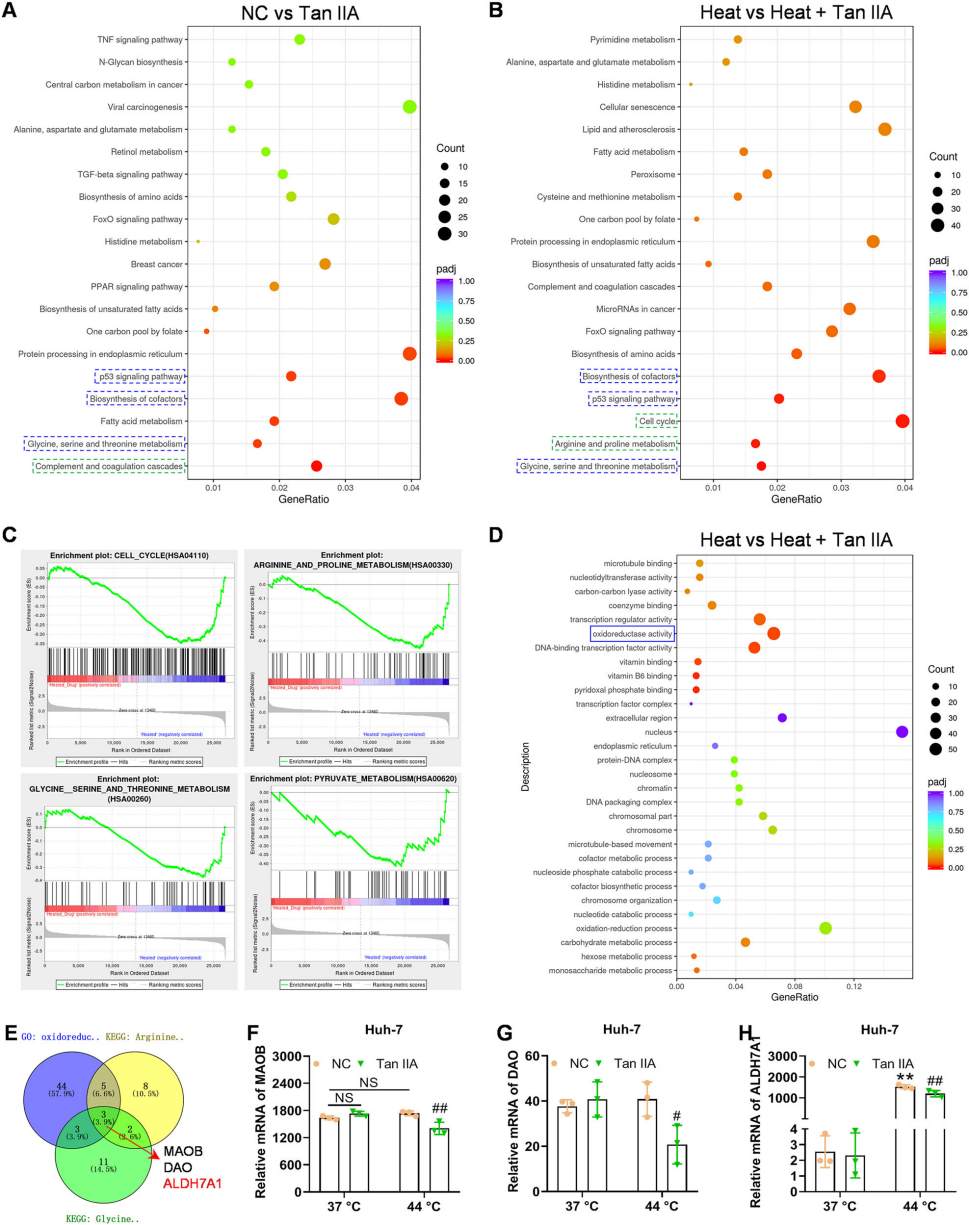
Bioinformatics after mRNA sequencing for Huh-7 cells indicate that oxidoreductase ALDH7A1 in amino acid metabolic pathways may play important roles in anti-HCC effects of Tan IIA after heat stress induction. KEGG pathway enrichment of DEGs between the NC and the Tan IIA groups (**A**), as well as the Heat and Heat + Tan IIA groups (**B**). The blue dashed boxes represent the pathways that are shared between the two comparisons, while the green dashed boxes represent pathways that are different from each other. **C** GSEA was conducted on 4 KEGG pathways (Cell cycle, Arginine and proline metabolism, Serine and threonine metabolism, and Pyruvate metabolism) in the comparison between the Heat and the Heat + Tan IIA groups. **D** GO enrichment of the DEGs between the Heat and the Heat + Tan IIA groups. The blue solid box indicates the most significantly enriched GO term. **E** The Venn diagram illustrates the overlaps among the three gene sets, which were significantly enriched in the two KEGG pathway terms (Arginine and proline metabolism, and Glycine, serine and threonine metabolism) and one GO term (Oxidoreductase activity). **F–H** The relative expression level (data extracted from the RNA-seq results) of the 3 chosen genes MAOB, DAO, and ALDH7A1 in heat-induced Huh-7 cells treated with Tan IIA. **p < 0.01, compared with NC at 37 °C; # *p* < 0.05, ## *p* < 0.05, compared with NC at 44 °C; NS not significant.

To reveal the potential mechanisms underlying the protumor effect of the sublethal heat and anti-tumor effect of Tan IIA on heat-induced HCC cells, we used RNA sequencing to analyze the gene expression after corresponding treatments. The results indicated that the differences induced by heat stimulation could be partially reversed by Tan IIA (white box area in Fig. S2A). Compared to Tan IIA group, the Heated+Tan IIA group exhibited some different profile (blue line box area) and some similar profile (Fig. S2A), indicating part different and part similar mechanisms of action of Tan IIA in heat-induced HCC cells. KEGG pathway enrichment analysis of the NC and heat groups revealed that heat stimulation may promote oxidative phosphorylation and ROS formation in Huh-7 cells (Fig. S2B). Tan IIA-altered genes were enriched in Biosynthesis of cofactors, p53 signaling pathway, and glycine serine and threonine metabolism pathways, regardless of whether Huh-7 cells are subjected to heat stimulation (blue dashed boxes, Fig. 3A, B). In heat-induced Huh-7 cells, Tan IIA significantly inhibited cell cycle (consistent with the results in Fig. 2E, F), arginine and proline metabolism, and glycine serine, and threonine metabolism pathways, which were supported by the findings in KEGG pathway analysis and GSEA (blue dashed boxes, Fig. 3B, C). Additionally, we noticed that pyruvate metabolism in heated Huh-7 cells was inhibited by Tan IIA, according to the GSEA results, and a significant enrichment of oxidoreductase activity was identified after GO enrichment analysis (Fig. 3C, D). By intersecting key gene sets from the arginine and proline metabolism, glycine serine and threonine metabolism pathways, and oxidoreductase activity, we identified three common genes, MAOB, DAO, and ALDH7A1 (Fig. 3E). Subsequently, we extracted the expression values of the three genes, MAOB, DAO, and ALDH7A1, from the RNA-seq results and displayed them as histograms (Fig. 3F–H). It is evident that ALDH7A1 expression significantly increased (600-fold) after heat induction at 44 °C, and Tan IIA effectively suppressed the transcriptional expression levels of ALDH7A1 in the sublethal heat-induced Huh-7 cells (Fig. 3F–H), suggesting its potential role in Tan IIA treatment for heat-induced HCC.

Additionally, RNA-sequencing and bioinformatics analysis were also conducted on Hep-G2 cells. The results showed that, compared to Huh-7 cells, Hep-G2 cells with or without heat pretreatment exhibited significantly different pathway enrichment results after Tan IIA treatment (Fig. S3A, B). Despite the significant cell cycle arrest in both Huh-7 and Hep-G2 cells following Tan IIA treatment, the GSEA results for the p53 pathway in TP53 mutant Huh-7 cells were not significant (data not shown), while in Hep-G2 cells carrying wild-type TP53, the p53 pathway showed a tendency towards inhibition (Fig. S3C). Moreover, the enrichment results for other pathways were also remarkably divergent between Huh-7 and Hep-G2 cells. The major GO enrichment results for differentially expressed genes in Tan IIA-treated heat-induced Hep-G2 cells mainly included transition metal binding, which was completely different from the results in Huh-7 cells (Figs. 3D and S3D). Therefore, the cell cycle inhibitory mechanisms of Tan IIA in the two cell lines are likely mediated by fundamentally different pathways. In addition, due to limited time and budget, we only further investigated Huh-7 cells in the following experiments of this study.

### The validation of Tan IIA’s potential target (ALDH7A1) and its function in heated Huh-7 cells

**Fig. 4:**
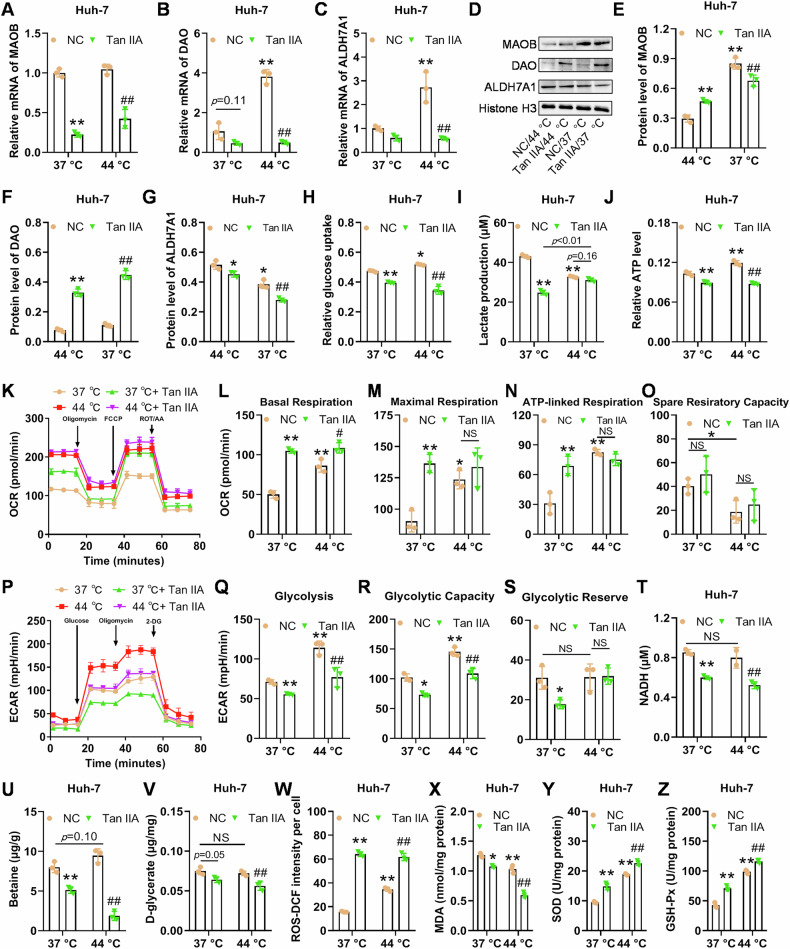
Tan IIA reduces ALDH7A1 and intracellular osmolytes and inhibits glycolysis in the heat stress-induced HCC cells. **A–C** The altered mRNA level of MAOB, DAO, and ALDH7A in Huh-7 cells with or without heat pretreatment validated using qPCR. **D–G** The altered protein level of MAOB, DAO and ALDH7A in Huh-7 cells with or without heat pretreatment validated using western blotting. **H–J** Heat pretreatment induces increased glucose uptake and ATP production, and decreased the production of lactate, and Tan IIA reduces these indices to various extents. **K–S** Seahorse analyses of oxygen consumption rate (OCR) and extracellular acidification rate (ECAR): heat-induced cells have higher OCR and ECAR values, and Tan IIA can enhance basal respiration and lower some ECAR values. **T** Tan IIA treatment results in the decrease in NADH production with or without heat pretreatment. **U**, **V** Tan IIA reduces the osmolytic and enzymatic products betaine and d-glycerate to various extents with or without heat pretreatment. **W–Z** Heat pretreatment and Tan IIA treatment induce ROS and inhibit LPO to various extents. *p < 0.05, **p < 0.01, compared with NC at 37 °C; # *p* < 0.05, ## *p* < 0.05, compared with NC at 44 °C; NS not significant.

To validate the expression of potential targets of Tan IIA, MAOB, DAO, and ALDH7A1, we induced Hun-7 cells at 44 °C and measured their transcription and protein levels. We found that induction at 44 °C significantly elevated the transcription levels of DAO and ALDH7A1, and Tan IIA was able to reverse their expression level (Fig. 4A–C). At the protein level, MAOB and DAO (normalized to histone H3 instead of GAPDH or β-actin, which has been explained above) were downregulated by induction at 44 °C, and they were significantly increased by Tan IIA in the cells induced at 44 °C (Fig. 4D–F). Tan IIA exhibited an inhibitory effect on ALDH7A1, regardless of heat induction at 44 °C (Fig. 4D, G). DAO and MAOB showed quite contradictory results at transcription and expression level, and therefore ALDH7A1 might warrant further exploration. Due to time and budget constraints, we did not further explore the changes of DAO and MAOB, and only chose to focus on ALDH7A1 for further investigation.

As mentioned above, bioinformatical results suggested that heat stimulation induced the enrichment of oxidative phosphorylation and chemical carcinogenesis-reactive oxygen species (ROS) in Huh-7 cells, and Tan IIA caused the inhibition of pyruvate metabolism in heated Huh-7 cells (Figs. S2B and 3C). Besides, ALDH7A1 essentially belongs to the aldehyde dehydrogenase (ALDH) family and is believed to be involved in osmoregulation by metabolizing various aldehyde substrates. These substrates include osmolyte precursors, such as d-glyceraldehyde, which, together with NAD^+^ and catalyzed by ALDH7A1, is converted into d-glycerate and NADH. d-glycerate serves as the direct precursor of osmolyte glucosylglycerate [17]. Betaine aldehyde and lipid peroxidation (LPO)-derived aldehydes are also the substrates of ALDH7A1 [18]. Therefore, we validated the following indices in the downstream experiments: A. cellular respiratory (mainly glycolysis) indices, including glucose intake, and production of ATP and lactate; B. redox indices, including ROS and LPO; and C. the products of ALDH7A-mediated enzymatic reaction, including betaine, d-glycerate, and NADH.

To assess the impact of Tan IIA on glycolysis after heat induction, we measured glucose uptake, lactate production, and ATP levels. The results showed an increase in glucose uptake in heat-induced Huh-7 cells, compared with NC group, but the increase could be effectively reversed by Tan IIA (Fig. 4H). Heat induction suppressed lactate production (potentially inhibiting glycolysis), and Tan IIA slightly (*p* = 0.163) weaken lactate production in cells after heat induction (Fig. 4I). The sublethal heat promoted ATP production, but Tan IIA significantly reduced ATP levels (Fig. 4J). Using Seahorse analyzer, we further showed that both heat stress and Tan IIA induced significantly higher basal respiration, maximal respiration, and ATP-linked respiration, however, the heat stress reduced, and Tan IIA did not influence spare respiratory capacity (Fig. 4K–O). We also evaluated the ECAR, and it was shown that heat induction resulted in higher glycolysis and glycolytic capacity, but Tan IIA was able to significantly inhibit them in the non-heated or heated cells (Fig. 4P–S). Additionally, the results indicated that Tan IIA significantly reduced NADH levels in cells with or without heat stimulation (Fig. 4T). These results indicated that Tan IIA can markedly inhibit cellular energy metabolism, and this is achieved through the inhibition of glycolysis. To assess the direct impact of Tan IIA on the function of ALDH7A1 after heat induction, we measured two products, betaine and d-glycerate, after intracellular ALDH7A activity. The results showed that the levels of betaine and d-glycerate were not significantly increased by the sublethal heat, but they were effectively reduced by Tan IIA in the cells pretreated by the sublethal heat (Fig. 4U, V). Furthermore, we investigate the effects of Tan IIA on ROS in the cells after heat induction. The results showed that heat induced more and relatively evenly distributed ROS, while Tan IIA significantly increased ROS, which was strongly stained in some of the cells instead of all (Fig. 4W and Supplementary Fig. S4A). Heat decreased MDA (a marker of lipid peroxidation) in Huh-7 cells, and Tan IIA decreased MDA in non-heated or heated Huh-7 cells (Fig. 4X).

### Knockdown of ALDH7A1 in Hun-7 cells inhibited glycolysis and sensitized the cells to hyperosmotic stress

**Fig. 5:**
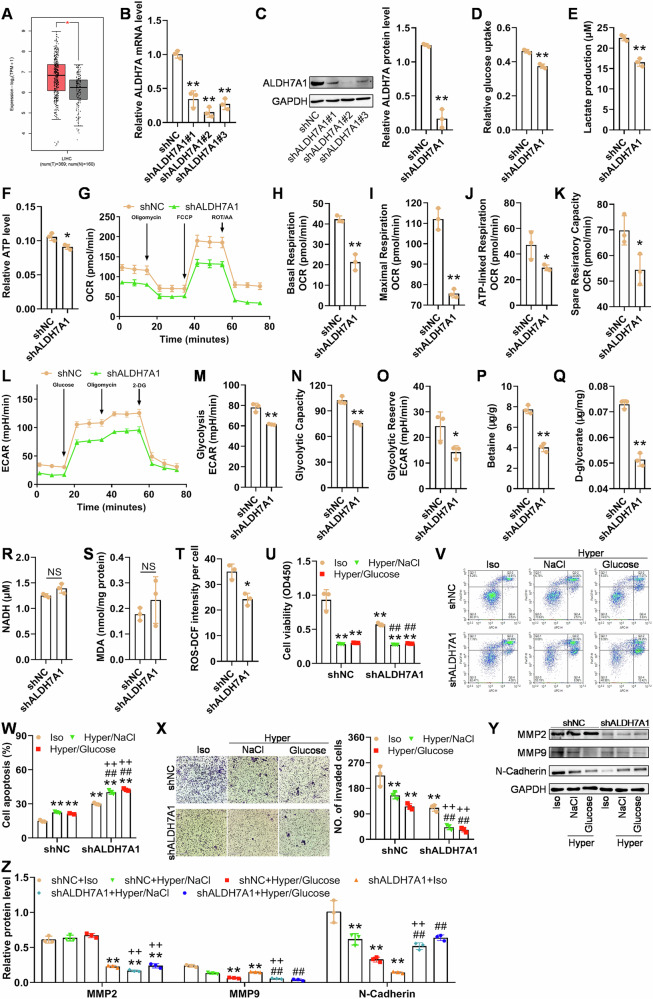
ALDH7A1 knockdown inhibits glycolysis of heat-induced HCC cells and sensitizes the cells to hyperosmotic stress. **A** ALDH7A1 is overexpressed at mRNA level in HCC tissues, compared with normal liver tissue (analyzed using GEPIA2). **B**, **C** Validation of the reduced expression of ALDH7A1 at mRNA and protein level after shRNA lentiviral infection using qPCR and western blotting. **D–F** Knockdown of ALDH7A1 inhibits glucose uptake, lactate production, and ATP production. **G–O** Seahorse analyses of OCR and ECAR: the cells with low level of ALDH7A1 have significantly decreased OCR and ECAR values. **P, Q** ALDH7A1 knockdown reduces its downstream osmolytic and enzymatic products betaine and d-glycerate. ALDH7A1 knockdown does not influence NADH (**R**) or LPO (**S**), but reduces ROS (**T**). For (**A–T**), *p < 0.05, **p < 0.01, compared with shNC; NS not significant. **U** ALDH7A1 knockdown inhibits the viability (at 72 h) of heat-induced Huh-7 cells in isosmotic or hyperosmotic conditions. Iso isosmotic, Hyper Hyperosmotic. **V–X** ALDH7A1 knockdown increases the sensitivity of heat-induced HCC cells to hyperosmotic stress: knockdown of ALDH7A1 results in more apoptotic cells and less invasive cell in NaCl- or glucose-mediated hyperosmotic conditions. **Y–Z** ALDH7A1 knockdown reduces MMP2, MMP9, and N-cadherin in isosmotic media, and only reduces MMP2 and MMP9 in NaCl-mediated hyperosmotic media. For **U–Z**, **p < 0.01, compared with shNC+Iso group; ## *p* < 0.01, compared with shALDH7A1+Iso group; ++ *p* < 0.01, compared with shNC+Hyper/NaCl or shNC+Hyper/Glucose group; NS not significant.

Through online bioinformatic analysis using GEPIA2 (http://gepia2.cancer-pku.cn/), we discovered a significant increase in ALDH7A1 mRNA level in HCC tissues compared to normal liver tissues (Fig. 5A). Subsequently, we knocked down ALDH7A1 and investigated its role in the survival of Hun-7 cells following heat induction. We identified the most efficient shRNA (shALDH7A1#2) for further studies (Fig. 5B, C). Following ALDH7A1 knockdown, glucose uptake, lactate levels, and ATP production significantly decreased (Fig. 5D–F). Using Seahorse XF Analyzer, we further characterized the oxidative phosphorylation and glycolysis in the Huh-7 cells with low level of ALDH7A1. As shown in Fig. 5G–O, the knockdown of ALDH7A1 resulted in significantly lower basal respiration, maximal respiration, ATP-linked respiration, spare resiratory capacity, glycolysis, glycolytic capacity, and glycolytic reserve. ALDH7A1 knockdown resulted in a decrease in the levels of downstream osmolytes betaine and d-glycerate (Fig. 5P-Q). The above results were consistent with the results observed after Tan IIA treatment (Fig. 4T–V). ALDH7A1 knockdown caused insignificant changes in NADH (Fig. 5R) and MDA (Fig. 5S) but a slight increase in ROS levels (Fig. 5T). This discrepancy with the results after Tan IIA treatment may be due to the existence of other targets of Tan IIA that can influence ROS and LPO.

Downregulation of ALDH7A1 in Hun-7 cells led to a significant decrease in cell viability under hyperosmotic conditions, regardless of whether ALDH7A1 expression was knocked down (Fig. 5U). Both ALDH7A1 knockdown and hyperosmotic conditions substantially increased apoptosis levels in heat-induced Huh-7 cells, and in the cells with low level of ALDH7A1 were more sensitive to hyperosmotic stress (Fig. 5V–W). The invasive capacity of cells significantly decreased after ALDH7A1 knockdown or in hyperosmotic conditions, regardless of ALDH7A1 expression, but cells with knocked down ALDH7A1 exhibited weaker invasion under hyperosmotic conditions (Fig. 5X). Compared to the cells infected with shNC, the cells with downregulated ALDH7A1 showed significant reductions in MMP2, MMP9, and N-cadherin. When the cells with ALDH7A1 knockdown were exposed to hyperosmotic conditions, the change in MMP2 was not significant (possibly due to decreased sensitivity to hyperosmotic stress after ALDH7A1 knockdown), while MMP9 was significantly downregulated, and N-cadherin was simultaneously upregulated under hyperosmotic conditions (EMT and invasion were not simultaneously influenced by hyperosmotic stress; Fig. 5Y, Z). These results indicated that hyperosmotic stress-induced apoptosis and inhibition of invasion could be reinforced by knocking down ALDH7A1.

### Overexpression of ALDH7A1 in Hun-7 cells promoted glycolysis and increased cell resistance to hyperosmotic stress

**Fig. 6:**
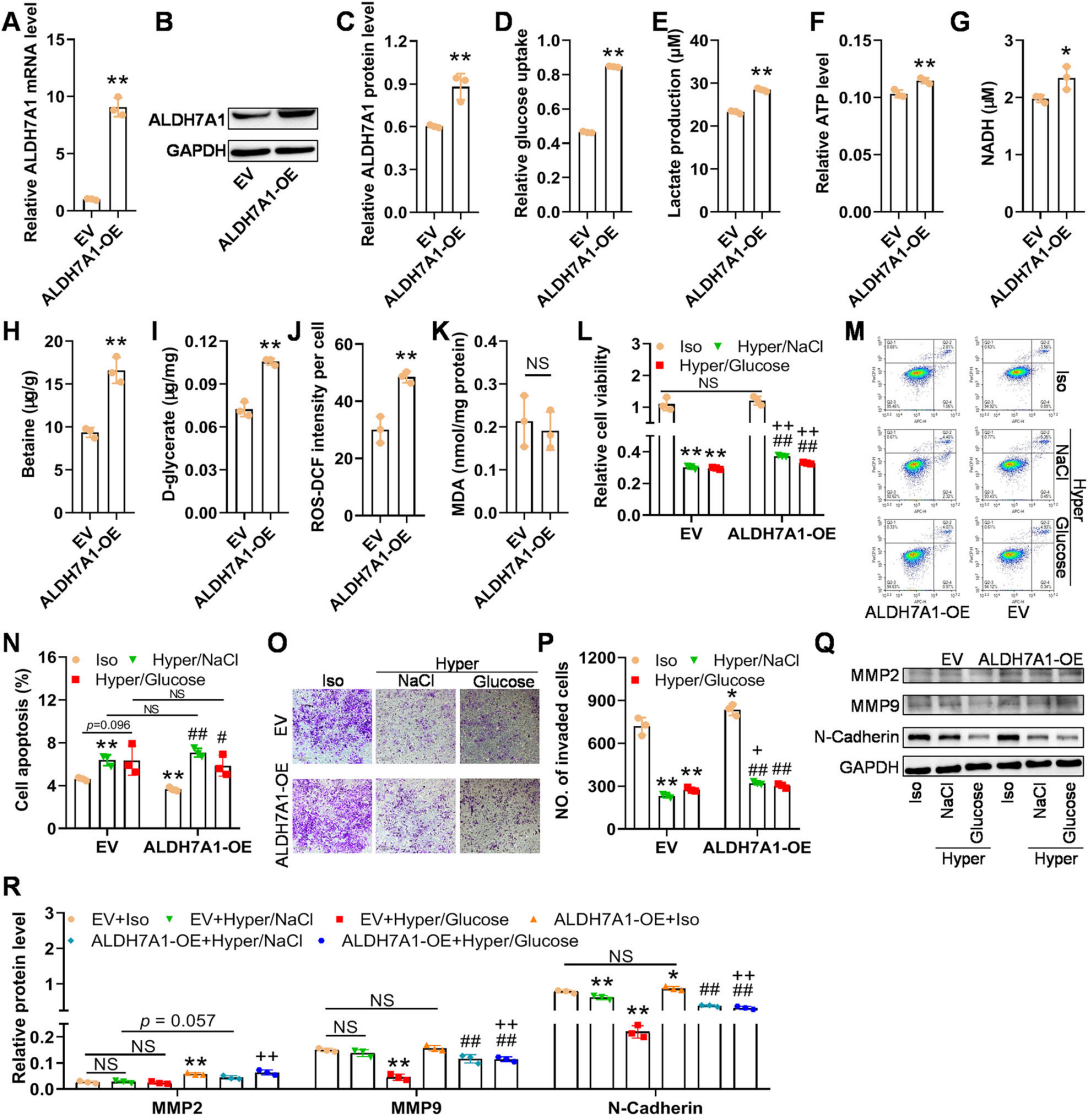
ALDH7A1 overexpression promotes glycolysis of heat-induced HCC cells and protects the cells against hyperosmotic stress. **A–C** Validation of ALDH7A1 OE at mRNA and protein levels using qPCR and western blotting. ALDH7A1 OE promotes glucose uptake (**D**), lactate production (**E**), and ATP level **F** in the heated HCC cells. ALDH7A1 OE increases the levels of its enzymatic and/or osmolytic products NADH (**G**), betaine (**H**) and d-glycerate (**I**). **J, K** ALDH7A1 OE enhances its downstream ROS but does not influence lipid peroxidation. In (**A–K**), *p < 0.05, **p < 0.01, compared with EV (empty vector) group. **L** Hyperosmotic stress inhibits the viability of the cells with or without ALDH7A1 OE, and ALDH7A1 OE enhances the viability of heat-induced HCC cells in hyperosmotic culture media. **M**, **N** ALDH7A1 OE does not significantly inhibit hyperosmotic stress-induced apoptosis of HCC cells pretreated with heat. **O**–**R** ALDH7A1 OE promotes invasion and EMT of the heat-induced HCC cells under hyperosmotic stress. Iso, isosmotic; Hyper, Hyperosmotic. In (**L**–**R**), *p < 0.05, **p < 0.01, compared with EV+Iso group; # *p* < 0.05, ## *p* < 0.01, compared with ALDH7A1-OE+Iso group; + *p* < 0.05, ++ *p* < 0.01, compared with EV+Hyper/NaCl or EV+Hyper/Glucose group.

Furthermore, we overexpressed ALDH7A1 and studied its function in Hun-7 cells survived after heat induction. The significantly increased mRNA (Fig. 6A) and protein levels (Fig. 6B, C) of ALDH7A1 indicated the success of ALDH7A1 overexpression, following which, glucose uptake, lactate production, ATP production, and NADH production were all significantly increased (Fig. 6D–G). Therefore, ALDH7A1 overexpression enhanced glycolysis of heat-induced Huh-7 cells. The hyperosmotic regulation molecules betaine and d-glycerate were also significantly increased after ALDH7A1 overexpression (Fig. 6H, I). Unexpectedly, ALDH7A1 overexpression significantly increased ROS levels, but had no effect on MDA levels (Fig. 6J, K, Supplementary Fig. S4B).

Stable ALDH7A1 overexpression did not significantly increase the viability of HCC cells in isosmotic culture media. Hyperosmotic stress mediated by high concentration of NaCl or glucose resulted in significant inhibition of cell viability in heated cells with or without stable ALDH7A1 overexpression. Simultaneously, under hyperosmotic stress, Huh-7 cells overexpressing ALDH7A1 showed higher viability than Huh-7 cells (Fig. 6L). Similarly, in the Huh-7 cells infected with EV, hyperosmotic stress induced by NaCl and glucose led to a slight increase in cell apoptosis (*p* ≈ 0.11, vs EV+Iso group), and in the cells overexpressing ALDH7A1, hyperosmotic conditions mediated by NaCl and glucose significantly increased the proportion of apoptotic cells (*p* < 0.01, vs ALDH7A1-OE+Iso group, namely the cells with ALDH7A1 overexpression cultured in isosmotic media, Fig. 6M, N). ALDH7A1 overexpression didn’t show the ability against hyperosmotic stress-induced cell apoptosis, either. ALDH7A1 overexpression has a promoting effect on cell invasion, but hyperosmotic conditions significantly inhibited cell invasion, regardless of ALDH7A1 overexpression. However, in NaCl-mediated hyperosmotic surroundings, cells with ALDH7A1 overexpression seemed to have stronger invasion than control cells (*p* < 0.05, EV+Hyper/NaCl vs ALDH7A1-OE+Hyper/NaCl; Fig. 6O, P). At the molecular level, the protein levels of MMP2 and N-cadherin significantly increased after ALDH7A1 overexpression, but changes in MMP-9 were not significant (Fig. 6Q, R). In cells overexpressing ALDH7A1, NaCl-mediated hyperosmotic stress did not significantly change MMP2 but significantly downregulated MMP9 and N-cadherin. In addition, in NaCl- or glucose-mediated hyperosmotic culture conditions, ALDH7A1-OE cells seemed to have higher protein level of MMP2/9 and N-cadherin (Fig. 6Q, R).

The above results demonstrated that ALDH7A1 overexpression can promote glycolysis and resist hyperosmotic stress-induced inhibition of invasion, but is not able to effectively conquer the hyperosmotic stress-induced apoptosis.

### Tan IIA and ALDH7A1 specific inhibitor DEAB treatments caused similar gene expression pattern, and ALDH7A1 mediated the effects of Tan IIA on heated cells under hyperosmotic stress

**Fig. 7:**
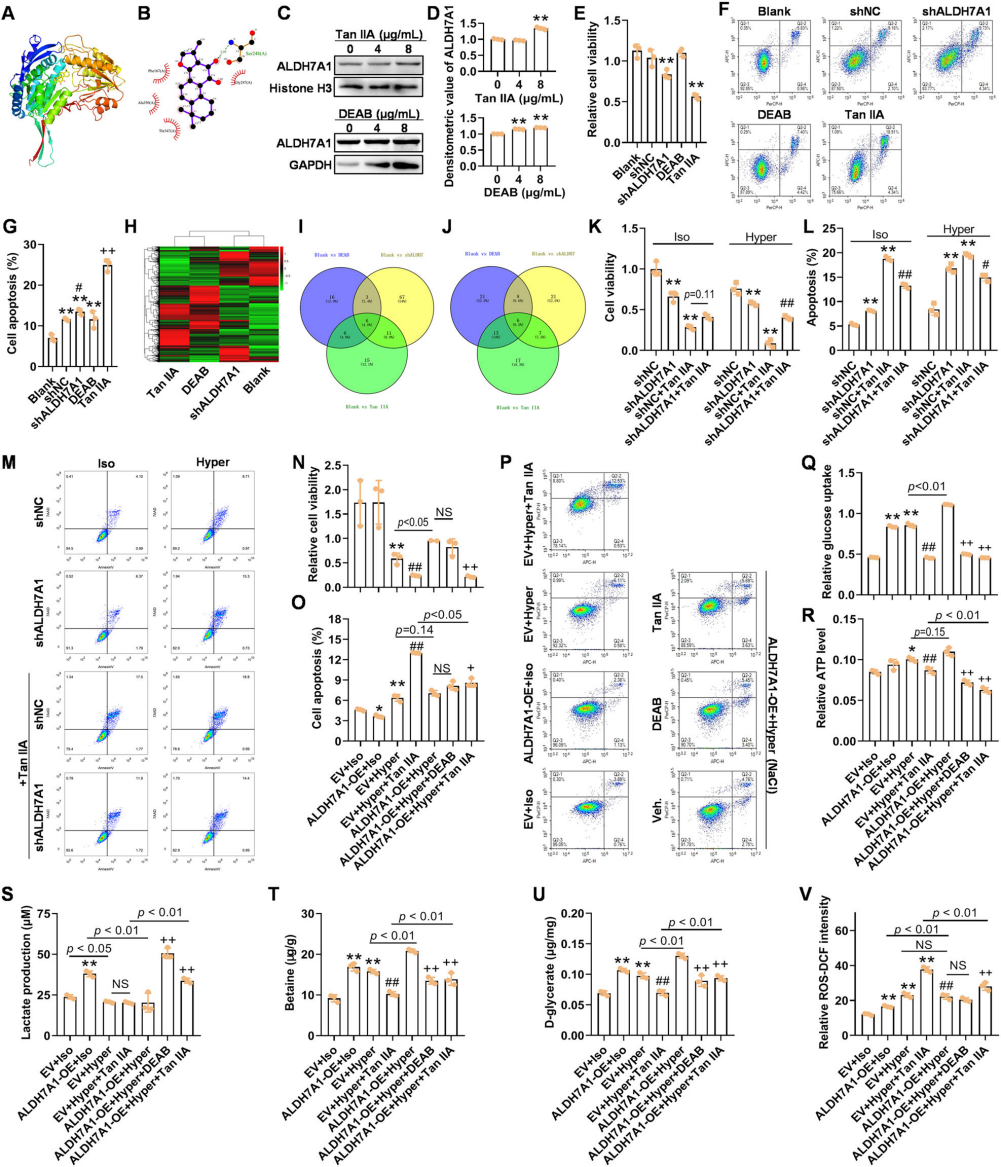
Tan IIA increases the sensitivity of heat-induced Huh-7 cells to hyperosmotic stress and inhibits glycolysis by deactivating ALDH7A1. Visualization of the molecular docking between Tan IIA and ALDH7A1 protein using PyMOL 2.3.0 (**A**) and LigPlot v2.2.8 (**B**). **C**, **D** The direct interaction between ALDH7A1 and Tan IIA or 4-diethylaminobenzaldehyde (DEAB, positive control) tested using cellular thermal shift assay (CETSA). **p < 0.01, vs 0 μg/mL. Viability (**E**) and apoptosis (**F**, **G**) of heat-induced Huh-7 cells, respectively, treated with shALDH7A1 lentivirus, DEAB, and Tan IIA for 48 h. **H**–**J** Comparison of gene expression of heat-induced Huh-7 cells, respectively, treated with shALDH7A1, DEAB, and Tan IIA, and the intersection of the corresponding GO or KEGG pathway enrichment results: DEG expression profile (**H**), Venn diagrams of GO (**I**) or KEGG pathway (**J**) terms. **p < 0.01, compared with Blank; # *p* < 0.05, compared with shNC; + *p* < 0.05, compared with shALDH7A1. **K**–**M** The anti-viability and proapoptotic effects of Tan IIA on the cells with scarce ALDH7A1 are attenuated. **p < 0.01, compared with shNC in Iso or Hyper group; # *p* < 0.05, compared with shNC+Tan IIA. **N**–**P** NaCl-mediated hyperosmotic stress inhibits viability and induces apoptosis, and Tan IIA further inhibits viability of heat-induced Huh-7 cells with or without ALDH7A1 OE in the presence of NaCl-mediated hyperosmotic stress. **Q**, **R** Hyperosmotic stress increases glucose uptake and ATP production, and Tan IIA reduces them in heat-induced Huh-7 cells with or without ALDH7A1 OE in the presence of hyperosmotic stress. **S** Tan IIA promotes lactate production in heat-induced Huh-7 cells overexpressing ALDH7A1 in the presence of hyperosmotic stress, and heat-induced cells overexpressing ALDH7A1 produces more lactate after Tan IIA treatment in hyperosmotic culture media. **T**, **U** Hyperosmotic stress induces betaine and d-glycerate, which can be enhanced by ALDH7A1 OE, and Tan IIA blocks the induction. **S** Hyperosmotic stress increases ROS level in heat-induced cells, and Tan IIA causes excessive ROS. For (**N**–**V**), ** *p* < 0.01, compared with EV+Iso; # *p* < 0.05, ## *p* < 0.01, compared with EV+Hyper; + *p* < 0.05, compared with ALDH7A1-OE+Hyper; NS not significant.

We further validated the potential interaction between Tan IIA and ALDH7A1 protein using molecular docking techniques and CETSA experiments. The binding energy between Tan IIA and ALDH7A1 was calculated to be −8.1 kcal/mol, demonstrating a strong binding interaction. Interaction between the small molecule and ALDH7A1 protein mainly occurred through hydrogen bonding and hydrophobic interactions, forming hydrogen bonds with Ser248 with a bond length of 3.09 Å; and hydrophobic interactions with Gly247, Phe167, Ala350, and Thr347 (Fig. 7A, B). CETSA results showed that the binding of Tan IIA to ALDH7A1 was concentration-dependent, similar to the known ALDH7A1 inhibitor DEAB. However, DEAB also appeared to bound to GAPDH, indicating nonspecific interactions beyond the ALDH enzyme family (Fig. 7C, D). Both ALDH7A1 shRNA and Tan IIA treatment significantly reduced the viability and increased apoptosis level of HCC cells. However, the changes in cell viability and apoptosis levels after DEAB treatment (8 μg/mL) were not significant, possibly owing to its lower concentration and lack of expected specificity (Fig. 7E–G).

At transcriptomic level, the expression profiles of the Tan IIA treatment group and the DEAB treatment group were quite similar (Figs. 7H; S5A and Supplementary Table S2). Among the genes regulated by shALDH7A1 (709 upregulated, 235 downregulated), 34.7% were also regulated by Tan IIA (Fig. S5B and Supplementary Table S2). Similarly, among the genes regulated by DEAB (1437 upregulated, 727 downregulated), 54.5% were also regulated by Tan IIA (Fig. S5C and Supplementary Table S2). These results indirectly indicated that Tan IIA likely directly targets the ALDH7A1 protein as well as other molecules. After GO or KEGG pathway enrichment analyses, we compared the results among different comparisons. There were a certain proportion of GO or KEGG pathway terms were overlapped (Fig. 7I, J). The 6 common GO terms were transporter activity, channel activity, passive transmembrane transporter activity, G-protein-coupled receptor activity, cyclic-nucleotide phosphodiesterase activity, and 3‘,5’-cyclic-nucleotide phosphodiesterase activity; and the 6 intersected KEGG pathway terms were Neuroactive ligand-receptor interaction, Insulin resistance, cAMP signaling pathway, Cytokine-cytokine receptor interaction, FoxO signaling pathway, and Insulin secretion (Fig. 7I, J and supplementary Table S1). The detailed enriched GO or KEGG pathway terms were shown in Supplementary Table S2 and partly visualized in Fig. S5D–I.

To verify the mediating role of ALDH7A1, we tested the effects of Tan IIA in ALDH7A1-knocked down cells. In isosmotic or hyperosmotic medium, cells with reduced ALDH7A1 expression exhibited relatively lower viability compared to the shNC-transfected group. After treatment with Tan IIA, the shALDH7A1-transfected cells in isosmotic medium showed a slightly (*p* = 0.11) higher viability than shNC cells, while in hyperosmotic medium, they showed a significantly (*p* < 0.01) higher viability (Fig. 7K). Consistently, shALDH7A1-transfected cells had fewer apoptotic cells than shNC cells after Tan IIA treatment, indicating reduced sensitivity to Tan IIA in both isosmotic and hyperosmotic conditions (Fig. 7L, M). These results suggest that ALDH7A1 deficiency compromises the anti-tumor effects of Tan IIA, indicating that Tan IIA inhibits Huh-7 cells at least in part by targeting ALDH7A1.

In isosmotic media, the enhancing effect of ALDH7A1 overexpression on cell viability was not significant, compared with EV-infected cells (similar to Fig. 6L). But in hyperosmotic culture, ALDH7A-OE had the potential to increase the viability of Huh-7 cells (Fig. 7N). Tan IIA but not DEAB could reduce the increase in activity caused by ALDH7A1 overexpression (*p* < 0.05, Fig. 7N). ALDH7A1 overexpression caused lower (*p* = 0.037) spontaneous cell apoptosis in isosmotic medium, and it did not show enhanced resistance of HCC cells to hyperosmotic stress (Fig. 7O, P). Under high osmotic conditions, after the cells with overexpressed ALDH7A1 underwent Tan IIA treatment, we observed a higher apoptotic level than that of untreated cells (ALDH7A1-OE+Hyper group), indicating that Tan IIA can effectively induce apoptosis in Huh-7 cells with high ALDH7A1 expression under NaCl mediated hyperosmotic conditions (Fig. 7O, P). Compared with EV+Hyper+Tan IIA group, ALDH7A-OE+Hyper+Tan IIA had less apoptotic cells, indicating that ALDH7A1 overexpression partly reversed Tan IIA-induced apoptosis (Fig. 7O, P).

In isosmotic culture media, cells overexpressing ALDH7A1 have enhanced glycolysis. NaCl-induced hyperosmotic stress led to a significant increase in glucose uptake and ATP production, with no significant change in lactate production (Fig. 7Q–S). Cells with ALDH7A1 overexpression showed a significant increase in glucose uptake and ATP production, with a decrease in lactate production under hyperosmotic conditions, compared with EV+Hyper group. However, overexpression of ALDH7A1 did not exhibit a significant increase in glycolytic capacity in hyperosmotic culture media. Under the effect of Tan IIA, glycolysis decreased significantly in cells overexpressing ALDH7A1 under hyperosmotic conditions, similar to the action of the positive control DEAB (Fig. 7Q–S). Overexpression of ALDH7A1 or exposure to hyperosmotic conditions (NaCl) led to a significant increase in betaine and d-glycerate levels in Hun-7 cells; further, in cells overexpressing ALDH7A1, hyperosmotic stress induced a further increase in levels for betaine and d-glycerate. Under the effect of Tan IIA, cells overexpressing ALDH7A1 or not showed significant reductions in betaine and d-glycerate levels under hyperosmotic conditions, similar to the effects of the positive control DEAB (Fig. 7T–U). Compared with EV+Hyper+Tan IIA, cells with ALDH7A1 OE in hyperosmotic NaCl had higher levels of betaine and d-glycerate, suggesting that ALDH7A1 OE can resist the antagonistic effects of Tan IIA one ALDH7A1. Both ALDH7A1 OE and hyperosmotic stress increased ROS level in heated Huh-7 cells, but the morphology of the staining results significantly varied (Fig. 7V and Supplementary Fig. S4C). Tan IIA effectively increased ROS levels in heated Huh-7 cells under hyperosmotic conditions (Fig. 7V and Supplementary Fig. S4C). In hyperosmotic culture media, ROS in ALDH7A1-OE cells after Tan IIA treatment had low ROS, compared with EV+Hyper+Tan IIA group. These results demonstrate that Tan IIA in combination with hyperosmotic stress can significantly sensitize heated cells, and Tan IIA inhibits glycolysis, and break balances of osmosis and ROS by inhibiting ALDH7A1.

### Combination of Tan IIA and hyperosmotic stress effectively inhibited tumor growth in mice bearing heat-induced Huh-7 cells

**Fig. 8:**
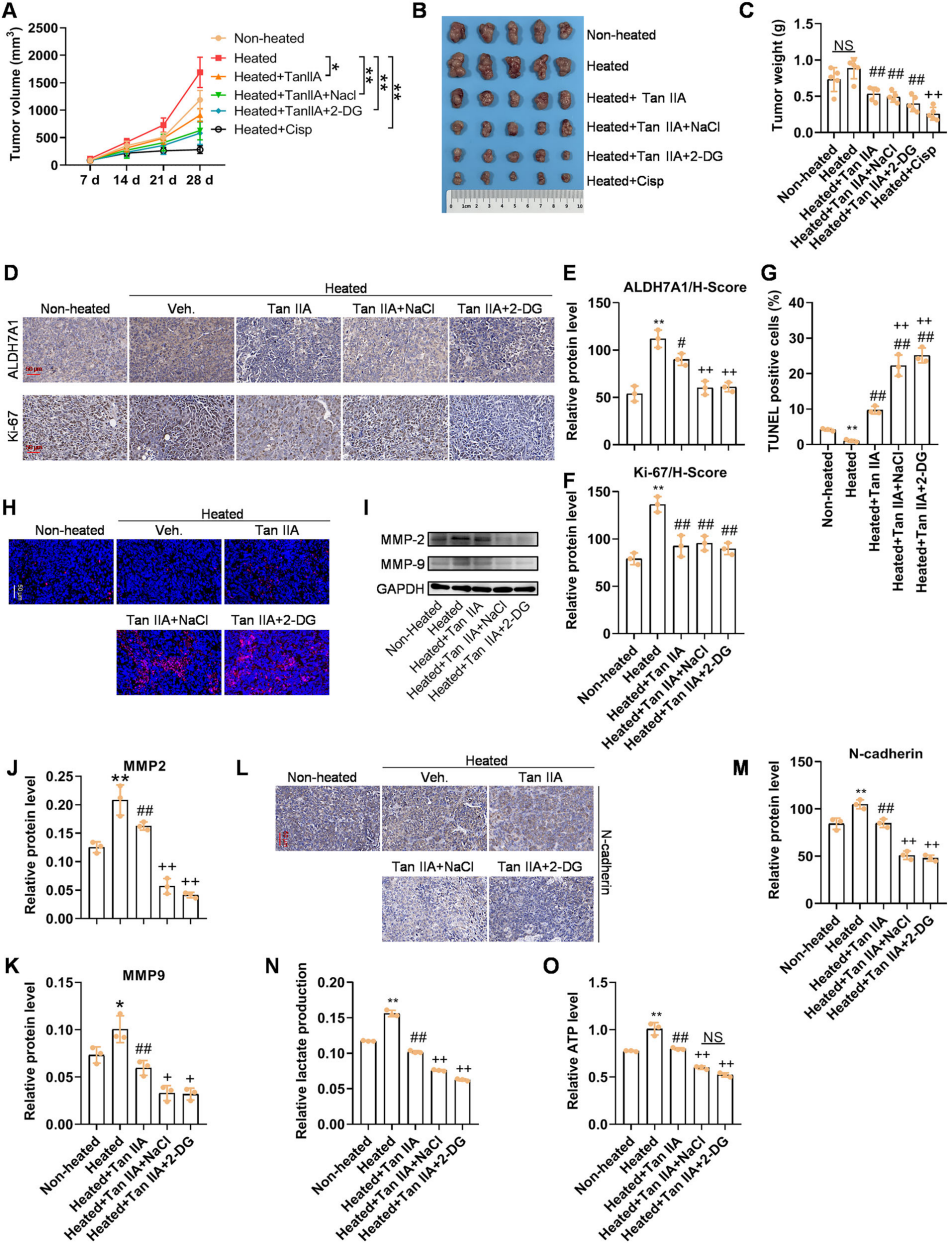
Hyperosmotic NaCl or glycolysis inhibitor 2-DG increases the sensitivity of heat-induced HCC cells to Tan IIA. **A** Heat-induced increase in tumor size can be slowed down by Tan IIA with or without NaCl-mediated hyperosmotic stress or the glycolysis inhibitor 2-DG. * *p* < 0.05, ** *p* < 0.01. **B**, **C** Gross observation and tumor weight after various treatments for 21 days: Tan IIA with NaCl or 2-DG has better therapeutic efficacy. **D–F** IHC of ALDH7A1 and Ki67: Tan IIA with NaCl or 2-DG results in a lower protein level of ALDH7A1 and Ki-67. **G**, **H** TUNEL staining and the corresponding quantitative analysis: Tan IIA with NaCl or 2-DG leads to more apoptotic cells than Tan IIA alone. **I–M** Western blotting and IHC results of invasion and/or EMT markers MMP2/9 and N-cadherin: the increased protein level of MMP2/9 and N-cadherin induced by heat can be blocked by Tan IIA, and its combination with NaCl or 2-DG has better inhibitive effects on MMP2/9 and N-cadherin. **N**, **O** Heat boosts lactate and ATP production, while Tan IIA, particularly when combined with NaCl or 2-DG, significantly inhibits them. For (**C–O**), *p < 0.05, **p < 0.01, compared with Non-heated; # *p* < 0.05, ## *p* < 0.01, compared with Heated; + *p* < 0.05, ++ *p* < 0.01, compared with Heated+Tan IIA; NS not significant.

The growth rate of cells surviving heat stimulation after being implanted into nude mice increased significantly. Although the p-values of the statistical results were all greater than 0.05, this growth trend was evident, with p-values gradually approaching 0.05. On day 28, the tumor volume and weight of heat-induced HCC cells treated with Tan IIA were significantly lower than those of the heat stimulation only group (*p* < 0.05). The inhibitory effect of Tan IIA was even more pronounced under conditions of increased osmotic pressure with NaCl (*p* < 0.01). 2-DG also enhanced the inhibitory effect of Tan IIA on the growth of heat-induced HCC to a certain extent (Fig. 8A–C). The tumor volume after treatments with Tan IIA+NaCl or Tan IIA + 2-DG for 28 days was not significantly different from that after treatment with positive control (Cisp, cisplatin). Immunohistochemical results showed that heat stimulation significantly increased the levels of ALDH7A1 and Ki-67 in the mice, which were suppressed by Tan IIA. Moreover, the inhibitory effect of Tan IIA was more pronounced under conditions of increased osmotic pressure with NaCl after heat stimulation. 2-DG also enhanced the inhibitory effect of Tan IIA on ALDH7A1 and Ki-67 levels after heat stimulation to a certain extent (Fig. 8D–F). TUNEL experiments and detection of MMP2 and MMP9 protein levels demonstrated that Tan IIA inhibited the enhanced effects of heat stimulation. Furthermore, the inhibition effect of Tan IIA was more pronounced under conditions of increased osmotic pressure with NaCl after heat stimulation. The lactate and ATP levels significantly increased after heat stimulation, which were effectively inhibited by Tan IIA. The inhibitory effect was even stronger when Tan IIA was combined with NaCl treatment, approaching the effect of the combination therapy of Tan IIA and 2-DG (Fig. 8O, P). These results confirm the ability of Tan IIA to inhibit the growth of heat-induced HCC cells in mice, with a stronger effect when combined with NaCl treatment.

## Discussion

It’s known that thermal ablative methods, such as radiofrequency ablation (RFA) and microwave ablation (MWA), have been widely chosen as curative treatment options in many HCC treatment guidelines because of their better clinical outcomes and minimally invasive procedure [19]. However, moderate hyperthermia enhances the tumor cell viability, survival, and invasive potential of HCC cells, and clinically, incomplete ablation is associated with high risk of tumor progression [20]. This paradoxical effect underscores the complexity of hyperthermic therapy and suggests that temperature modulation must be carefully optimized to avoid inadvertently promoting tumor progression, and to avoid the situation that tumor cells still exist after ablation and their malignancy is enhanced, preventions or interventions must be applied. To serve this purpose, we propose a potentially effective strategy that Tan IIA in combination with hyperosmotic NaCl, following or during the thermal ablation for HCC therapy. Underlying this method is that Tan IIA at least partly targets at ALDH7A1 and inhibits its enzymatic activity, decreasing the intracellular level of NADH and the osmolytic products, betaine, glycerate, etc., which further interfere with glycolysis, breaks the osmotic balance, and result in tumor growth arrest and/or cell apoptosis.

According to the literature, heat at a certain range often induces tumor growth of HCC by activating MET and EGFR signals [21], extracellular collagen I-mediated ERK signal [22], or VEGF/VEGFR1 signal [5]. In our study, we show distinct results in Huh-7 cells after heat induction that oxidative phosphorylation and chemical carcinogenesis-ROS pathways (Fig. S2B) are activated instead of the above signals, which is also quite different from the results in Hep-G2 cells (Fig. S3A). The difference may be resulted from p53 mutation, because p53 mutation is a main and essential difference between Huh-7 and Hep-G2 or other cells. However, further investigation is needed to validate the p53 role in mediating the heat-induced promotion of HCC cells. Despite the mutation of p53, Tan IIA showed potent inhibitive efficacy on tumor cell viability after heat induction in this study.

In heated Huh-7 cells, we further found the inhibited cell invasion and promoted apoptosis of Huh-7 cells after Tan IIA treatment. The molecular basis for Tan IIA’s efficacy lies on its ability to downregulate ALDH7A1 mRNA and inhibit enzymatic activity. It’s a key enzyme involved in the efficient metabolism of aldehydes, including the osmolyte precursor, betaine aldehyde, lipid peroxidation-derived aldehydes, and intermediate lysine degradation product α-aminoadipic semialdehyde (α-AASA), and the regulation of oxidative stress and cellular osmosis [18, 23]. According to The Human Protein Atlas (https://www.proteinatlas.org/), ALDH7A1 is specifically expressed in hepatocytes. It’s subcellularly localized in cytosol, nucleus, and mitochondria [18]. Using GEPIA2, we noticed that ALDH7A1 mRNA is higher (Fig. 5A) in HCC tissues than normal liver tissues, including tumor-adjacent tissues from TCGA and normal liver tissues from GTEx database, but its mRNA level decreases as tumor stage increases (data not shown). Data from cProSite (https://cprosite.ccr.cancer.gov/) indicated that the protein level of ALDH7A1 is lower in the HCC tissues, compared with adjacent normal tissue (data not shown). The discrepancy between mRNA and protein levels when comparing normal liver to HCC may result from abnormally enhanced protein degradation of ALDH7A1 or increased stability of ALDH7A1 mRNA post-transcription in HCC. Similarly, a study pointed out low protein level of ALDH7A1 in HCC is associated with the patients’ poor clinical outcome [24]. As for other types of cancer, a high level of activity of this enzyme has been proven to be involved in the formation of prostate cancer bone metastases [25]. It is also associated with stronger resistance of lung cancer cells against anticancer drugs like gemcitabine [26] and a higher incidence of lung cancer recurrence after surgical resection [27]. Additionally, it is linked to tumor formation and poor prognosis in pancreatic ductal adenocarcinoma [28]. Our results in this study are consistent with these findings in other cancer types, but seemingly contrary to the aforesaid results in HCC. According to the literature, in HPV16^+^/p53WT HNSCC, where wild type p53 is inactivated by the HPV oncogene, E6, high ALDH7A1 correlated with poor prognosis [29], indicating that p53 functional status is an important factor influencing the association between ALDH7A1 expression and patient prognosis. Therefore, our results based on TP53-mutant Huh-7 cells also suggest that ALDH7A1 is conducive to tumor progression in TP53 mutant HCC, and the unknown TP53 status in the clinical samples may lead to the discrepant findings.

Under normal conditions, ALDH7A1 is dispensable in HEK293 cells or HeLa cells. However, under hypoxia, NADH generated by ALDH7A1 can balance cellular energy, and when ALDH7A was knocked down by its specific siRNA under hypoxia, ATP production significantly reduced [30]. In our study, we observed that the mRNA level of ALDH7A1 was tremendously increased (600-fold) by heat at 44 °C (Fig. 3H), consistently implying its potentially important role under heat stress. It is known that ALDH7A1 promotes the following biochemical reactions: d-glyceraldehyde + NAD^+^ + H_2_O = d-glycerate + NADH + 2H^+^, and Fatty aldehyde + NAD^+^ + H_2_O = Fatty acid + NADH + 2H^+^, from which we can see that NADH is a frequently produced product of ALDH7A1 in addition to the osmolyte d-glycerate. ALDH7A1 has been identified as a glycolysis-related gene in cancer, which may facilitate glycolysis and promote cell survival under stress conditions [30–32]. In addition, increased NADH by ALDH7A1 overexpression results in the reduced cytosolic NAD^+^/NADH ratio, which means the glycolysis can be possibly suppressed [33]. Simultaneously, the reduction of the NAD^+^/NADH ratio mediated by mitochondrial ALDH7A also theoretically occurs, meaning the oxidative phosphorylation can be impaired as well. Therefore, ALDH7A1-mediated NADH may play dual role in the regulation of glycolysis and oxidative phosphorylation. To evaluate the two main metabolic pathways, mitochondrial respiration and glycolysis, in heated Huh-7 cells after various treatments, we measured glucose uptake, ATP level, lactate production, OCR, and ECAR, in addition to cellular malignancy phenotypes and their markers. After we initially found that Tan IIA could reverse the enhanced cell viability and invasion and induce cell cycle arrest at the G2/M phase and apoptosis at both the cellular and molecular levels (Figs. 1 and 2), we consistently demonstrated that the increases in glucose uptake, ATP production and ECAR were reversed, and the basal respiration was significantly increased by Tan IIA, in heat-treated Huh-7 cells (Fig. 4). Through manipulation of ALDH7A1 expression using lentivirus, we confirmed that the knockdown and overexpression of ALDH7A1 ran in the opposite direction, resulting in inhibited glycolysis and promoted oxidative phosphorylation, respectively (Figs. 5 and 6). We noticed the slight inhibition of lactate production caused by Tan IIA, and deemed that this may result from its other potential targets that can influence glycolysis directly or indirectly, or perhaps simply the lower drug concentration [34, 35]. Based on these findings, we conclude that ALDH7A1 has a greater effect on oxidative phosphorylation than on glycolysis by generating NADH. Additionally, Tan IIA likely upregulates oxidative phosphorylation relatively directly and inhibits glycolysis indirectly, at least in part, by targeting ALDH7A1. Furthermore, the increase in ROS observed after Tan IIA treatment in this study may be partly contributed by this regulation.

Because of the known osmoregulative effects of ALDH7A1, we also investigated its enzymatic and osmolytic products, betaine and d-glycerate. As a result, both osmolytes were significantly reduced by Tan IIA treatment or by knocking down ALDH7A1 in heated Huh-7 cells. In contrast, ALDH7A1 overexpression remarkably increased them. Betaine plays a crucial role in regulating hydration within hepatocytes and safeguards the liver against various types of stress. The availability of betaine is controlled to adapt to changes in cell volume, and CHDH and CHK facilitate the initial stage of betaine production [36]. By looking into the gene expression data again from RNA-seq (data not shown), we noticed that, the mRNA level of CHDH was decreased but CHK was not changed in heat-induced Huh-7 cells, and after the heated cells were treated with Tan IIA, the two genes’ expression was not changed either, suggesting the presence of other pathways regulating betaine, such as those mediated by ALDH7A1. d-glycerate can be derived from d-glyceraldehyde under the catalysis of ALDH7A1, and it can enter the glycolysis pathway as an important metabolic intermediate and simultaneously serve as the precursor of osmolyte glucosylglycerate [17, 37]. In heated Huh-7 cells, but not in heated Hep-G2 cells, ALDH7A1 expression was significantly activated at the transcriptional level. However, Tan IIA can effectively inhibit its activity, directly abolishing the production of betaine and d-glycerate, leading to the vulnerability of Huh-7 cells to hyperosmotic stress (Fig. 4). Similarly, in low-ALDH7A Huh-7 cells after heat induction, the lower levels of betaine and d-glycerate are possibly one of the reasons that NaCl- or glucose-mediated hyperosmotic stress causes increased apoptosis and reduced invasion (Fig. 5).

It is well-known that cancer cells usually have higher basal ROS level than normal cells, which play a dual role in cell metabolism [38], and heat induces ROS in various cancers for a long time. However, the underlying mechanism of the tumor protective role of ROS in HCC cells induced by sublethal heat was recently reported by Peng et al. [39]. They pointed out that the ROS burst following incomplete RFA, driven by NOX4 and leading to increased mitochondrial ROS production, exerts a pro-survival effect through PINK1-dependent mitophagy and elevated Nrf2 expression to remove damaged mitochondria [39]. In our study, we also found a significant increase in ROS after heat induction. When Tan IIA was applied to Huh-7 cells, either with or without heat pretreatment, ROS levels also increased (Figs. 4W and S4A). However, their staining morphologies differed, which may indicate different types of ROS—mitochondrial ROS and cytosolic ROS, respectively (Fig. 4W and S4A). Mitochondrial ROS are a major driver of lipid peroxidation within the mitochondria, damaging mitochondrial membranes and potentially leading to mitochondrial dysfunction and cell injury. Cytosolic ROS originates from various sources like NADPH oxidases and mitochondrial electron-transport chain, and it also triggers lipid peroxidation that can affect cellular integrity and signaling pathways [40]. We did not find any study reporting distinct functions for ROS distributed in different positions, but a report claims that cytosolic ROS stimulate ferroptosis in conjunction with ferric ammonium citrate-induced mitochondrial LPO [41]. This finding suggests that cytosolic ROS and mitochondrial ROS probably synergistically regulates downstream pathways and biological processes. In our study, we further showed that shALDH7A1 slightly decreased ROS, and ALDH7A OE strongly increased both the ROS level and the proportion of ROS-positive cells in heated Huh-7 cells under isosmotic conditions (Figs. 5T, 6J and S4B). Therefore, we speculate that Tan IIA causes excessively high and lethal ROS in some cells through mechanisms other than merely targeting ALDH7A1, such as targeting PERK [42] or forming semiquinone radical (hydroquinone-TIIA¯·) by itself via reduction mediated by NAD(P)H quinone dehydrogenase 1 (NQO1). In addition, ALDH7A1 may not be crucial for maintaining the basal ROS level in Huh-7 cells, but its overexpression will definitely increase the basal ROS level of most cells. However, in hyperosmotic culture media, ALDH7A-OE cells did not show higher ROS levels than EV-infected cells (Figs. 7C and S4C). This might be caused by the higher ROS induced by hyperosmotic conditions overlapping the relatively slight enhancement by ALDH7A1 overexpression. Simultaneously, MDA was reduced, and the activities of two antioxidant enzymes SOD and GSH-Px were increased by heat or Tan IIA, which is consistent with several previous studies [43, 44], but is discrepant from the results of ROS and seems hard to understand. The reduced MDA is a typical result of increased activities of SOD and GSH-Px, but the precise mechanism by which Tan IIA upregulates SOD and GSH-Px has not yet been reported, to our knowledge. In our study, we noticed that MDA was not significantly different after manipulation of ALDH7A1 expression. Therefore, we believe distinct molecular mechanisms underlie how Tan IIA regulates the antioxidant enzymes and ROS, warranting further exploration.

To demonstrate the mediating role of ALDH7A1 in Tan IIA’s antitumor effect, we used Tan IIA to treat the Huh-7 with low levels of ALDH7A1, or overexpressed ALDH7A1 in the Huh-7 cells treated with Tan IIA in the presence of hyperosmotic stress. As a result, deficiency of ALDH7A1 led to the resistance of Huh-7 cells to Tan IIA treatment, and ALDH7A1 OE rescued the apoptosis and ROS induced by Tan IIA, restored the levels of lactate and osmolytes in hyperosmotic culture media (Figs. 7 and S4C). In summary, we conclude that Tan IIA inhibits p53 mutant Huh-7 cells at least partly by targeting ALDH7A1-mediated homeostasis of glycolysis, osmosis, and ROS. Hyperosmotic stress impacts migration of several types of cancer cells [45], and it induces apoptosis by triggering various molecular pathways, including ROS generation, in a cell type-specific fashion [46]. Therefore, the dysregulation of cellular osmotic pressure mediated by ALDH7A deficiency is a vital link influencing ROS, cell invasion, and apoptosis in some specific cancer cells.

Our in vivo experiments further support the therapeutic potential of combining Tan IIA with hyperthermia and hyperosmotic stress. The addition of hyperosmotic agents like NaCl to Tan IIA treatment significantly enhanced tumor suppression in heat-induced HCC models. This combination therapy appears to exert its effects by further destabilizing the metabolic and oxidative balance within tumor cells, leading to reduced tumor growth and increased apoptosis. The promising strategy that combining NaCl-mediated hyperosmotic stress with some anticancer drugs like Tan IIA is also an easily accessible and feasible way to prevent and treat HCC after thermal ablation. However, in the future, there are still many aspects to explore. First, the mechanism underlying heat induced tumor growth is far from being revealed. The influence of temperature on the immune response in the microenvironment deserves more attention [47]. A less enriched immune-related KEGG pathway TNF signaling pathway in our study supports this notion (Fig. 3A). In addition, it’s worth noting that pathways in cells carrying different mutations (e.g., TP53) vary distinctly after heat induction. Second, Tan IIA has many other targets, whose changes may not be reflected by their transcriptional levels. As aforesaid, it also targets PERK [42]. In our experiments, we observed that Tan IIA is also predicted to target the protein encoded by SUCLG1, which is the α subunit of succinate-CoA ligase that plays important roles in the citric acid cycle [48]. Third, DEAB is a newly proven specific inhibitor of ALDH7A1, but its effective concentration or other potential targets should be investigated before its specific application. Ultimately, the role of ALDH7A1 in glycolysis, osmoregulation, and ROS still needs more evidence, and other downstream signal pathways are also worth exploring further, which will lay a solid foundation for us to use it as a potential target in HCC treatment. Lastly, the safety, tolerability, potential systemic effects, and mechanism of the therapy involving the combination of highly concentrated saline and RFA also need further investigation. Although high-concentration NaCl has been used experimentally and clinically in RFA, and despite observations suggesting improved efficacy in our clinical practice (unpublished) as well as in other studies [49–51], and the safety of the concentrated saline is reported to depend on both the concentration and the volume, for instance, an infusion of 15% NaCl as a bolus into the tumor will likely not have any major impact to the body [52], more experimental validation and clinical trial on the safety of this combination should be conducted before its extensive use. Additionally, we believe the improved efficacy results from increased tissue electrical conductivity due to a higher number of ions, greater energy deposition, and enlarged coagulation volume [49, 50, 53], as well as osmotic balance dysregulation. Therefore, more research into osmotic regulation systems is necessary to develop safer and more effective drugs for cancer RFA therapy.

## Conclusions

**Fig. 9:**
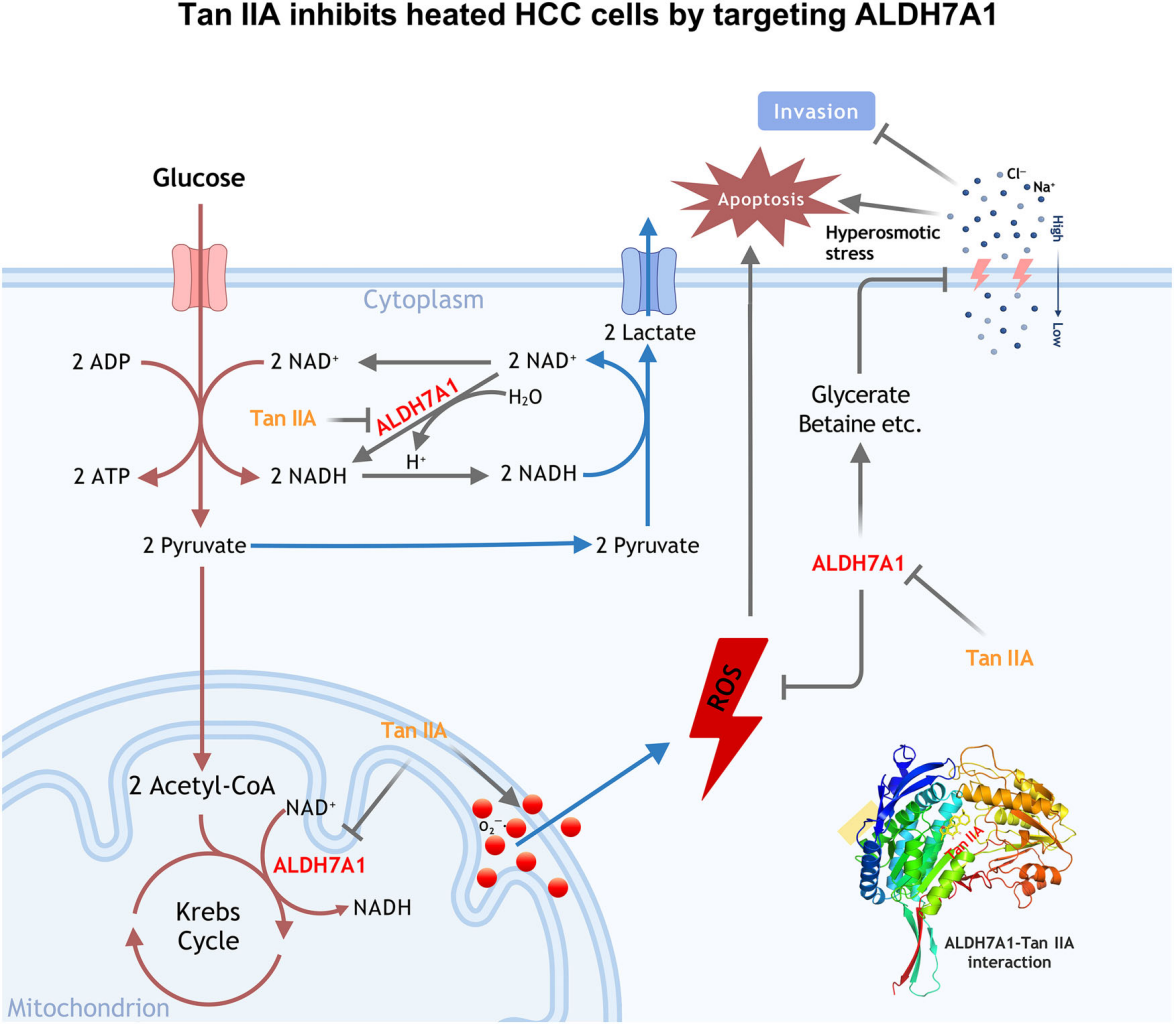
Schematic diagram of ALDH7A1’s role in regulating glycolysis and osmosis in heated HCC cells.

In conclusion, hyperthermia therapy, such as RFA, often leads to rapid recurrence if any tumor cells survive. As summarized in Supplementary Table S3 and depicted in Fig. 9, in p53-mutant Huh-7 cells, Tan IIA shows a stable anti-tumor effect, particularly under heat stress conditions, by targeting the protein ALDH7A1. ALDH7A1 plays a crucial role in protecting the HCC cells from heat stress. It is involved in the production of betaine and d-glycerate, which help cells adapt to hyperosmotic conditions, and NADH, which acts as an electron carrier and participates in cellular energy metabolism. Additionally, ALDH7A1 has antioxidant properties, neutralizing harmful reactive oxygen species (ROS) and thereby enhancing cell survival under heat or hyperosmotic stress. Tan IIA’s anti-invasion and proapoptotic effect on Huh-7 cells is at least partly due to its inhibition of ALDH7A1, which disrupts these protective mechanisms. By targeting ALDH7A1, Tan IIA disturbs the cellular osmotic balance and increases the vulnerability of HCC cells to hyperthermia, reducing the chances of recurrence. In summary, Tan IIA enhances the effectiveness of hyperthermia in treating HCC by inhibiting ALDH7A1, thereby weakening the cancer cells’ defenses against heat-induced stress. Our findings underscore the potential of Tan IIA as a powerful anti-cancer agent, particularly when used in conjunction with hyperthermia and/or hyperosmotic stress. To prevent or treat recurrence of hepatocellular cancer following thermal ablation, drug or drug combinations targeting glycolytic or osmotic homeostasis might need extra attention. However, the complex interplay between hyperthermia, tumor biology, and therapeutic response warrants further investigation to optimize treatment protocols and improve clinical outcomes.

## Methods

### Cell culture and reagents

HCC cell lines Huh-7 (FH0075) and Hep-G2 (FH0076) were obtained from Shanghai Fuheng Biotechnology Co., Ltd., China. The cell lines were recently authenticated by STR profiling and excluded mycoplasma contamination. Cells were cultured in Dulbecco’s Modified Eagle Medium (DMEM; C3113-0500M, VivaCell, China) supplemented with 10% fetal bovine serum (FBS; 10100147, Gibco, USA), 1% penicillin-streptomycin (PB180120, Procell, China) at 37 °C in a humidified incubator with 5% CO_2_.

### Cell treatment

#### Heat induction

Heat induction was performed as previously described [54]. The water bath (HH Digital Constant Temperature Water Bath, LiChen, China) was heated to the required temperature in advance (the first gradient setting included 37, 45, 50, and 55 °C; the second gradient setting included 37, 42, 43, and 44 °C). The culture plate was gently shaken to ensure that the cells were heated evenly, and then placed in the water bath for 10 min of heat stimulation. Afterward, the cells were immediately transferred back to standard culture conditions, washed to remove non-adherent cells, and then reseeded at 5000 cells per well in a new 96-well plate with DMEM. The cells were continued to be cultured at 37 °C for 24, 48, or 72 h.

#### Drug treatment

3-BrPA (3-bromopyruvate, HY-19992, MCE, USA), As_2_O_3_ (1327-53-3, Sigma-Aldrich, USA), and Tan IIA (T109795, Aladdin, China) were dissolved in dimethyl sulfoxide (DMSO). The Huh-7 or Hep-G2 cells were digested using 0.25% pancreatic enzyme (C3530-0500, VivaCell) and then inoculated with 5000 cells per well into 96-well plates for culture. After cell adhesion, cells were treated with different concentrations of drugs (3-BrPA: 20, 30, 40, and 80 μM; As_2_O_3_: 3, 6, and 12 μM; Tan IIA: 2, 4, 8, and 16 μM) for 24, 48, and 72 h. Control cells were treated with DMSO.

#### Hypertonic treatment

The cells were thermally stimulated at 44 °C for 10 min and then transferred back to standard culture conditions for normal culture. The following day, 120 mM NaCl or 250 mM D-glucose (ST1227s Beyotime Biotechnology, China) was added and incubated at 37 °C for 48 h.

### Construction of stable lentiviral transductants of ALDH7A1

The interfering sequences targeting the conserved region of ALDH7A1 were inserted into lentiviral silencing plasmid JS039 (U6-MCS-CMV-ZsGreen-PGK-Puro), and the CDS region of ALDH7A1 was cloned into the lentiviral overexpression plasmid JS037 (CMV-MCS-3flag-EF1-ZsGreen-T2A-puro), using the BamHI and EcoRI restriction enzymes (R0136/R0101, NEB). Sequences were synthesized at GenePharma (China). ShALDH7A1-1: 5′-GATCCGGGAGAAGATCCAAGTACTACTCGAGTAGTACTTGGATCTTCTCCCGTTTTTG-3′ (S), 5′-AATTCAAAAACGGGAGAAGATCCAAGTACTACTCGAGTAGTACTTGGATCTTCTCCCG-3′ (A); ShALDH7A1-2: 5′-GATCGCTCCGATTCTCTATGTCTTTCTCGAGAAAGACATAGAGAATCGGAGCTTTTTG-3′ (S), 5′-AATTCAAAAAGCTCCGATTCTCTATGTCTTTCTCGAGAAAGACATAGAGAATCGGAGC-3′ (A); ShALDH7A1-3: 5′-GATCGCGAGGCGACTGTTTATACATCTCGAGATGTATAAACAGTCGCCTCGCTTTTTG-3′ (S), 5′-AATTCAAAAAGCGAGGCGACTGTTTATACATCTCGAGATGTATAAACAGTCGCCTCGC-3′ (A). The recombinant plasmid was mixed with the packaging vector and shuttle vector, and then incubated in the transfection reagent at room temperature for 25 min. The transfection mixture infected 293T cells in a serum-free medium for 4 h with polycoaguline (H8761, Solarbio, China). The harvested viral supernatant, containing the lentivirus particles, was used to infect Huh7 cells at a MOI of 100. The cells were further selected with 2 μg/ml Puromycin (SX50113, Shanghai Shaoxinbio Co., Ltd., China).

### Sample size chosen and inclusion/exclusion criteria

The sample sizes for both cell (*n* = 3) and animal (*n* = 5 per group) experiments were chosen based on common practice and prior published studies in the field of cell and molecular biology. No formal power calculation was performed prior to the experiments. The selected sample sizes are considered generally adequate to detect biologically meaningful changes under controlled experimental settings, while also considering resource constraints and ethical aspects.

Inclusion and exclusion criteria were pre-defined at the beginning of the study. In this study, no samples/animals met the exclusion criteria, and, therefore, all samples were included in the final analysis.

### Cell viability assay

The Cell Counting Kit-8 (CCK-8; C0039, Beyotime) was used to analyze the viability of HCC cells after treatment. Briefly, CCK-8 reagent was added to each well and gently mixed, followed by further incubation in the 37 °C for 1 h. Wells containing the corresponding amounts of cell culture medium and CCK-8 solution but without cells were set up as blank controls. The absorbance at 450 nm was measured using Thermo Scientific™ MULTISKAN GO microplate reader (1510, USA).

### RNA extraction and quantitative real-time PCR (RT-qPCR)

The Huh-7 cells or Hep-G2 cells were seeded in 24-well plates and lysed with Trizol reagent (15596026, Invitrigen, USA). Total RNA was purified from the lysate using the phenol-chloroform (TriReagent) method. According to the manufacturer’s instructions, the synthesis of cDNA was performed using the HiScript III All-in-one RT SuperMix (R333-01, Vazyme, Denmark), and the resulting cDNA was then used as a template for qRT-PCR on the qTOWER3G Real-Time PCR Thermal Cycler from Analytikjena (Germany) with SYBR Green Master Mix (Q711-02, Vazyme). The relative expression levels of genes were normalized to GAPDH and calculated using the 2^−∆∆Ct^ method. Primers used are listed in Supplementary Table S1.

### Western blotting

Protein extraction was performed using the lysis buffer for Western (P0013, Beyotime) supplemented with PMSF (P0013, Beyotime). Protein concentrations were determined with a BCA Protein Assay Kit (P0010, Beyotime). Equal amounts of protein were separated by SDS-PAGE and transferred to NC membranes (0.22 µm, 66485, Pall, China). Membranes were blocked with 5% nonfat dry milk in TBST (8.8 g Nacl, 2.4 g Tris Base, 1% Tween-20) and incubated with primary antibodies: anti-MMP2 (ab181286, Abcam, Dilution ratio: 1:1000), anti-MMP9 (GB12132-1, Servicebio; dilution ratio: 1:500), anti-N-Cadherin (22018-1-AP, Proteintech; dilution ratio: 1:8000), anti-Bcl-2 (AF6139, Affinity Biosciences; dilution ratio: 1:1000), anti-Bax (50599-2-Ig, Proteintech; dilution ratio: 1:8000), anti-cleaved Caspase 3 (AF7022, Affinity; dilution ratio: 1:1000), anti-cyclin D1 (AF0931, Affinity; dilution ratio: 1:1000), anti-CDK1 (19532-1-AP, Proteintech; dilution ratio: 1:5000), anti-MAOB (Cell Signaling Technology, Beverly, MA, USA), anti-DAO (13273-1-AP, Proteintech; dilution ratio: 1:1000), anti-ALDH7A1 (10368-1-AP, Proteintech; dilution ratio: 1:2000) overnight at 4 °C. Secondary antibodies conjugated to HRP SA00001-2, Proteintech) were applied at room temperature for 1 h. GAPDH (AB0037, Abways; dilution ratio: 1:5000) and anti-Histone H3 (AF0863, Affinity Biosciences; dilution ratio: 1:1000) function as internal controls for protein standardization. Signals were visualized using ECL reagents (P0018S, Beyotime) and detected with the Tanon-4600 imaging system (China).

### Cell invasion assay

Transwell chambers (Corning, USA) coated with Matrigel (354277, Corning) were used to assess cell invasion. The Matrigel was melted overnight at 4 °C and then diluted with a serum-free medium pre-cooled at 4 °C to a final concentration of 1 mg/mL. This mixture was then added to the transwell upper chamber and incubated at 37 °C for 4 h. HCC cells were resuspended in serum-free medium. Approximately 1 × 10^5^ cells were seeded in the upper chamber in serum-free DMEM, while the lower chamber contained DMEM with 10% FBS. After a 24-h incubation, cells on the upper surface of the membrane were removed, and the invaded cells on the lower surface were fixed with 4% paraformaldehyde (G1101, Servicebio) and stained with 0.1% crystal violet. Invaded cells were counted in five random fields under a microscope.

### Cell apoptosis assay

The apoptosis of HCC cells after treatment was evaluated using an Annexin V-FITC/PI Apoptosis Detection Kit (40302ES60, Yeasen, China) and an Annexin V-APC/7-AAD Apoptosis Detection Kit (P-CA-208, Procell). Briefly, cells were digested with EDTA-free pancreatic enzymes and subsequently resuspended in a 1×Bingding Buffer. Next, Annexin-V-FITC/Annexin V-APC and PI/7-AAD staining solution was added to the cell suspension for double staining, gently swirled and mixed, and incubated at room temperature for 15 min. Flow cytometry analysis was performed using a FACSCanto II flow cytometer (BD Biosciences) and analyzed with CellQuest software (BD Biosciences).

### Cell cycle analysis

The Cell cycle was evaluated using a Cell Cycle and Apoptosis Analysis Kit (C1052, Beyotime). Briefly, cells were collected and fixed in pre-cooled 70% ethanol at 4 °C overnight. The next day, the mixture was centrifuged at 1000 × *g* for 5 min to remove the ethanol, and the cells were then resuspended in the Buffer. PI staining solution supplemented with RNase A was added to stain the DNA at 37 °C for 30 min. The flow cytometry was detected at an excitation wavelength of 488 nm and analyzed with CellQuest software.

### RNA sequencing and differential gene expression analysis

Total RNA was extracted from Huh-7 and Hep-G2 cells using the TRIzol reagent (Invitrogen). The cells were treated under various conditions as described: a blank control; exposure to 8 μM Tan IIA for 48 h; treatment with 8 μg/mL 4-diethylaminobenzaldehyde (DEAB) for 48 h; shALDH7A1 infection for 48 h; preheating at 44 °C (Huh-7) or 45 °C (Hep-G2) for 10 min; and 8 μM Tan IIA treatment for 48 h following preheating at 44 °C for 10 min. The RNA integrity and concentration were evaluated using an Agilent 2100 Bioanalyzer (Agilent Technologies, Santa Clara, CA, USA). RNA libraries were constructed using the TruSeq RNA Library Prep Kit (Illumina, San Diego, CA, USA) and sequenced on an Illumina HiSeq 4000 platform to generate 150 bp paired-end reads. Raw sequencing data were processed and analyzed following standard protocols. Reads were aligned to the human reference genome (GRCh38) using STAR aligner, and gene expression levels were quantified using HTSeq. Differentially expressed genes (DEGs) were identified using the DESeq2 package in R, with an adjusted *p* value < 0.05 and a fold change > 2 as thresholds. The raw data and processed data of RNA sequencing results have been deposited in GEO database with an accession number of GSE309428.

### Enrichment analysis

Gene Ontology (GO) enrichment analysis of DEGs was conducted using the DAVID Bioinformatics Resources 6.8 (https://david.ncifcrf.gov/). DEGs were categorized into three ontological categories: Biological Process (BP), Molecular Function (MF), and Cellular Component (CC). Enrichment scores were calculated based on the hypergeometric distribution, and terms with a *p* value < 0.05 were considered significantly enriched.

Kyoto Encyclopedia of Genes and Genomes (KEGG) pathway enrichment analysis was performed using the KEGG database (http://www.genome.jp/kegg/). DEGs were mapped to the KEGG pathways, and enrichment analysis was conducted using the KOBAS tool (2.0). Pathways with a *p* value < 0.05 were considered significantly enriched.

Gene Set Enrichment Analysis (GSEA) was performed using GSEA software (Broad Institute, Cambridge, MA, USA). Pre-ranked gene lists based on their fold change values were used for the analysis. The hallmark gene sets (h.all.v7.1) from the Molecular Signatures Database (MSigDB) were utilized to identify significantly enriched biological pathways. The number of permutations was set to 1000, and pathways with a false discovery rate (FDR) < 0.25 and nominal *p* value < 0.05 were regarded as significantly enriched.

### Measurement of glycolytic activity

#### Glucose uptake

Glucose uptake was measured using 2-deoxyglucose (2-DG) (ab136955, Abcam), a glucose derivative that can be metabolized as 2-DG-6-phosphate (2-DG6P) to accumulate in the cell. Cells were starved 2 h before the experiment and incubated in a medium without FBS. Afterward, 2-DG was added and incubated cells at 37 °C for 20 min. Next, a lysis was performed and the lysate was heated at 85 °C for 40 min. The lysate was treated with Enzyme Mix III at 37 °C for 1 h, followed by incubation at 90 °C for 40 min. Finally, Glutathione Reductase and DTNB were added. The output was measured at OD 412 nm using a microplate reader in kinetic mode, at 37 °C, every 2–3 min.

#### L-lactate A

L-Lactate Assay Kit (ab136955, Abcam) was used to quantify lactate production. Briefly, cells were collected and prepared into a homogenate solution. Next, 2 μl of OxiRed Probe was added and incubated at room temperature for 30 min away from light. The absorbance at OD 570 nm was measured on a microplate reader.

#### Adenosine triphosphate (ATP)

An Enhanced ATP Assay Kit (S0027, Beyotime) was used to assess ATP levels. Briefly, cells were collected and prepared for extraction into a cell lysate. ATP assay reagent was added, and RLU values were measured by luminometer.

### Mitochondrial stress and Glycolysis stress tests

Cellular energy metabolism was evaluated using the Agilent Seahorse XFe96 Analyzer (model XFe96). Glycolytic and mitochondrial stress tests were performed with the Seahorse XF Cell Glycolysis Stress Test Kit (Agilent, #103020-100) and Seahorse XF Cell Mito Stress Test Kit (Agilent, #103015-100), respectively. Cells were cultured in a Thermo Forma 3111 incubator. Mitochondrial respiration was evaluated following sequential injections of oligomycin, FCCP, and Rot-AA. For glycolysis assessment, cells were sequentially treated with glucose, oligomycin, and 2-DG. Probe plates were hydrated overnight with sterile water, calibrated in XF buffer (37 °C, O₂-free), and cells were washed with assay medium (DMEM, GlutaMAX, pyruvate) prior to drug injection. Protocols adhered to Agilent Seahorse XF kit guidelines.

### Measurement of ALDH7A1 metabolites-related indexes

#### NADH

A NAD^+^/NADH Assay Kit (S0175, Beyotime) was used to the levels of NADH within cells by quantifying the reduction reaction of WST-8. Briefly, cells were collected and lysed with NAD^+^/NADH extract. Subsequently, the lysate was heated in a water bath at 60 °C for 30 min, and 30 μl of ethanol dehydrogenase reagent was added and incubated at 37 °C for 10 min. The absorbance at OD 450 nm was then measured.

#### Betaine

A Betaine Content Assay Kit (BC3135, Solarbio) was used to betaine levels. Cells were prepared in accordance with the manufacturer’s instructions. The absorbance at OD 525 nm was then measured.

#### D-glycerate

A series of successive enzymatic reactions was carried out to quantify D-glycerate. Lactate dehydrogenase (D649636, Aladdin), D-Glycerate kinase (EXWM-2995, Creative Enzymes), ATP (A265857, Aladdin), enolase (NATE-0941, Creative Enzymes), pyruvate kinase (NATE-0938, Creative Enzymes), and reduced diphosphopyridine nucleotide (DPNH; L433357, Aladdin) were added to the cell lysate successively for enzymatic reaction. The results were analyzed by spectrophotometry.

### Measurement of oxidative stress and lipid peroxidation

#### Reactive oxygen species (ROS)

ROS production was measured using Dihydroethidium (S0063, Beyotime). Dihydroethidium was dissolved in DMSO. Fluorescent probe loading was performed by incubating cells with 2 µM of dihydroethidium at 37 °C for 30 min. Following washing, flow cytometry and fluorescence microscope were used for detection.

#### Malondialdehyde (MDA)

Lipid peroxidation was assessed by measuring malondialdehyde (MDA) levels using an MDA assay kit (S0131S, Beyotime). Briefly, cells were collected, lysed, and concentrations were determined. Subsequently, the lysate was mixed with MDA assay reagent and heated at 100 °C for 15 min. The absorbance at OD 450 nm was then measured.

#### SOD and GSH-Px

Two classic antioxidant enzymes, SOD and GSH-Px, in Huh-7 cells induced at 37 °C or 44 °C were measured using the following assay kits: GSH-Px (Nanjing Jiancheng, A005-1-1), and SOD (Beyotime, S0101S), according to the manufacturer’s instructions.

### Cellular thermal shift assay (CETSA)

The cells were thermally stimulated at 44 °C for 10 min and then transferred back to standard culture conditions for normal culture. The following day, the cells were treated with Tan IIA (0, 4, 8 µM) or DEAB (0, 4, 8 µg/mL; D104213, Aladdin) for 24 h. Total protein was then extracted, and the protein samples were placed in a PCR instrument at 63 °C for 5 min. Changes in the abundance of ALDH7A1 were detected by Western Blotting.

### In vivo tumorigenesis

All animal experiments that complied with the ARRIVE (Animal Research: Reporting of In Vivo Experiments) guidelines were conducted in accordance with ethical guidelines and approved by the Ethics Committee of the First Affiliated Hospital of Zhengzhou University (Approval number: ZZU-LAC20220427[01]). Female BALB/c nude mice (nu/nu), aged 4–5 weeks, were purchased from Shanghai Sippr-bk Laboratory Animal Co., Ltd (China) for the establishment of xenograft tumor models. The mice were kept in a 12-h light/dark cycle, a temperature range of 21 ± 2 °C, and a relative humidity of 40–60%, with free access to water and food. Using a random number table, a total of thirty mice were randomly divided into six groups: non-heated group (*N* = 5), heated group (*N* = 5, 44 °C), heated+Tan IIA group (*N* = 5, 44 °C), heated+Tan IIA+NaCl group (*N* = 5; 44 °C, 40 mg/kg TanIIA, 120 mM Nacl), heated+Tan IIA + 2-DG group (*N* = 5, 44 °C; 40 mg/kg TanIIA, 1000 mg/kg 2-DG), and heated+cisplatin group (*N* = 5, 44 °C, 4 mg/kg cisplatin). Huh-7 cells were treated with either non-heat shock or heat shock for 10 min. The Huh-7 cells in the logarithmic growth phase (8 × 10^6^) were injected beneath the skin of the mice. After the tumor grew to 100 mm^3^, drugs were injected into the tumor mass according to the group and treated continuously for 21 days. The length and width of the tumor were measured with a vernier caliper (Meinaite) every week, and the tumor volume was calculated using the formula (length × width^2^)/2. The investigators were blinded to group allocation during the conduct of the experiment and outcome assessment. A tumor growth curve was then plotted based on these measurements. The mice were euthanized and photographed. The tumor samples were collected, partially fixed, and partially frozen at −80 °C. Following fixation, the cancer tissue underwent a series of steps, including alcohol gradient dehydration (Sinopharm, China), xylene clearing (Sinopharm), and wax immersion for paraffin embedding in preparation. Using a rotary microtome (Leica RM2016), cancer tissue was cut into 5 µm slices for histological analysis.

### Immunohistochemistry

The slices were first dewaxed and then placed in a citric acid (pH 6.0/pH 9.0) antigen repair solution (G1202/G1203, Servicebio) in a pressure cooker for 3 min to repair antigens. Immunohistochemical pens were used to draw hydrophobic boundaries around cells. Slices were treated with 3% H_2_O_2_ solution (10011218, Sinopharm) for 25 min to inactivate endogenous peroxidase. Next, slices were covered with 3% BSA (G5001, Servicebio) and blocked at room temperature for 30 min. The diluted primary antibody (ALDH7A1: 1:500; Ki67: 1:300; E-Cadherin: 1:2000; N-Cadherin: 1:2000) was added and incubated in a wet box at 4 °C overnight. The HRP-conjugated goat anti-rabbit antibody (GB23303, Servicebio, 1:200) was incubated with the samples at room temperature for 1 h. Color development was performed using a DAB substrate (K5007, DAKO), followed by Hematoxylin restaining (G1004, Servicebio) of the nucleus for 3 min. The sections were sealed with a neutral mounting medium (G1403, Servicebio), and the images were then observed and captured under an optical microscope.

### TUNEL assay

Apoptosis in tumor tissue sections was assessed using a TUNEL assay kit (11684817910, Roche) following the manufacturer’s instructions. Briefly, tumor sections were dewaxed and rehydrated, followed by treatment with proteinase K for permeabilization (G1205, Servicebio). The tissue was then covered with the membrane breaking solution (G1204, Servicebio) and incubated at room temperature for 20 min. The TUNEL reaction mixture was prepared and incubated with slices at 37 °C for 2 h. DAPI restained nucleus for 10 min. The sections were sealed with an anti-fluorescence quenching (G1401, Servicebio), and the images were then observed and captured under an optical microscope.

### Statistical analysis

Data are expressed as mean ± standard deviation (SD) from at least three independent experiments. Statistical analyses were conducted by investigators blinded to group allocation. The normality of data distribution was first assessed using the Shapiro–Wilk test. For comparisons between two groups, an independent samples *t*-test was used if the data were normally distributed and passed the Levene’s test for homogeneity of variances. If the data did not meet these assumptions, the non-parametric Mann–Whitney U test was used instead. For normally distributed data, one-way or two-way ANOVA was used to assess overall differences, followed by Tukey’s post hoc test for precise identification of significant group differences while controlling for multiple comparisons. For non-normally distributed data, the Kruskal–Wallis test followed by Dunn’s post-hoc test was used. *P* values < 0.05 were considered statistically significant. Analyses were performed using GraphPad Prism 8.0.1 software (GraphPad Software, La Jolla, CA, USA).

## Supplementary information

Supplementary figure legends and supplementary table captions

Figure S1

Figure S2

Figure S3

Figure S4

Figure S5

Supplementary Table S1

Supplementary Table S2

Supplementary Table S3

Original WB images

## Data Availability

The data that support the findings of this study are available from the corresponding author upon reasonable request.
