# Supplementary material for: Tanshinone IIA inhibits heat-induced growth of p53-mutant Huh-7 hepatocellular carcinoma by modulating osmotic homeostasis and glycolysis through targeting ALDH7A1
Source: Cell Death Discov. 2025 Oct 31;11:493. doi: 10.1038/s41420-025-02795-0 (PMC12579247; doi:10.1038/s41420-025-02795-0)
Supplement: Supplementary file 1 — Supplementary figure legends and supplementary table captions [file 41420_2025_2795_MOESM1_ESM.doc]

**Supplementary figure legends and supplementary table captions**

**Supplementary Fig. S1**. **The influences of high temperature and/or various antitumor drugs on HCC cells.** **A-B**. The viability of cells pretreated at various temperatures (37, 45, 50 and 55 °C) measured using CCK-8 assay. * *p* < 0.05, ** *p* < 0.01, compared with 37 °C at the same time point. **C-D**. The influences of 3 antitumor drugs on viability of HCC cells pretreated with a high temperature of 45 °C (floating cells after heat induction were not removed before reinoculation, and the viability were measured after reculture for 24 h). * *p* < 0.05, ** *p* < 0.01, compared with NC. **E-F**. The expression of proliferation biomarkers (PCNA and KI67) at the transcription level measured using qPCR after treatment with Tan IIA for 24 on the HCC cells that were pretreated with high temperatures of 37, 42, 43 and 44 °C. * *p* < 0.05, ** *p* < 0.01, compared with NC at the same temperature; NS: not significant.

**Supplementary Fig. S2**. **The expression profile of all DEGs in Huh-7 cells among 4 groups as indicated (A) and KEGG pathway enrichment result (B) of DEGs between NC and Heat groups.** White outlined boxes indicate the genes that were induced by heat but then reversed by Tan IIA treatment. Blue line boxes indicate the genes that were significantly altered by Tan IIA in heat-induced cells.

**Supplementary Fig. S3. The effects of Tan IIA on gene expression in Hep-G2 cells. A-C**. KEGG pathway enrichment of DEGs in Hep-G2 cells before and after treatment with Tan IIA, which were pretreated with (**A, C**) or without heat (**B**). **D**. GSEA results of three key pathways, Cell cycle, Complement and coagulation cascades, and p53 signaling pathway. **E**. GO enrichment of DEGs between Heat vs Heat + Tan IIA groups.

**Supplementary Fig. S4**. **Representative images of reactive oxygen species (ROS) stained with** **dihydroethidium (DHE) probes. A**. ROS in Huh-7 cells treated with or without Tan IIA after induction at 44 °C. **B**. ROS of Huh-7 cells after ALDH7A1 knockdown or overexpression. **C**. ROS of Huh-7 cells overexpressing ALDH7A1 treated with Tan IIA or DEAB in the absence or presence of hyperosmotic stress.

**Supplementary Fig. S5**. **Bioinformatic analysis results of heat-induced Huh-7 cells after various treatments.** **A**. Cluster heat map of DEGs among groups, which were treated with shALDH7A1, DEAB, or Tan IIA. **B-C**. The Venn diagrams indicating the number of the mutually upregulated or downregulated genes by Tan IIA, shALDH7A1 or DEAB. **D-F**. GO enrichment results among 3 groups. Blue dotted boxes indicate the overlapped GO terms between 2 or among 3 comparisons (Blank vs shALDH7A1, Blank vs Tan IIA, and Blank vs DEAB). **G-I**. KEGG pathway enrichment results of DEGs among groups. Blue dotted box indicates the overlapped KEGG pathway terms between 2 or among 3 comparisons.

**Supplementary Table S1. Primers used in this study.**

**Supplementary Table S2. DEGs before and after ALDH7A knockdown with various methods and the results of their GO and KEGG pathway enrichment analyses.**

**Supplementary Table S3. Main results of various treatments on Huh-7 cells with or without heat induction.**
