# Supplementary figures and images for: Tanshinone IIA inhibits heat-induced growth of p53-mutant Huh-7 hepatocellular carcinoma by modulating osmotic homeostasis and glycolysis through targeting ALDH7A1
Source: Cell Death Discov. 2025 Oct 31;11:493. doi: 10.1038/s41420-025-02795-0 (PMC12579247; doi:10.1038/s41420-025-02795-0)

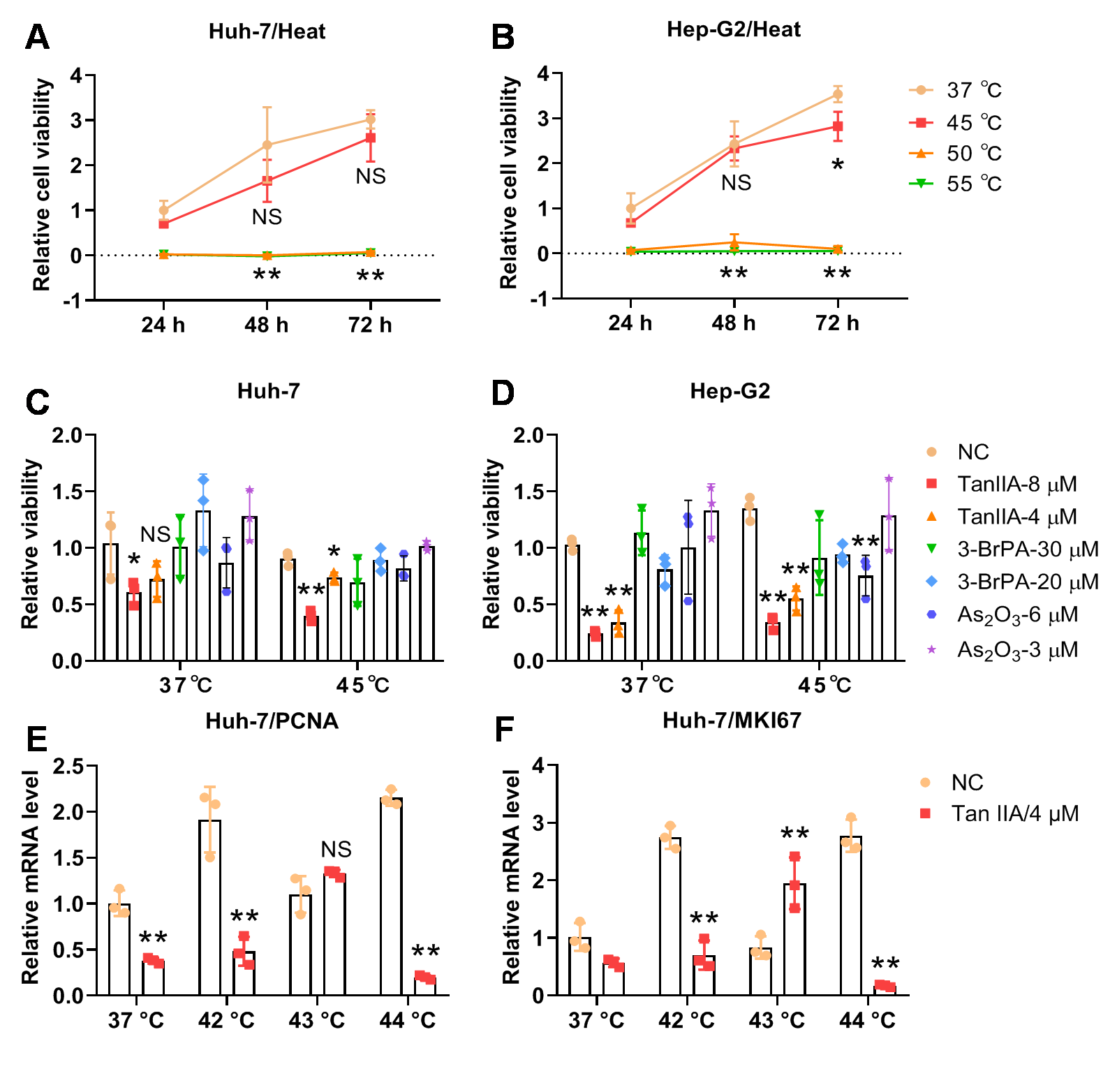

Supplement: Supplementary file 2 — Figure S1 [file 41420_2025_2795_MOESM2_ESM.tif]

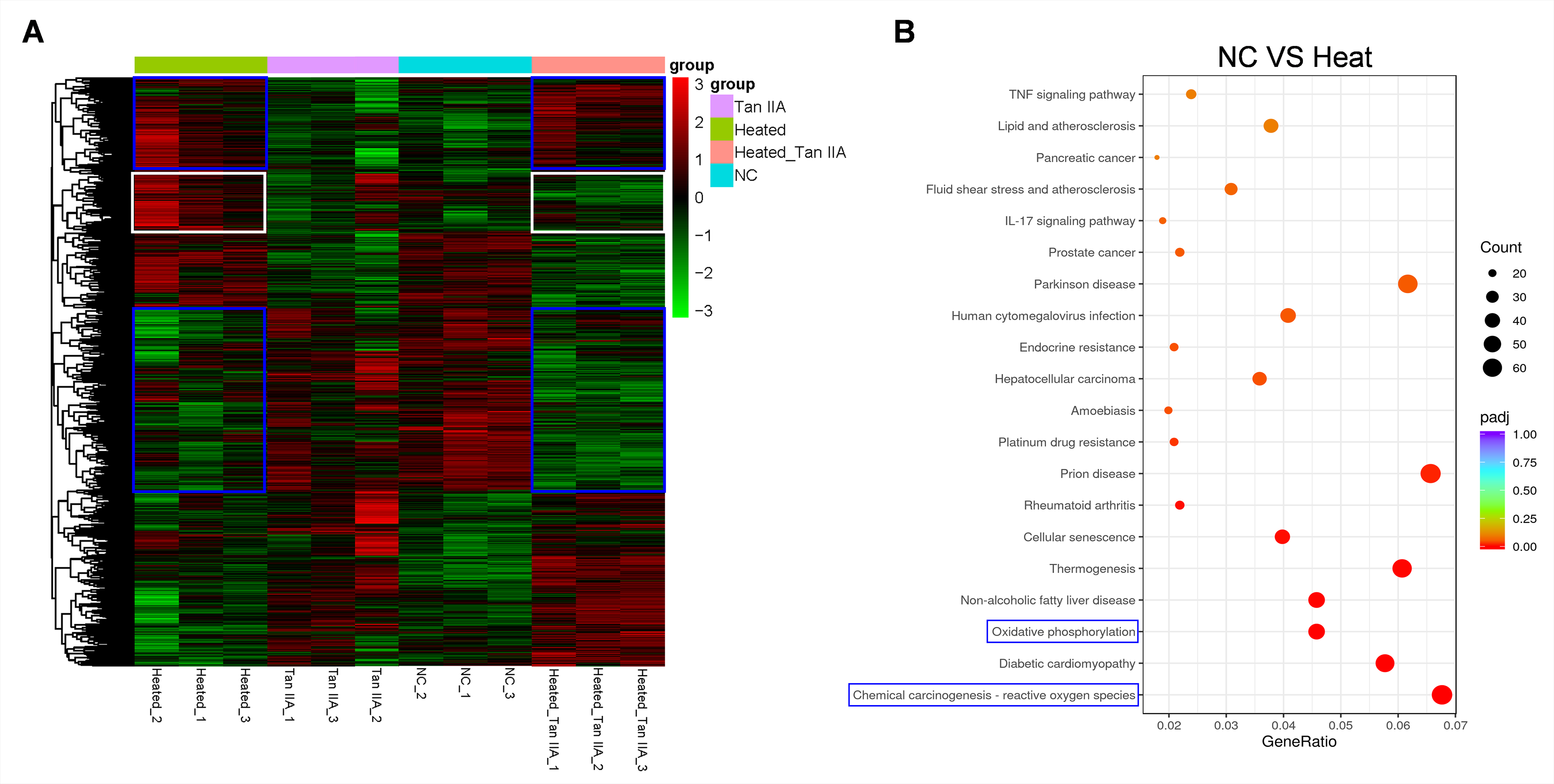

Supplement: Supplementary file 3 — Figure S2 [file 41420_2025_2795_MOESM3_ESM.tif]

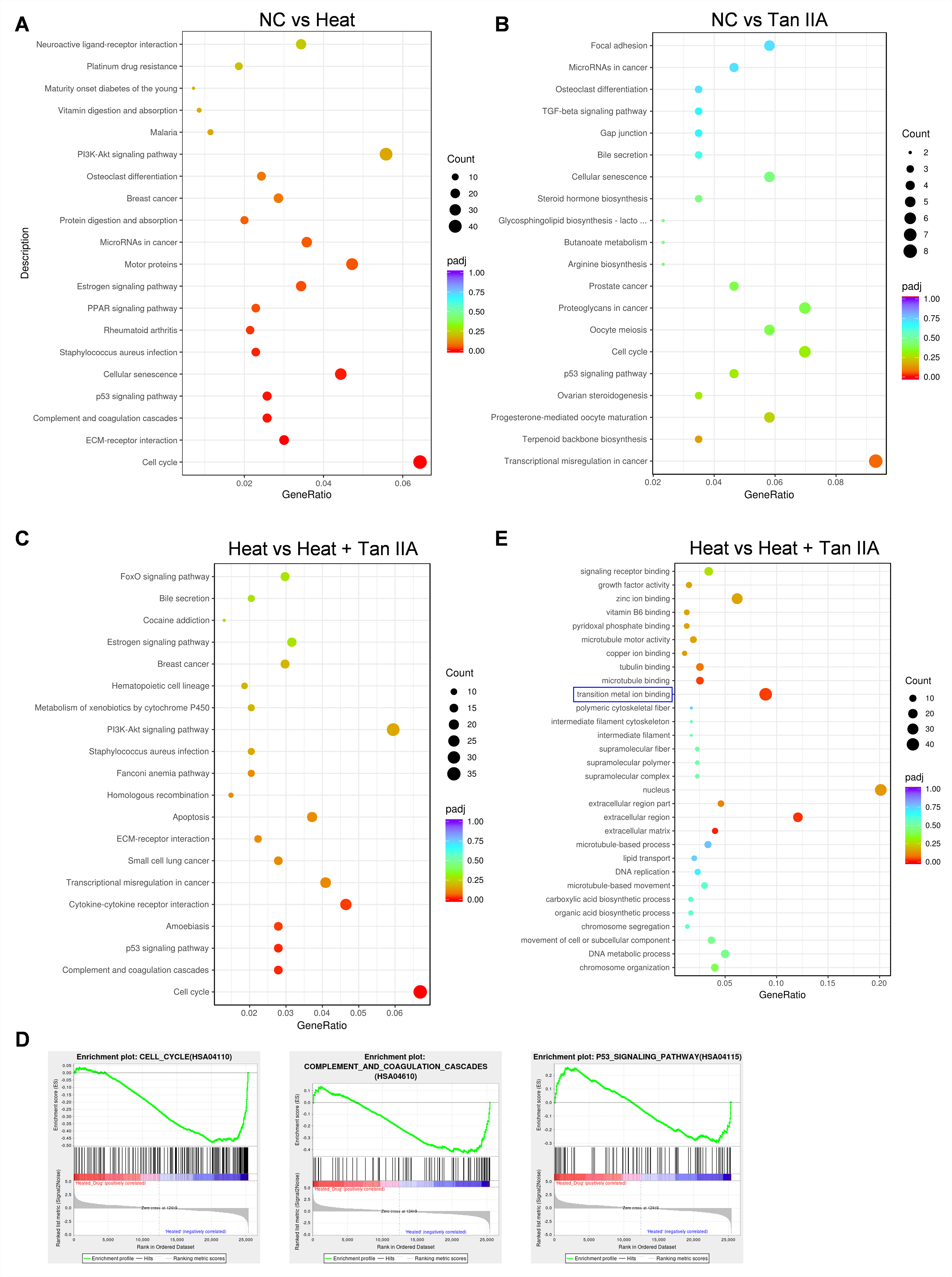

Supplement: Supplementary file 4 — Figure S3 [file 41420_2025_2795_MOESM4_ESM.tif]

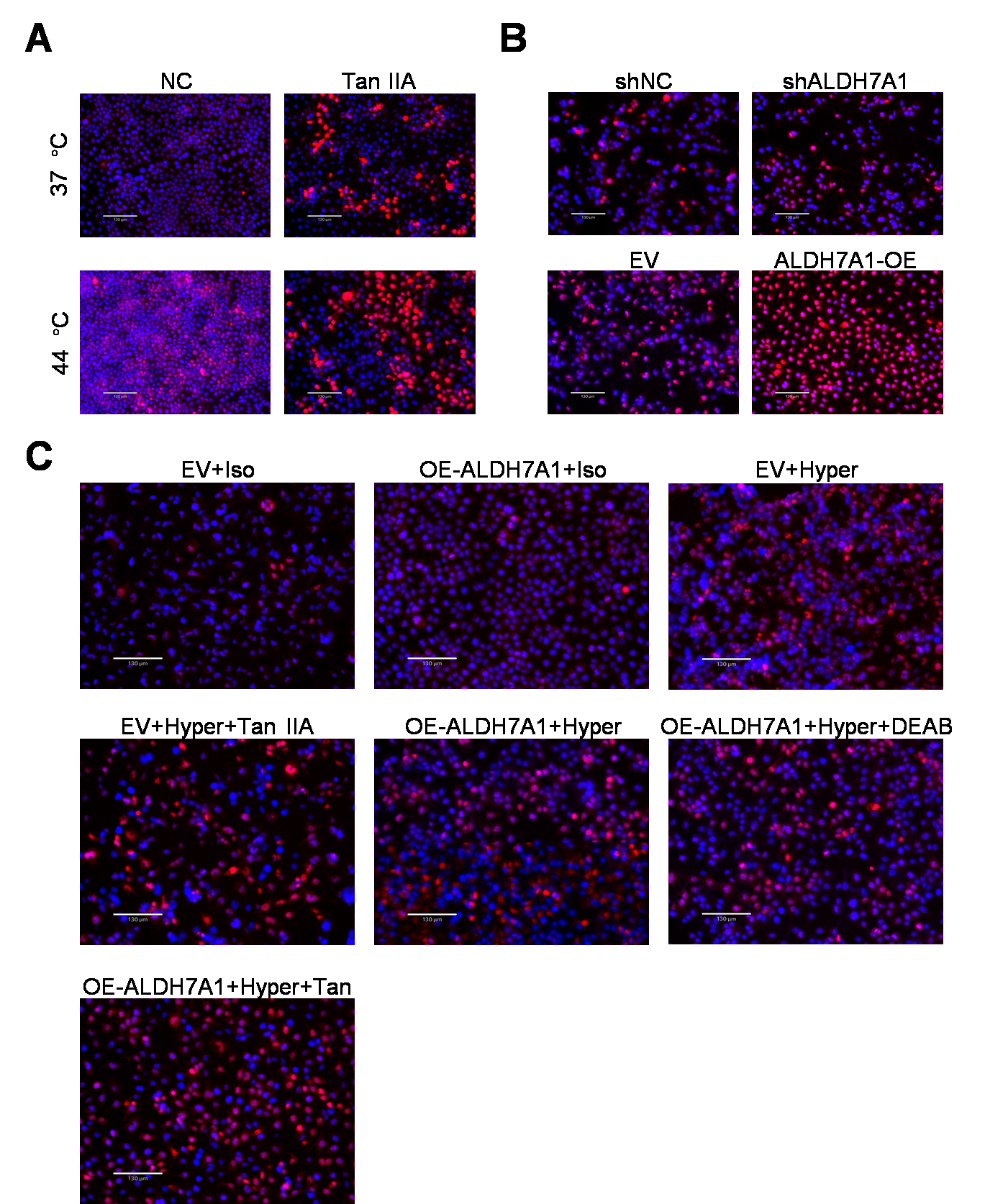

Supplement: Supplementary file 5 — Figure S4 [file 41420_2025_2795_MOESM5_ESM.tif]

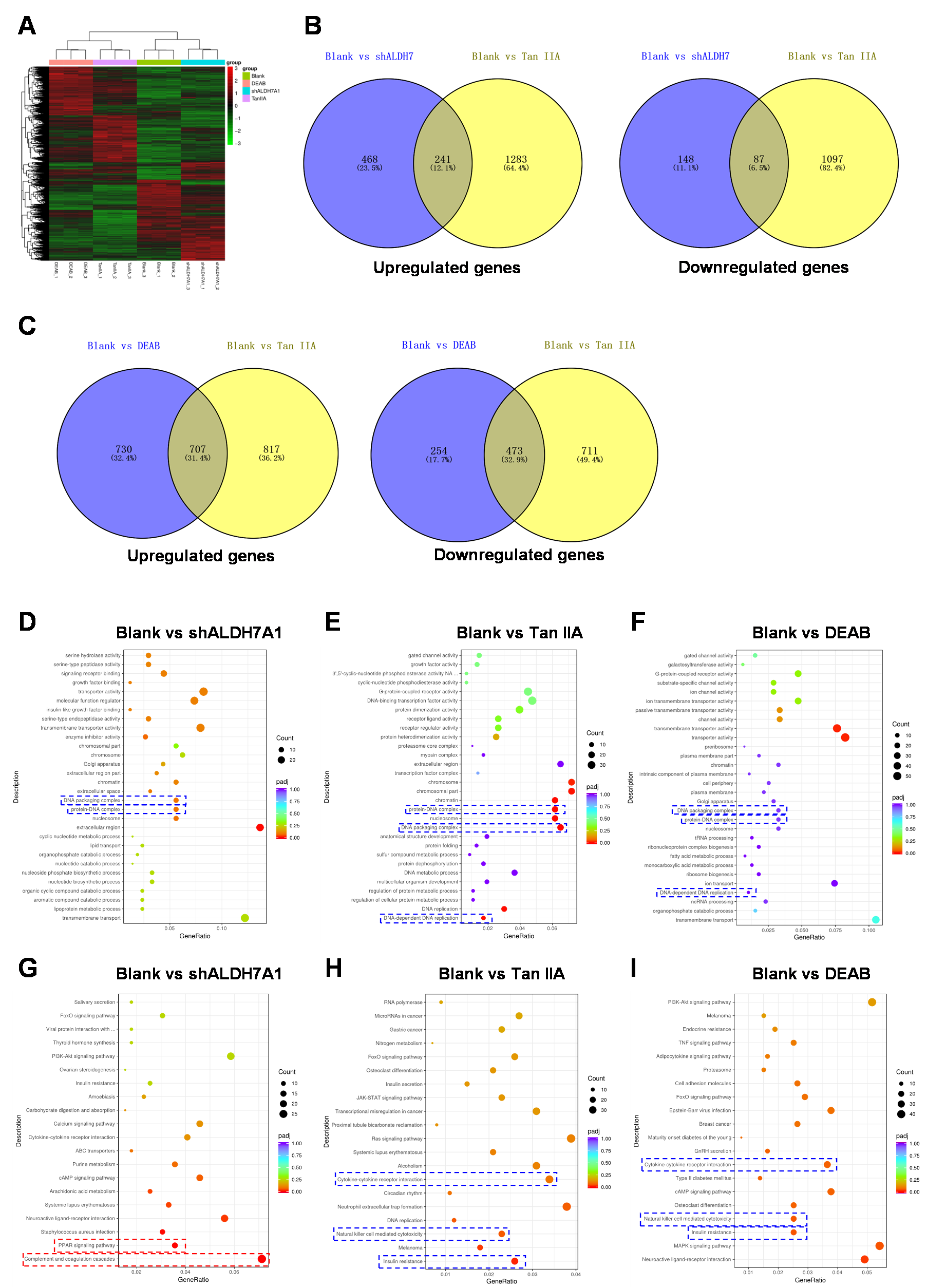

Supplement: Supplementary file 6 — Figure S5 [file 41420_2025_2795_MOESM6_ESM.tif]
