# Supplementary material for: Tanshinone IIA inhibits heat-induced growth of p53-mutant Huh-7 hepatocellular carcinoma by modulating osmotic homeostasis and glycolysis through targeting ALDH7A1
Source: Cell Death Discov. 2025 Oct 31;11:493. doi: 10.1038/s41420-025-02795-0 (PMC12579247; doi:10.1038/s41420-025-02795-0)
Supplement: Supplementary file 7 — Supplementary Table S1 [file 41420_2025_2795_MOESM7_ESM.docx]

**Table S1 Primers used in this study.**

| **Genes** | **Primers (5’-3’)** | |
| --- | --- | --- |
| GAPDH | Forward | GTTCGTCATGGGTGTGAACC |
|  | Reverse | CATCCACAGTCTTCTGGGTG |
| MAOB | Forward | TGGGAGGCAGGACTTACACT |
|  | Reverse | TGGTGGATCAGACGCTCAAC |
| DOA | Forward | AATCTCGGGCTACAACCTCTT |
|  | Reverse | TCAGCTTCCGAAATCCCAGAA |
| PCNA | Forward | ACACTAAGGGCCGAAGATAACG |
|  | Reverse | ACAGCATCTCCAATATGGCTGA |
| MKI67 | Forward | AGAAGAAGTGGTGCTTCGGAA |
|  | Reverse | AGTTTGCGTGGCCTGTACTAA |
| ALDH7A1 | Forward | CCAGTATGCGTGGCTGAAAGA |
|  | Reverse | CAGGGCAATAGGTCGTAATAACC |
