# Supplementary material for: Tanshinone IIA inhibits heat-induced growth of p53-mutant Huh-7 hepatocellular carcinoma by modulating osmotic homeostasis and glycolysis through targeting ALDH7A1
Source: Cell Death Discov. 2025 Oct 31;11:493. doi: 10.1038/s41420-025-02795-0 (PMC12579247; doi:10.1038/s41420-025-02795-0)
Supplement: Supplementary file 10 — Original WB images [file 41420_2025_2795_MOESM10_ESM.pptx]

## Slide 1
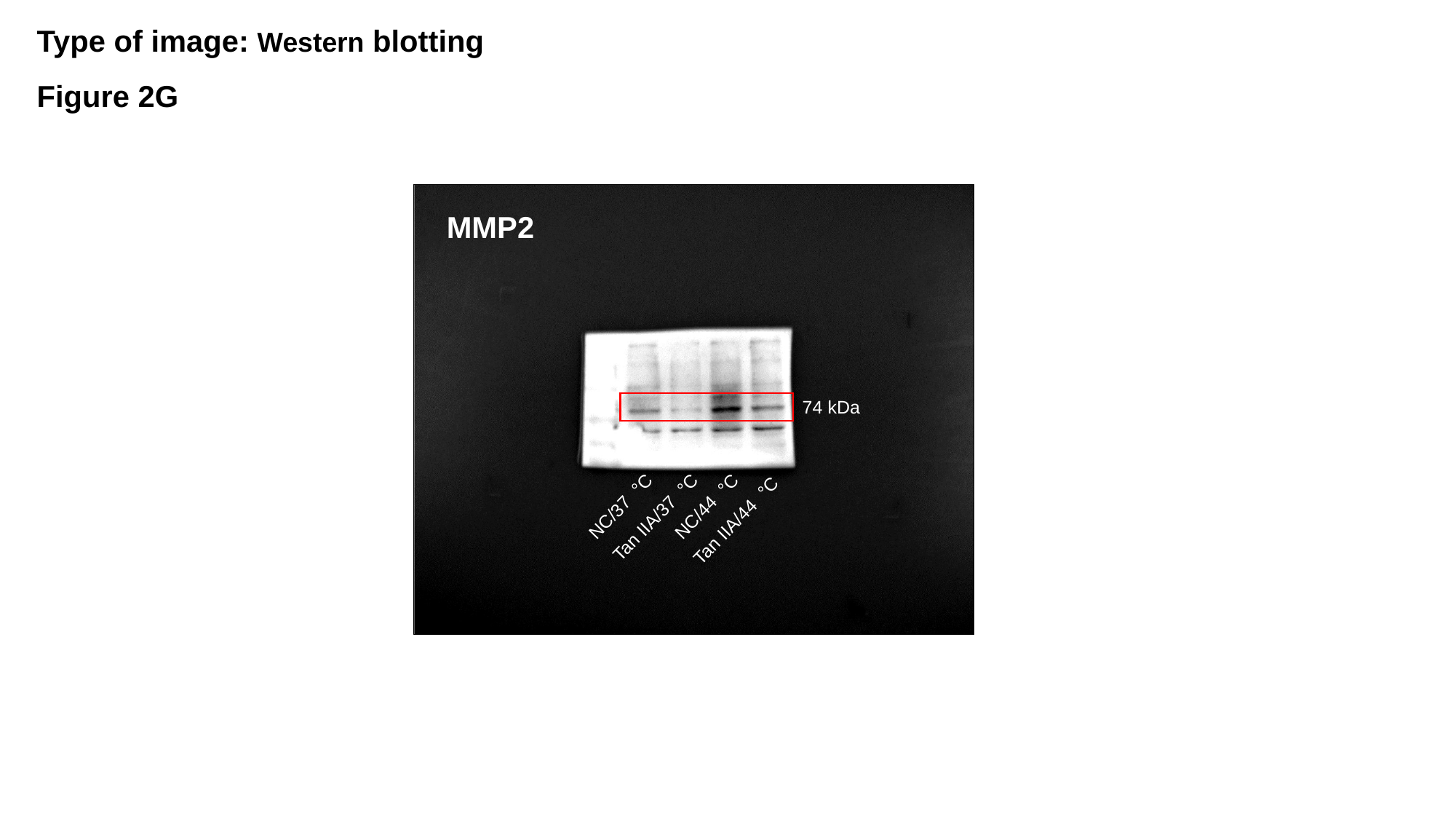

Type of image: Western blotting
Figure 2G
MMP2
74 kDa
NC/44 °C
Tan IIA/37 °C
NC/37 °C
Tan IIA/44 °C

## Slide 2
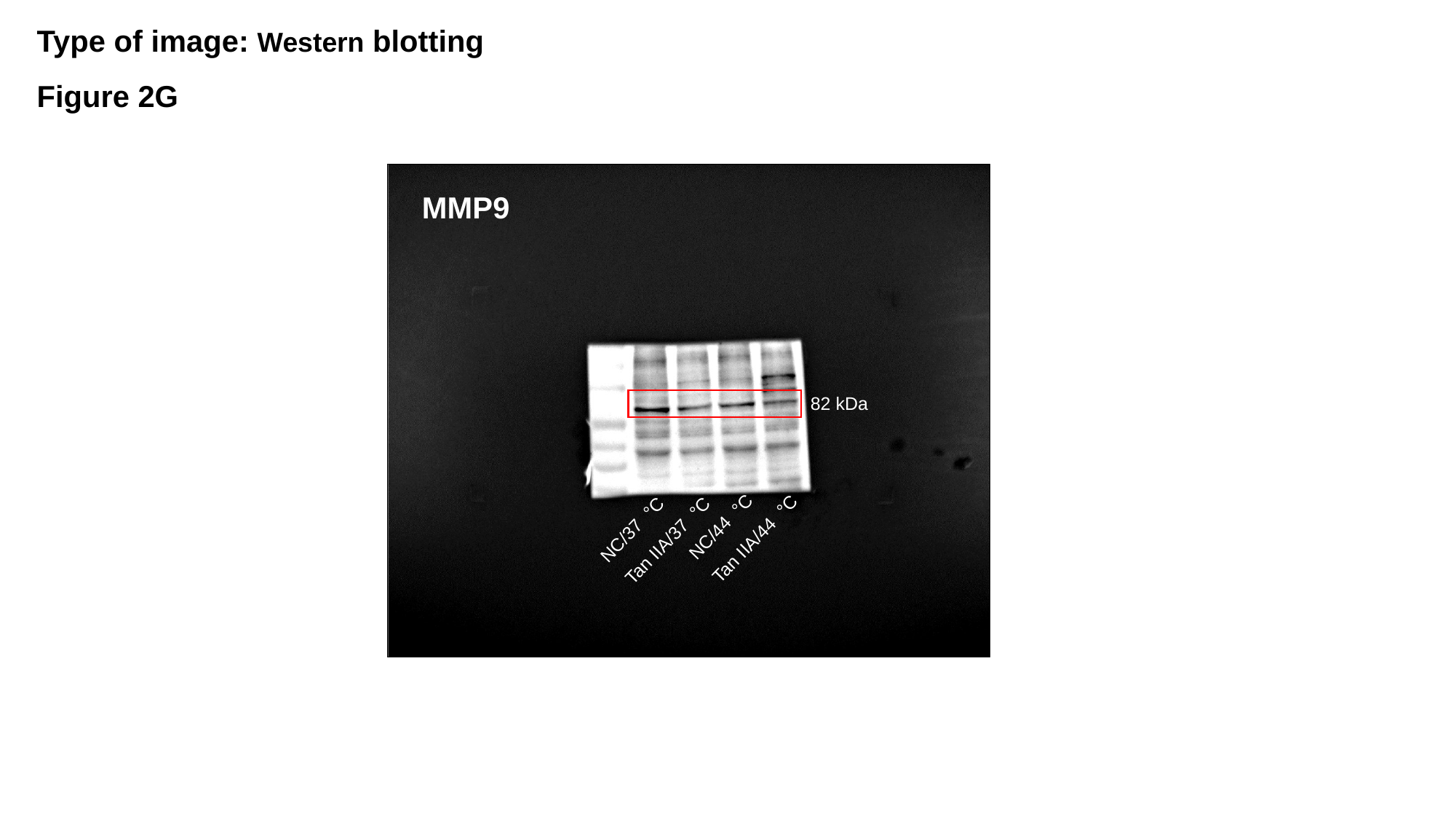

Type of image: Western blotting
Figure 2G
MMP9
82 kDa
NC/44 °C
Tan IIA/37 °C
Tan IIA/44 °C
NC/37 °C

## Slide 3
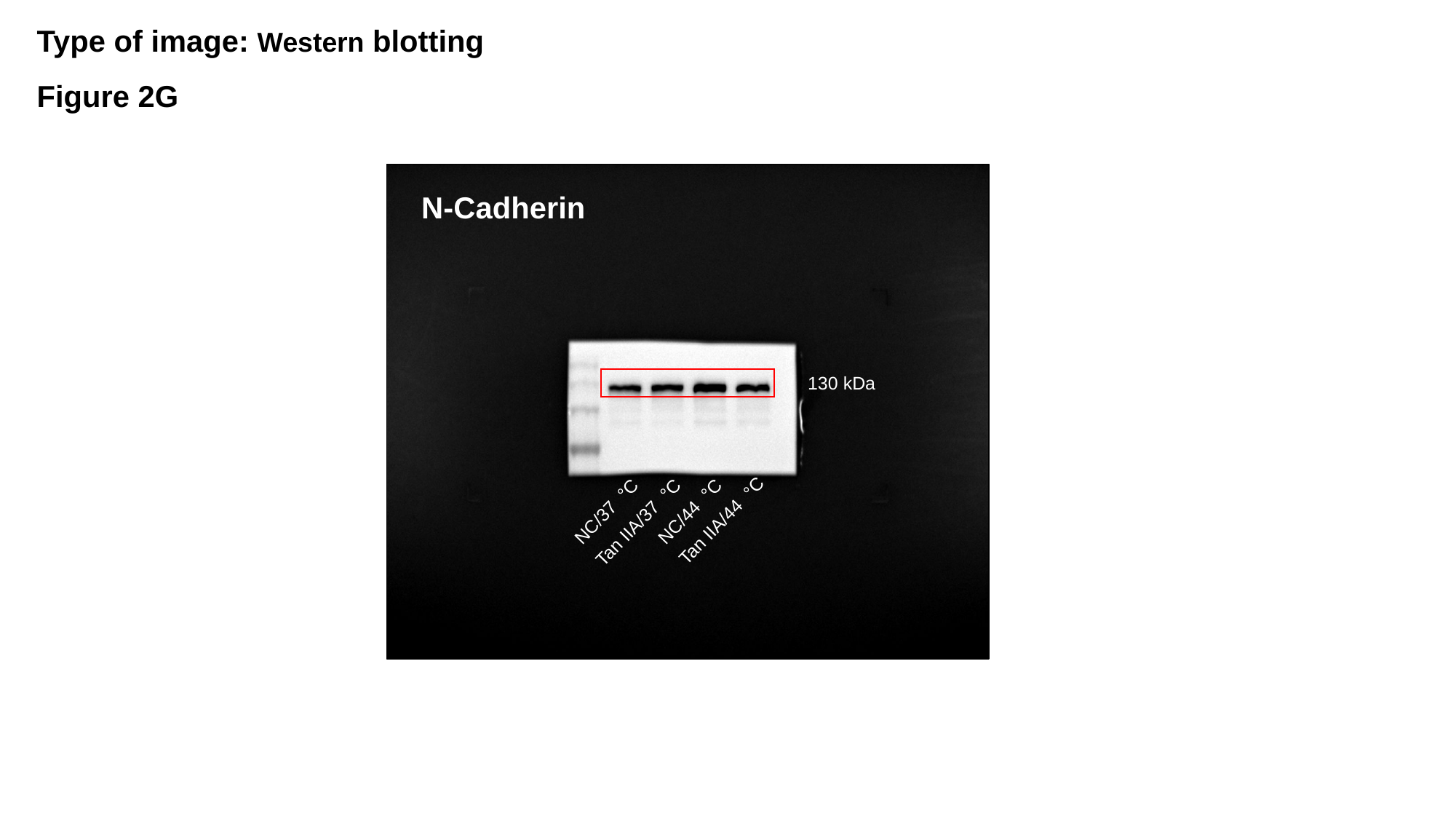

Type of image: Western blotting
Figure 2G
N-Cadherin
130 kDa
NC/44 °C
Tan IIA/37 °C
Tan IIA/44 °C
NC/37 °C

## Slide 4
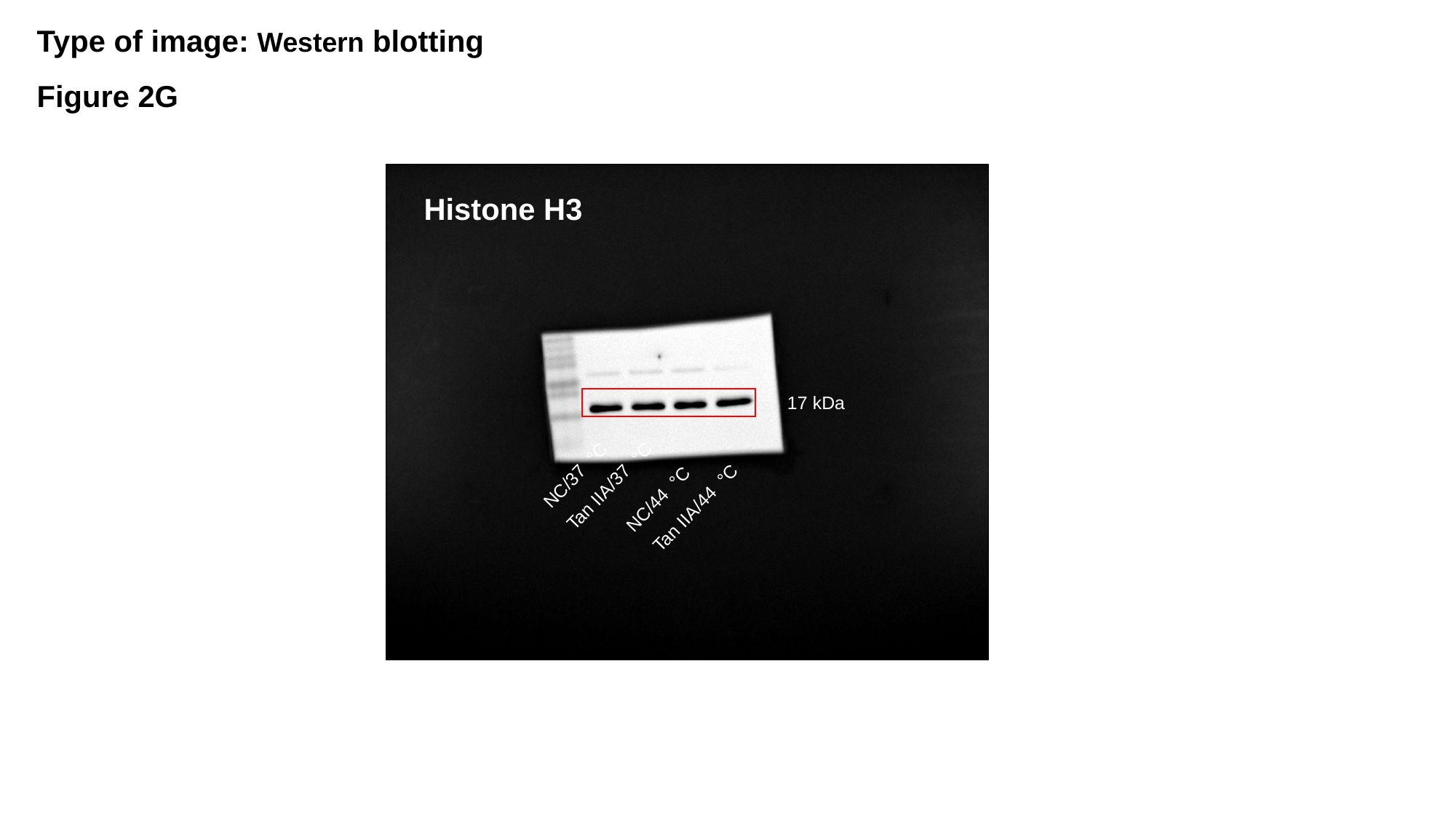

Type of image: Western blotting
Figure 2G
Histone H3
17 kDa
Tan IIA/37 °C
NC/37 °C
NC/44 °C
Tan IIA/44 °C

## Slide 5
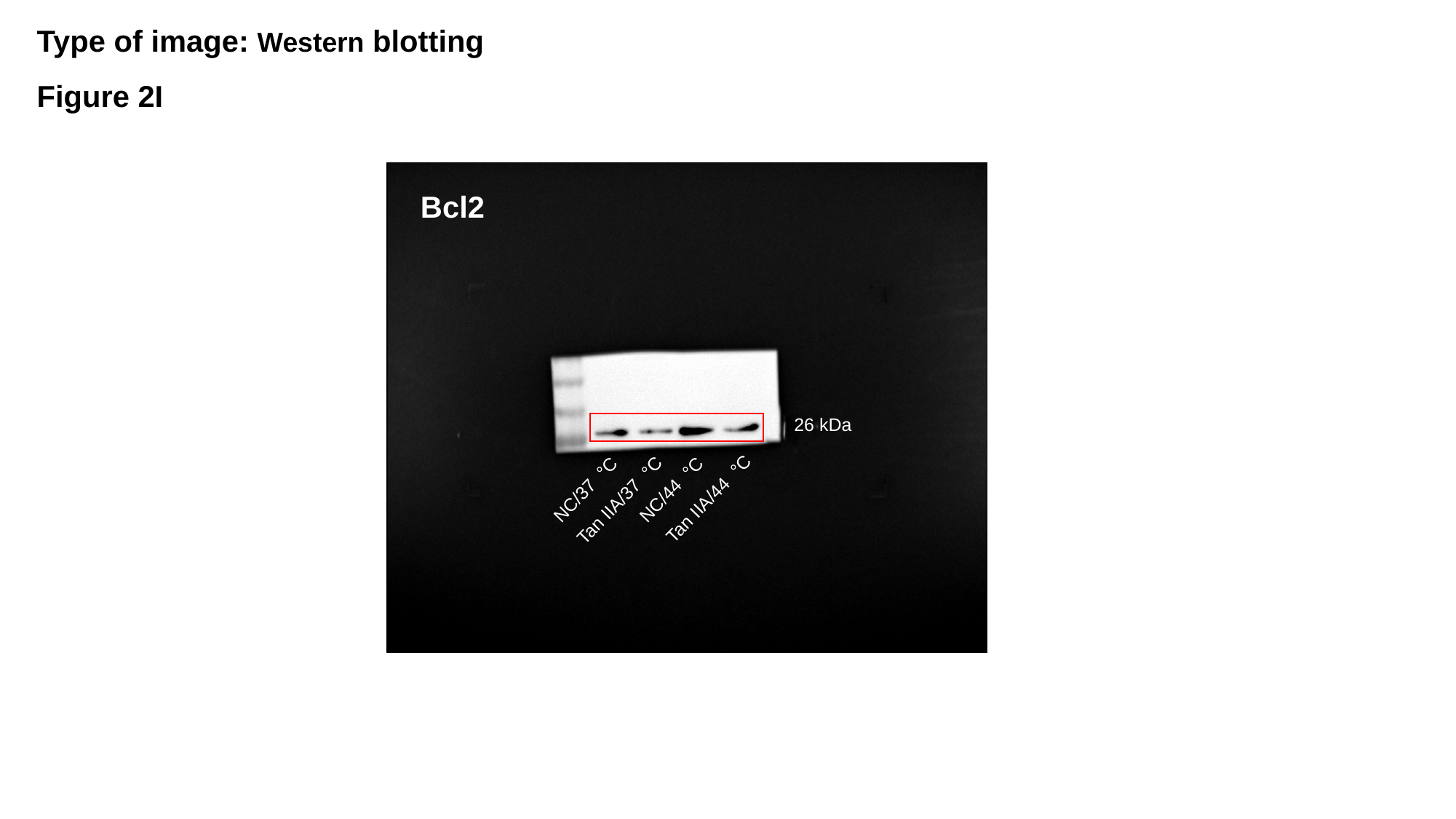

Type of image: Western blotting
Figure 2I
Bcl2
26 kDa
NC/44 °C
Tan IIA/37 °C
Tan IIA/44 °C
NC/37 °C

## Slide 6
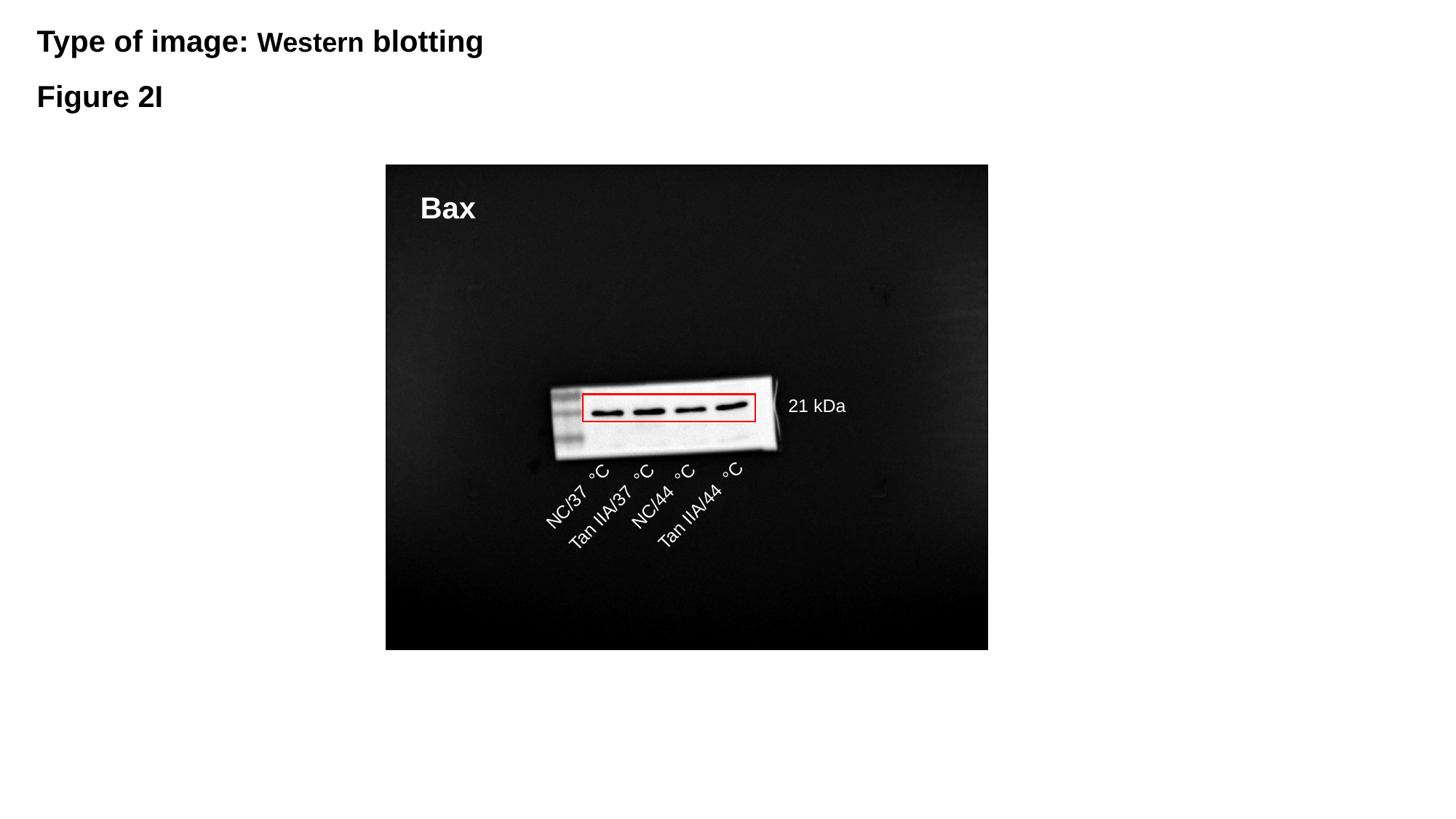

Type of image: Western blotting
Figure 2I
Bax
21 kDa
NC/44 °C
Tan IIA/37 °C
Tan IIA/44 °C
NC/37 °C

## Slide 7
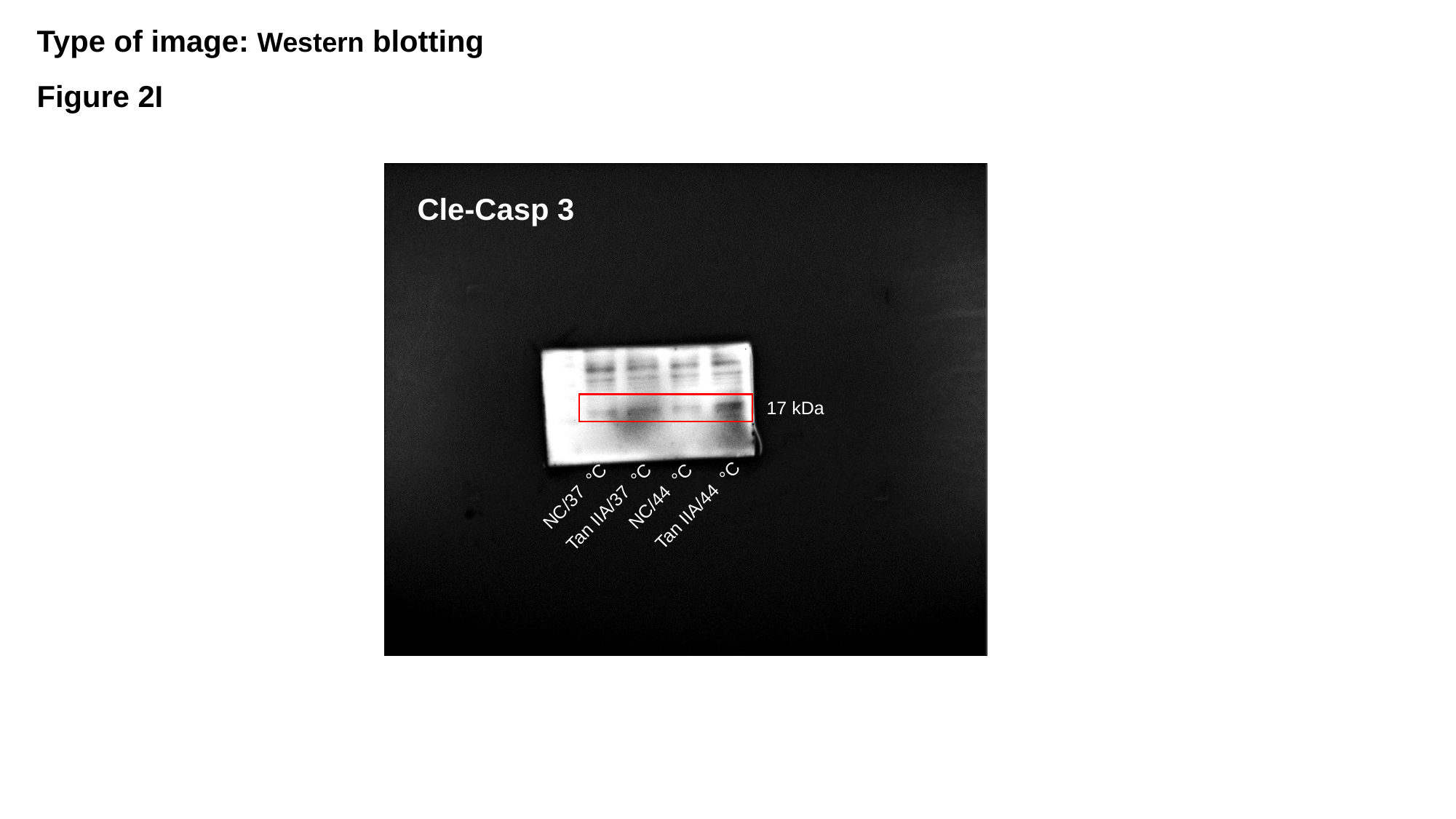

Type of image: Western blotting
Figure 2I
Cle-Casp 3
17 kDa
NC/44 °C
Tan IIA/37 °C
Tan IIA/44 °C
NC/37 °C

## Slide 8
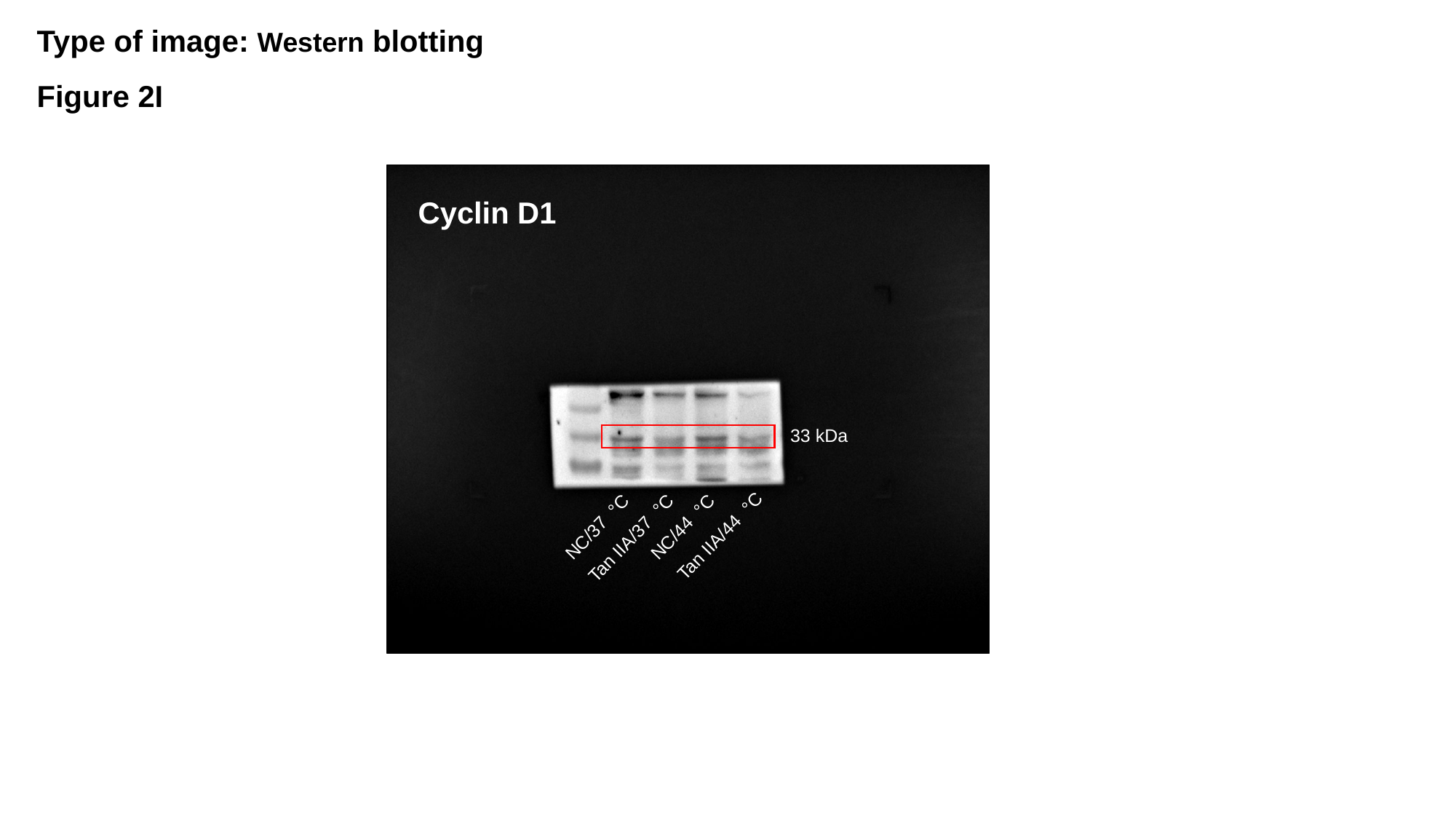

Type of image: Western blotting
Figure 2I
Cyclin D1
33 kDa
NC/44 °C
Tan IIA/37 °C
Tan IIA/44 °C
NC/37 °C

## Slide 9
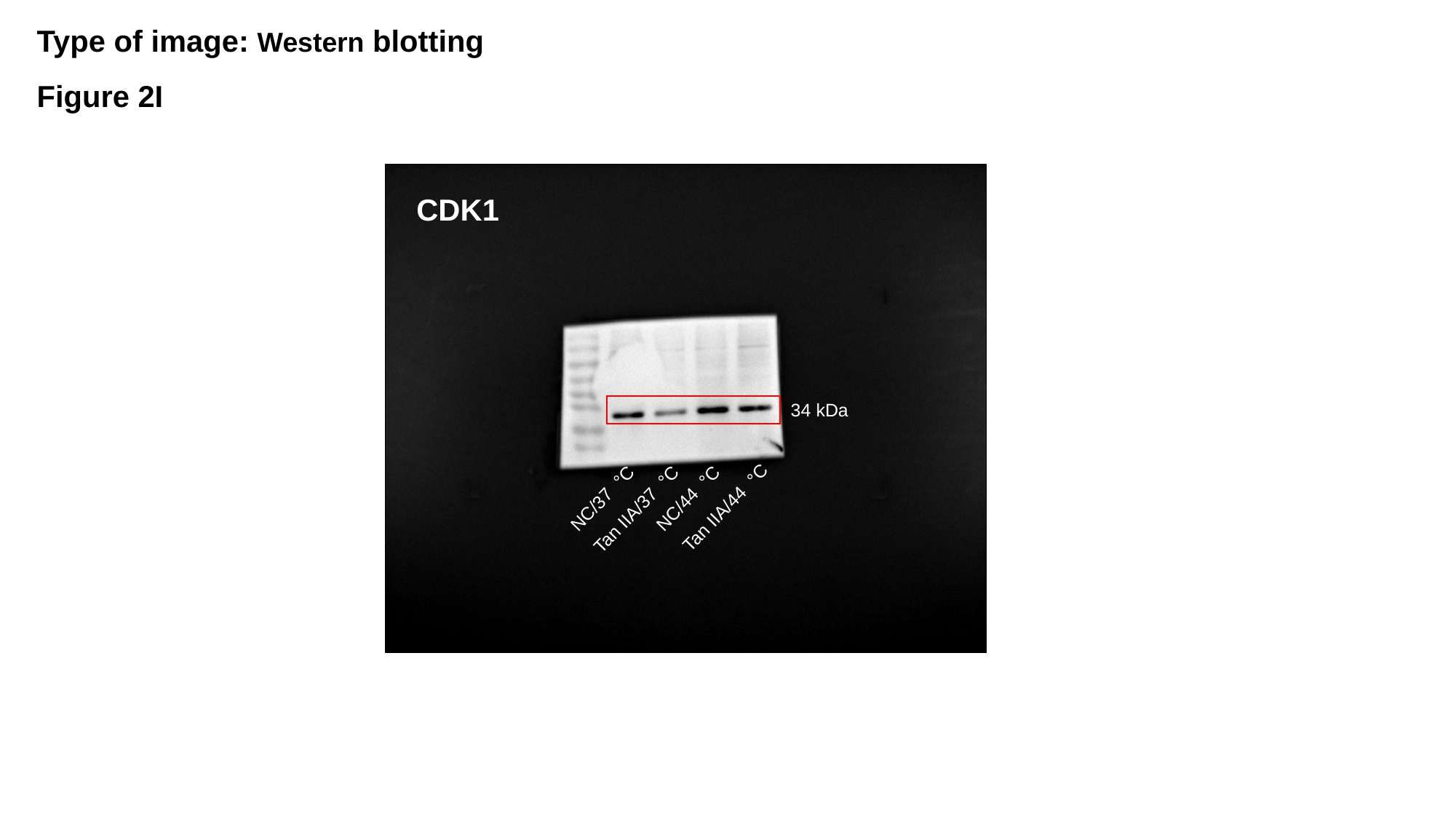

Type of image: Western blotting
Figure 2I
CDK1
34 kDa
NC/44 °C
Tan IIA/37 °C
Tan IIA/44 °C
NC/37 °C

## Slide 10
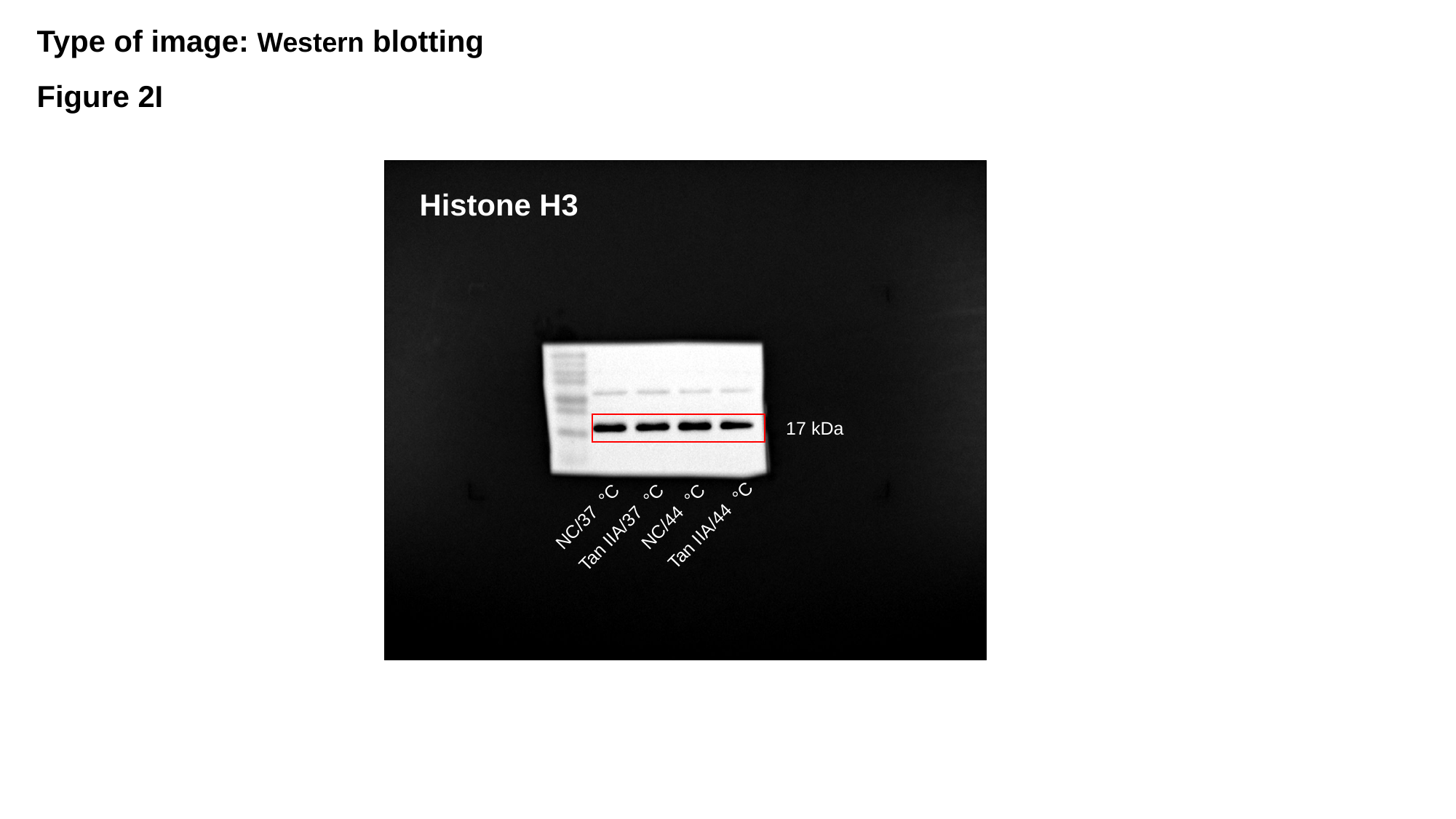

Type of image: Western blotting
Figure 2I
Histone H3
17 kDa
NC/44 °C
Tan IIA/37 °C
Tan IIA/44 °C
NC/37 °C

## Slide 11
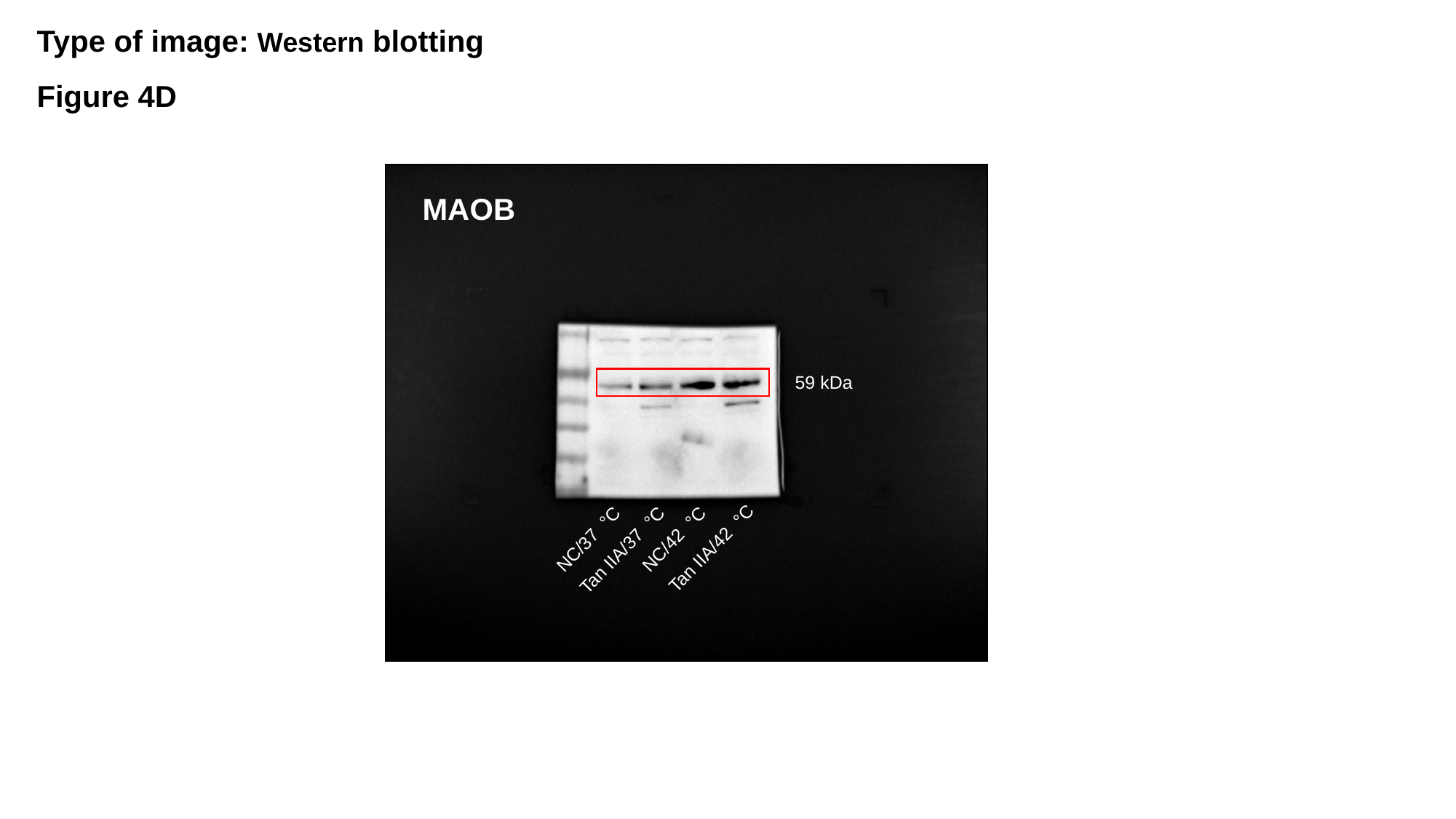

Type of image: Western blotting
Figure 4D
MAOB
59 kDa
NC/42 °C
Tan IIA/37 °C
Tan IIA/42 °C
NC/37 °C

## Slide 12
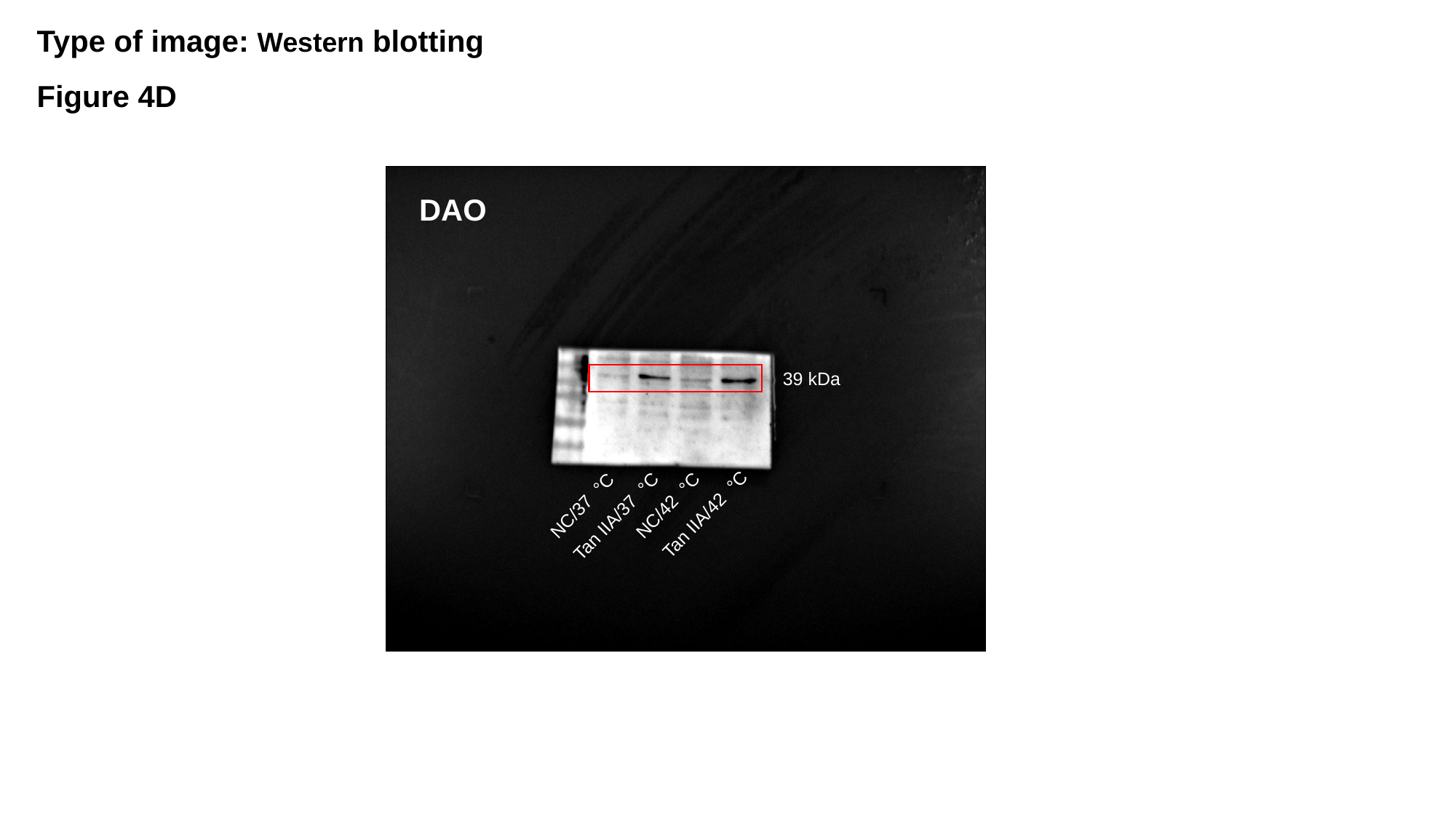

Type of image: Western blotting
Figure 4D
DAO
39 kDa
NC/42 °C
Tan IIA/37 °C
Tan IIA/42 °C
NC/37 °C

## Slide 13
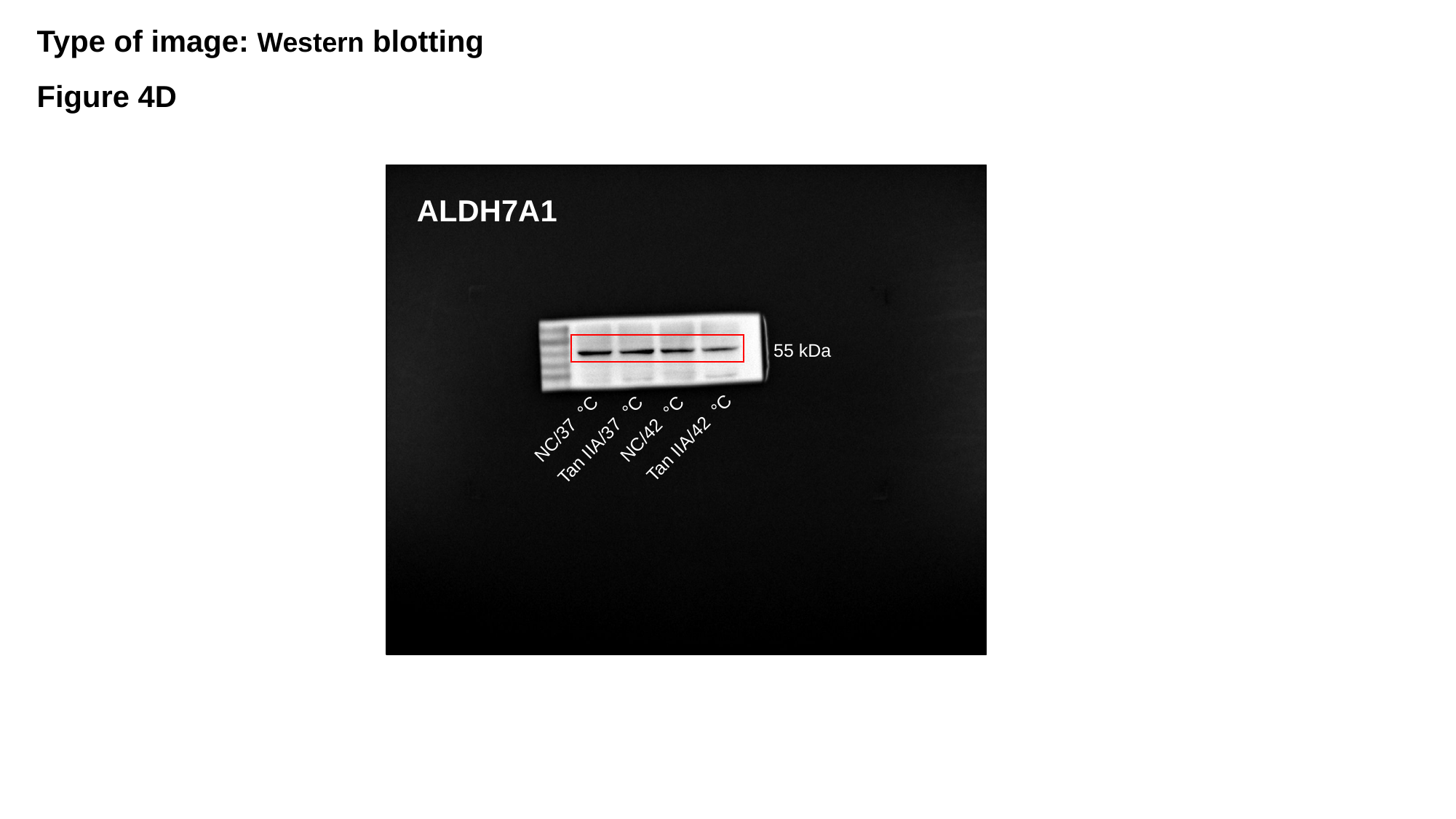

Type of image: Western blotting
Figure 4D
ALDH7A1
55 kDa
NC/42 °C
Tan IIA/37 °C
Tan IIA/42 °C
NC/37 °C

## Slide 14
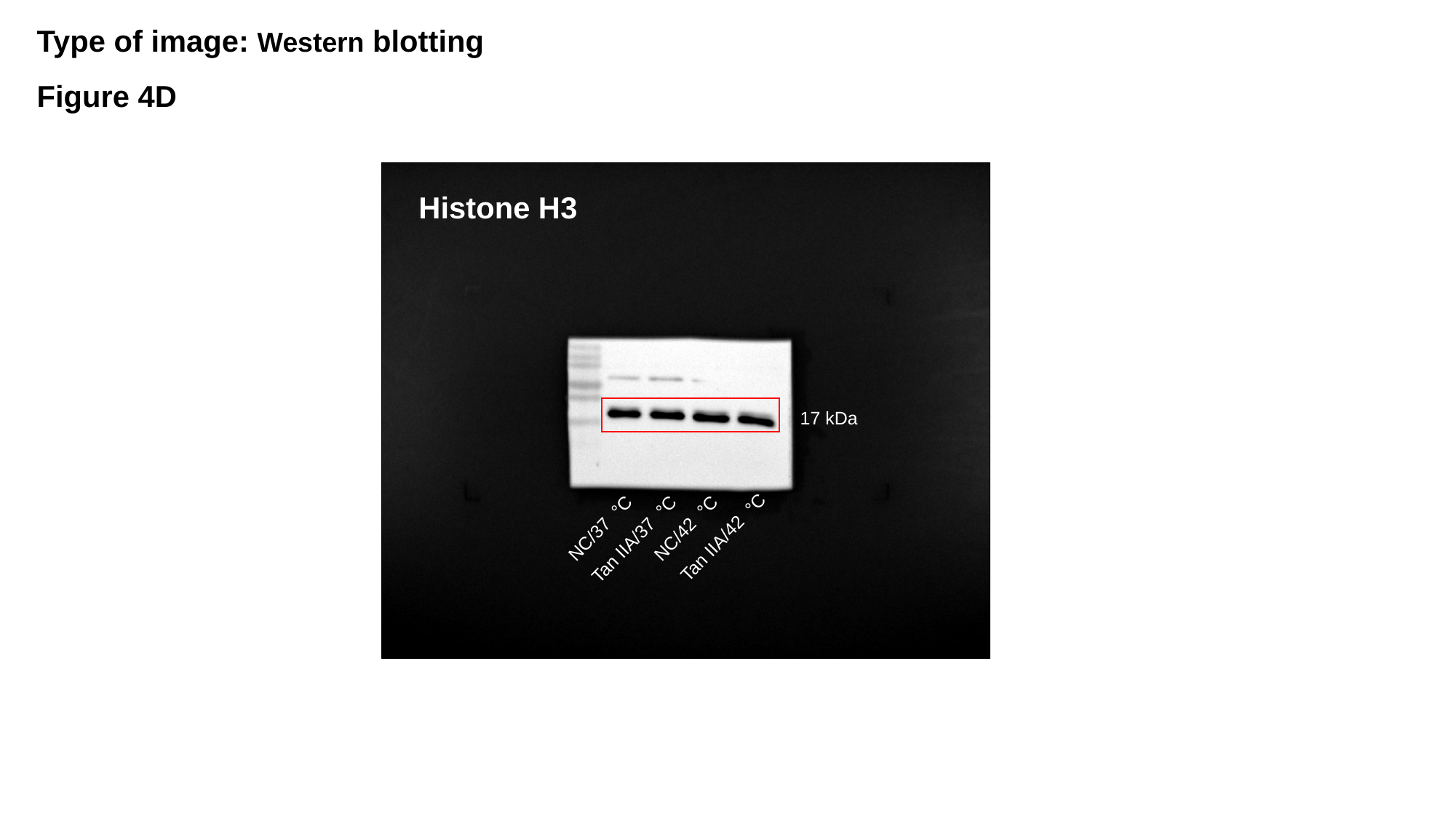

Type of image: Western blotting
Figure 4D
Histone H3
17 kDa
NC/42 °C
Tan IIA/37 °C
Tan IIA/42 °C
NC/37 °C

## Slide 15
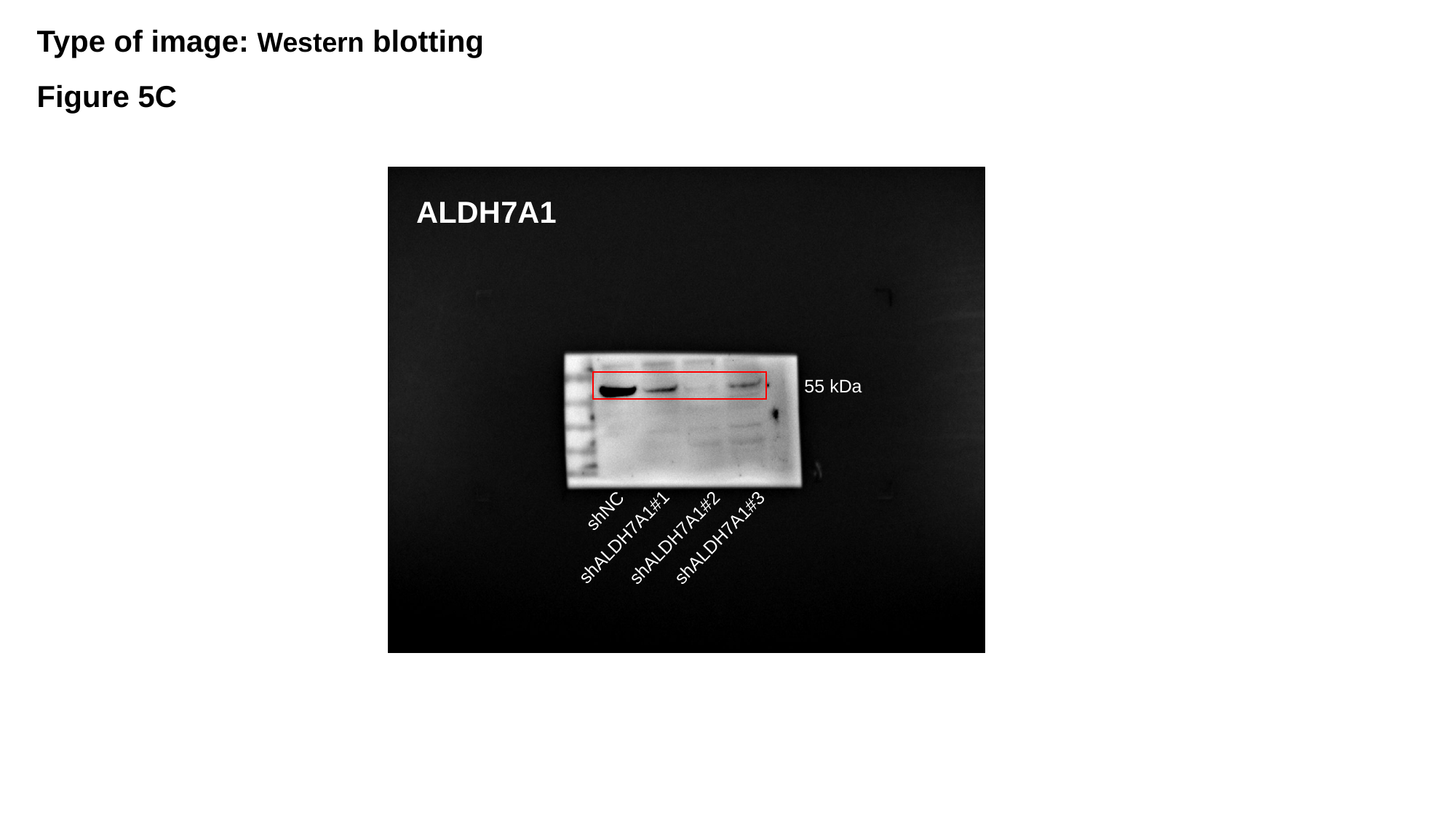

Type of image: Western blotting
Figure 5C
ALDH7A1
55 kDa
shNC
shALDH7A1#1
shALDH7A1#3
shALDH7A1#2

## Slide 16
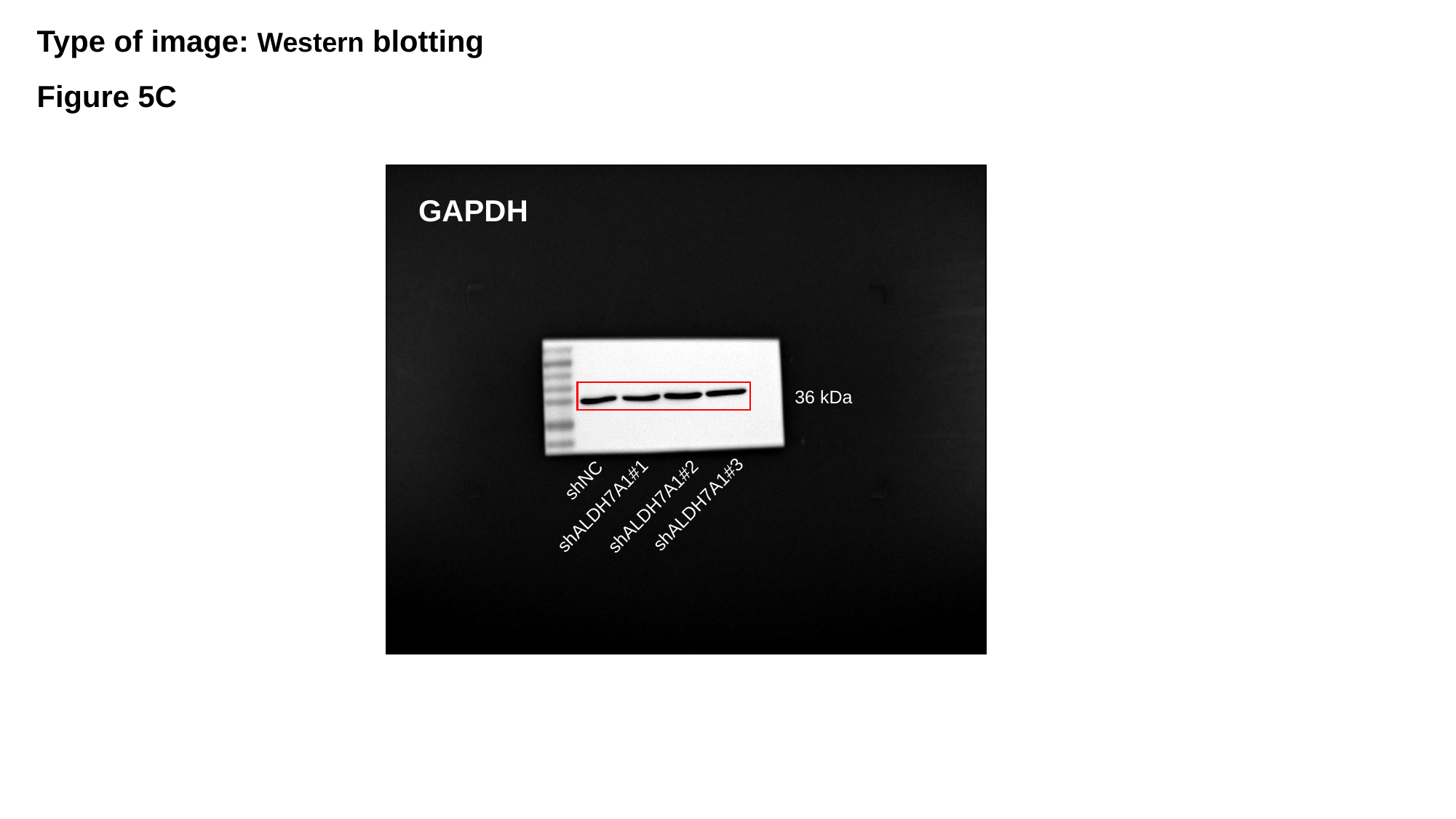

Type of image: Western blotting
Figure 5C
GAPDH
36 kDa
shNC
shALDH7A1#3
shALDH7A1#1
shALDH7A1#2

## Slide 17
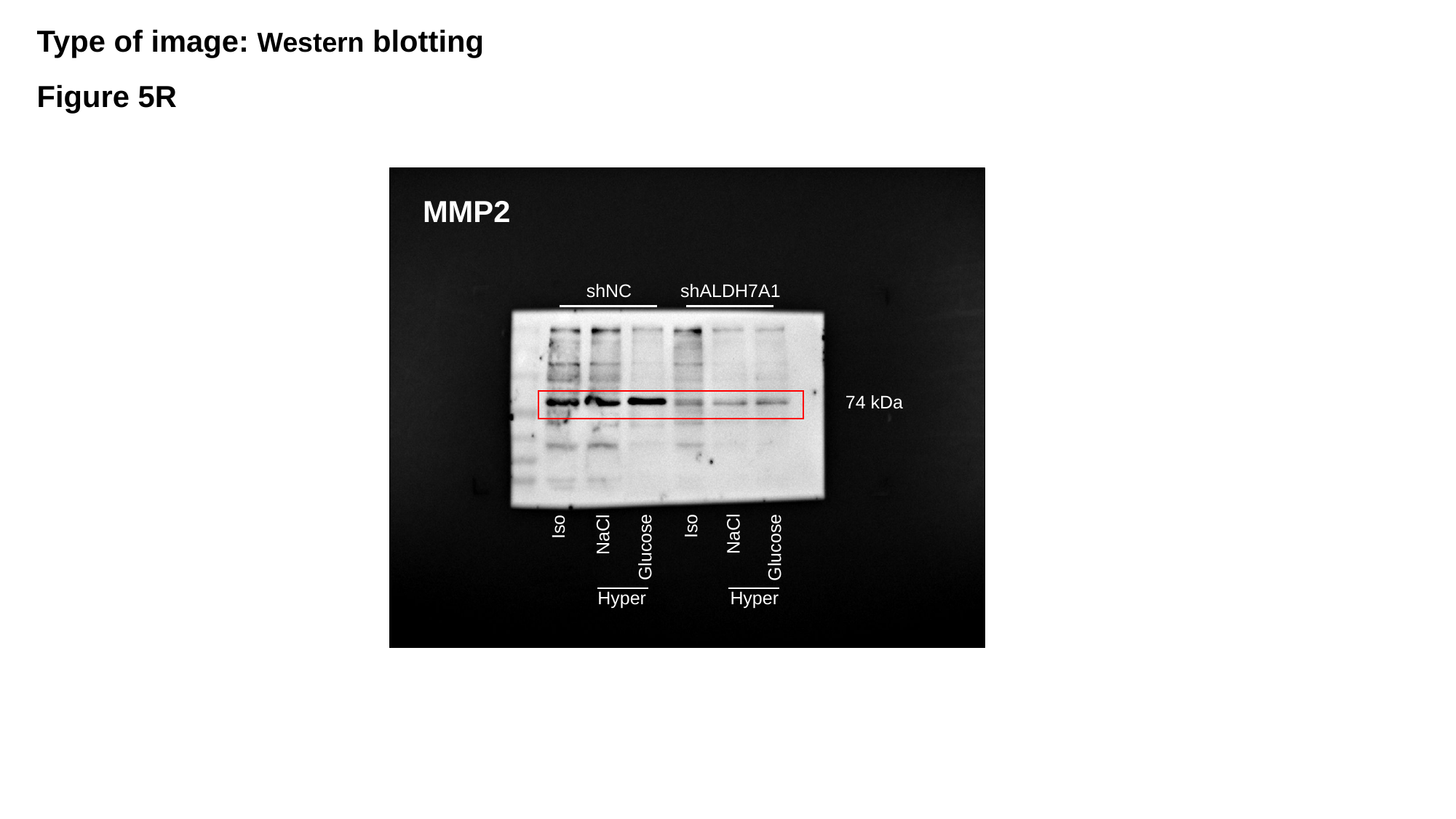

Type of image: Western blotting
Figure 5R
MMP2
shALDH7A1
shNC
74 kDa
Iso
Iso
NaCl
NaCl
Glucose
Glucose
Hyper
Hyper

## Slide 18
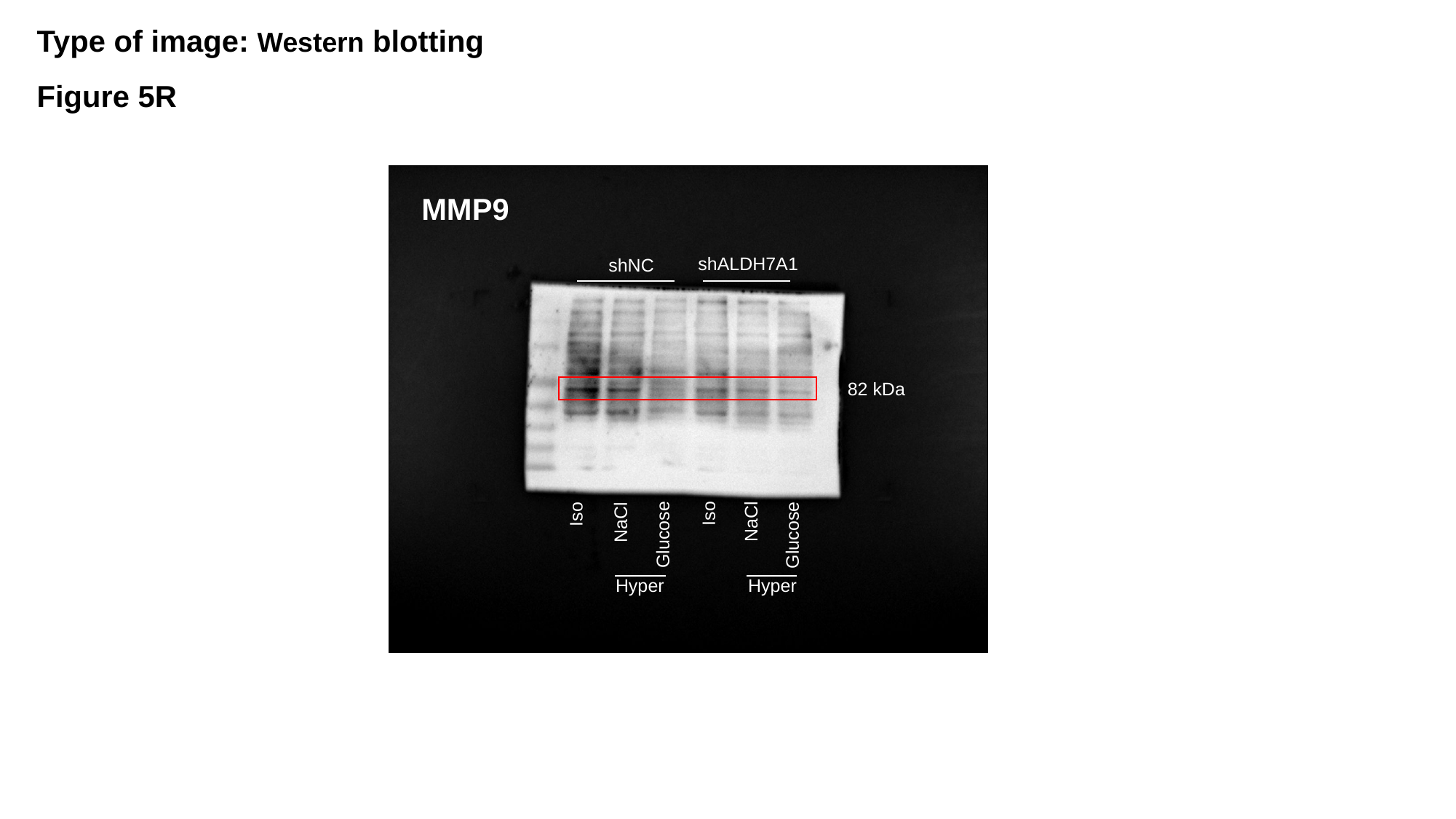

Type of image: Western blotting
Figure 5R
MMP9
shALDH7A1
shNC
82 kDa
Iso
Iso
NaCl
NaCl
Glucose
Glucose
Hyper
Hyper

## Slide 19
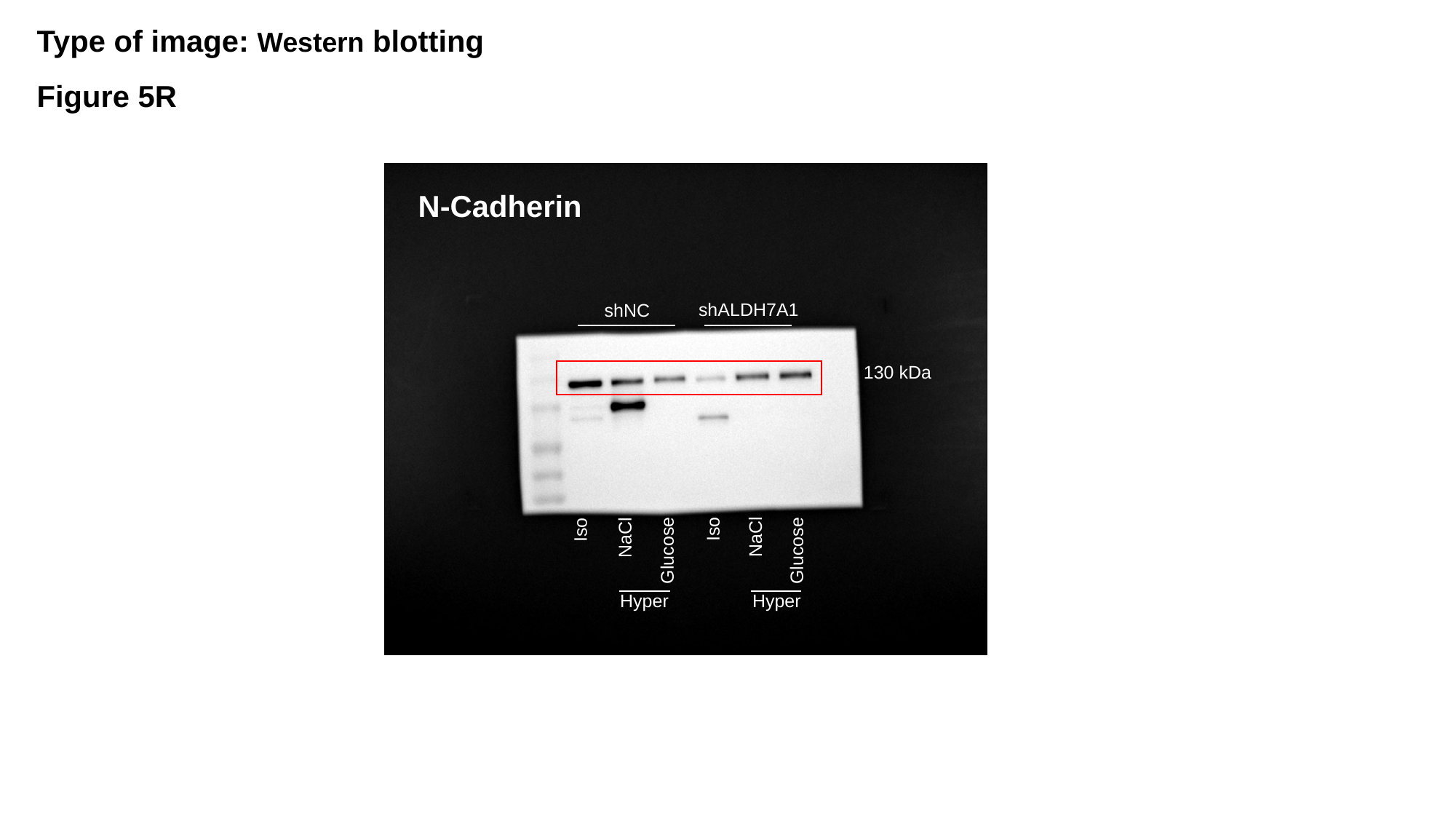

Type of image: Western blotting
Figure 5R
N-Cadherin
shALDH7A1
shNC
130 kDa
Iso
Iso
NaCl
NaCl
Glucose
Glucose
Hyper
Hyper

## Slide 20
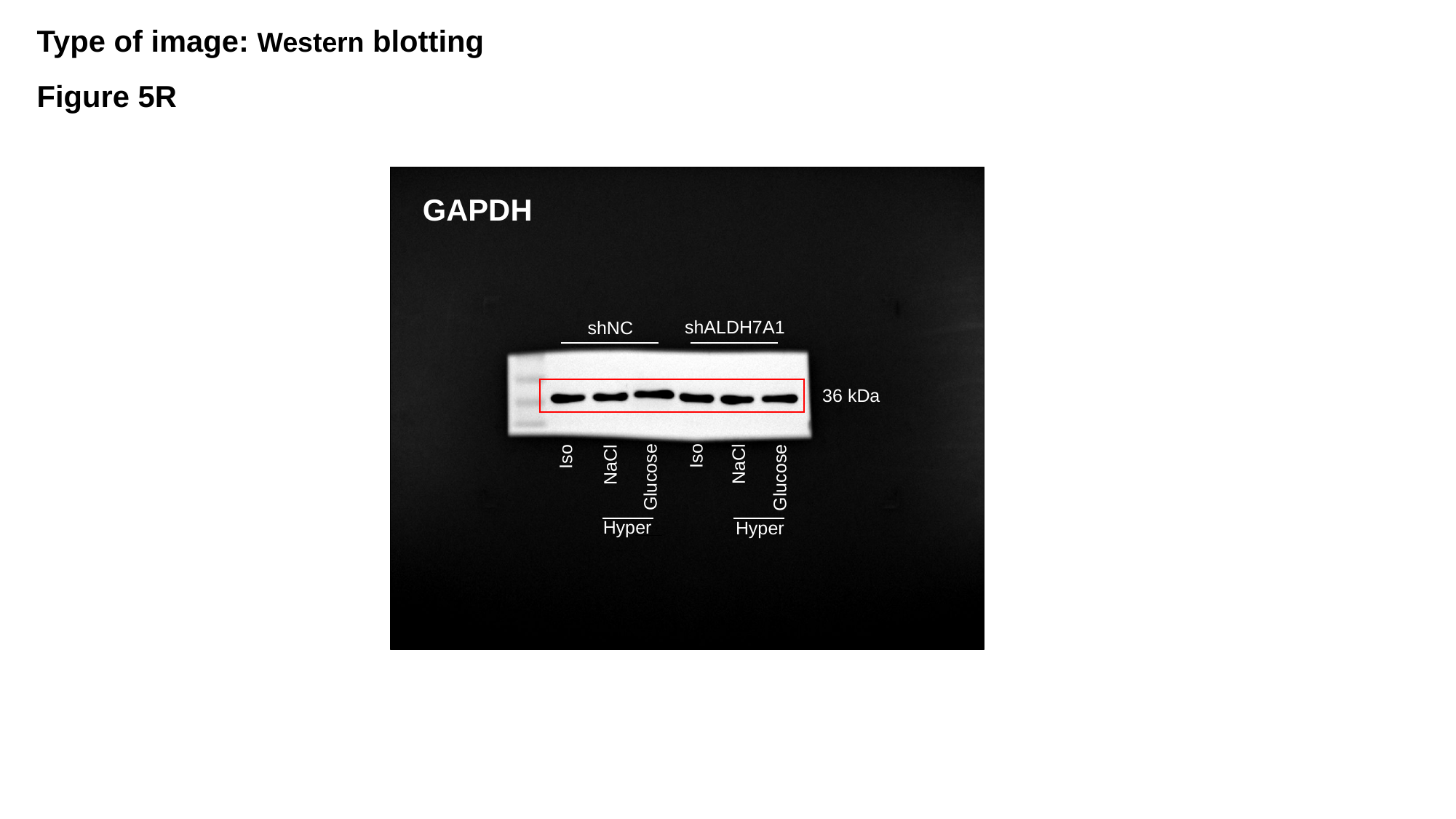

Type of image: Western blotting
Figure 5R
GAPDH
shALDH7A1
shNC
36 kDa
Iso
Iso
NaCl
NaCl
Glucose
Glucose
Hyper
Hyper

## Slide 21
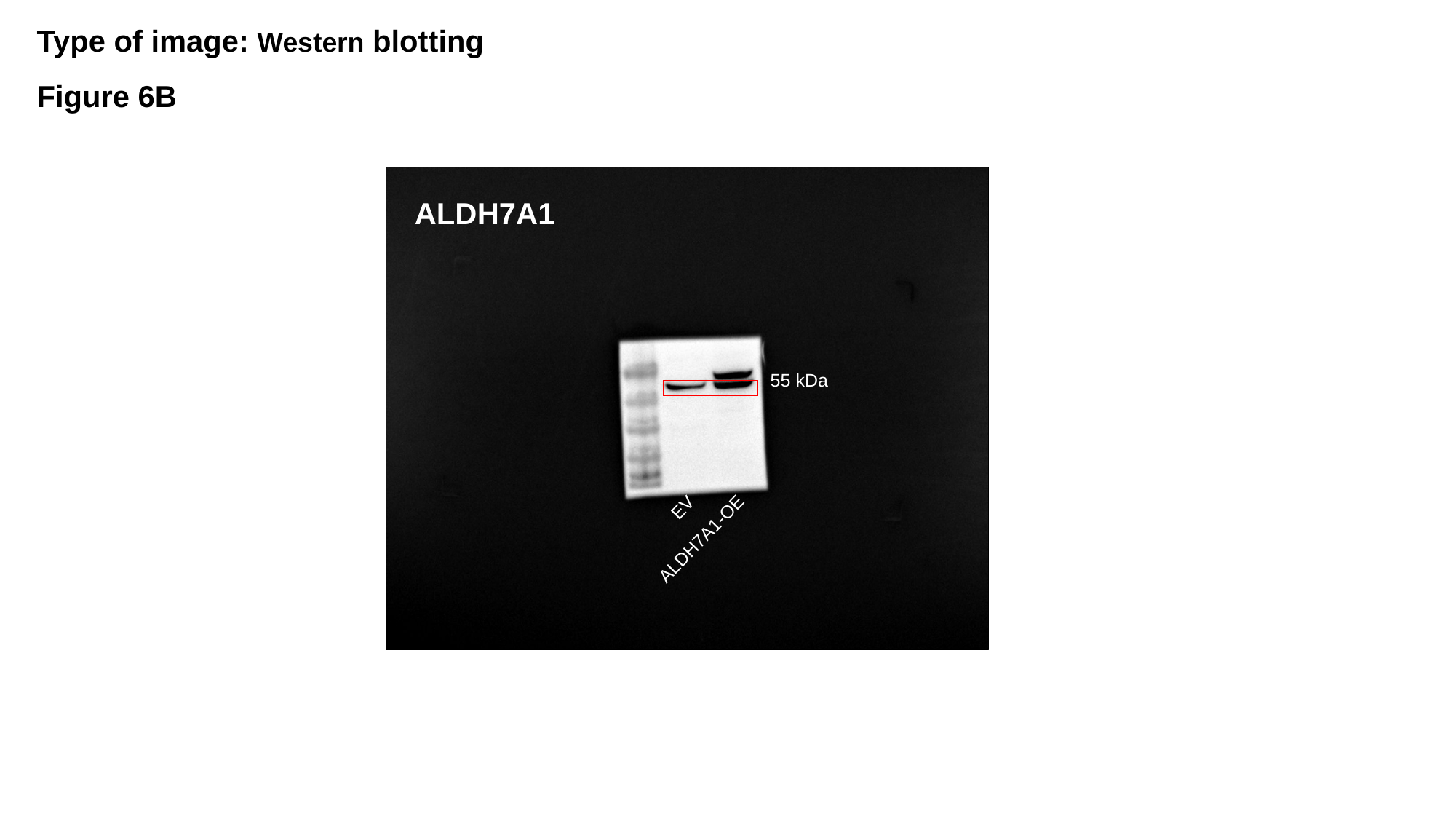

Type of image: Western blotting
Figure 6B
ALDH7A1
55 kDa
EV
ALDH7A1-OE

## Slide 22
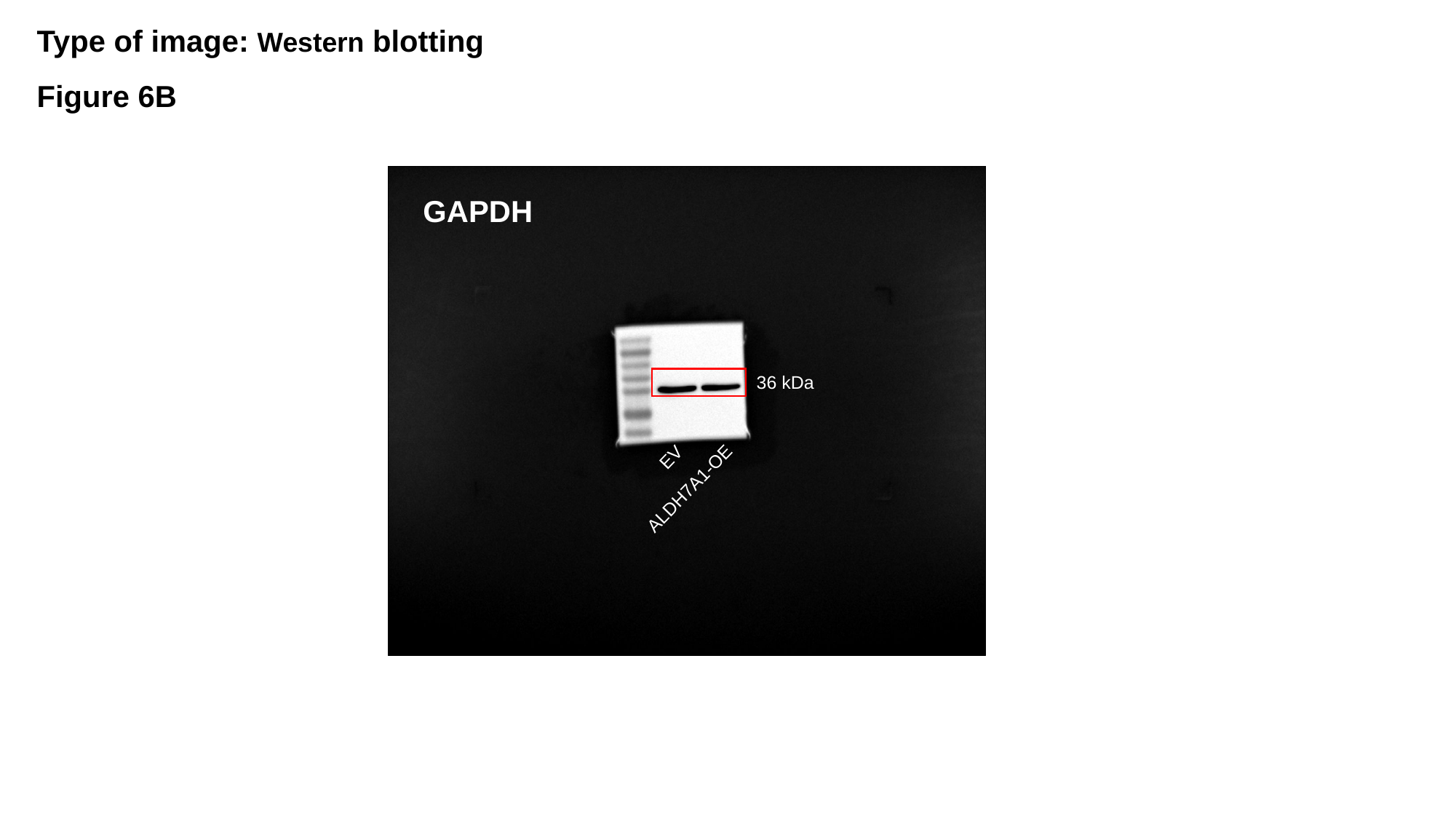

Type of image: Western blotting
Figure 6B
GAPDH
36 kDa
EV
ALDH7A1-OE

## Slide 23
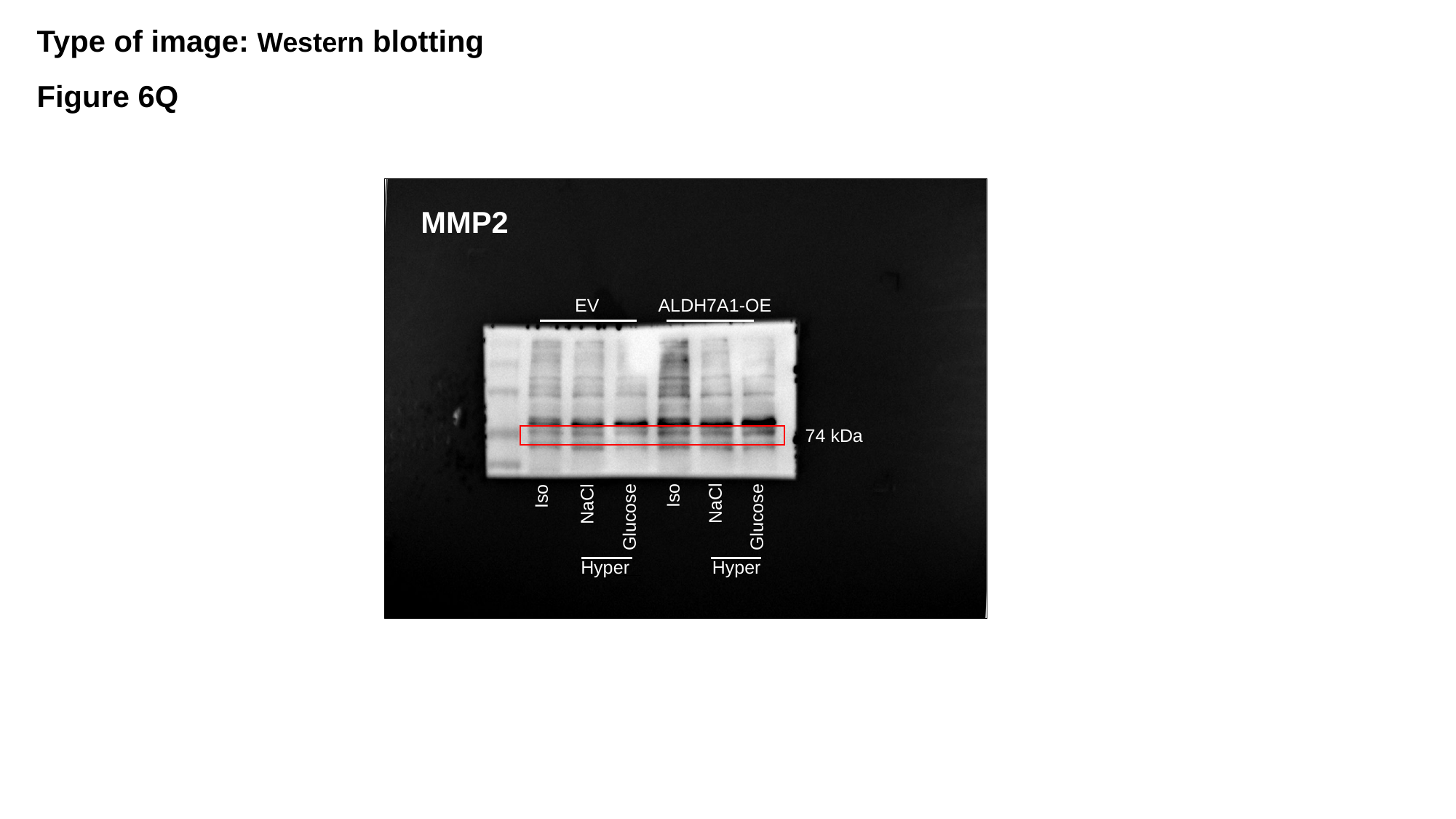

Type of image: Western blotting
Figure 6Q
MMP2
ALDH7A1-OE
EV
74 kDa
Iso
Iso
NaCl
NaCl
Glucose
Glucose
Hyper
Hyper

## Slide 24
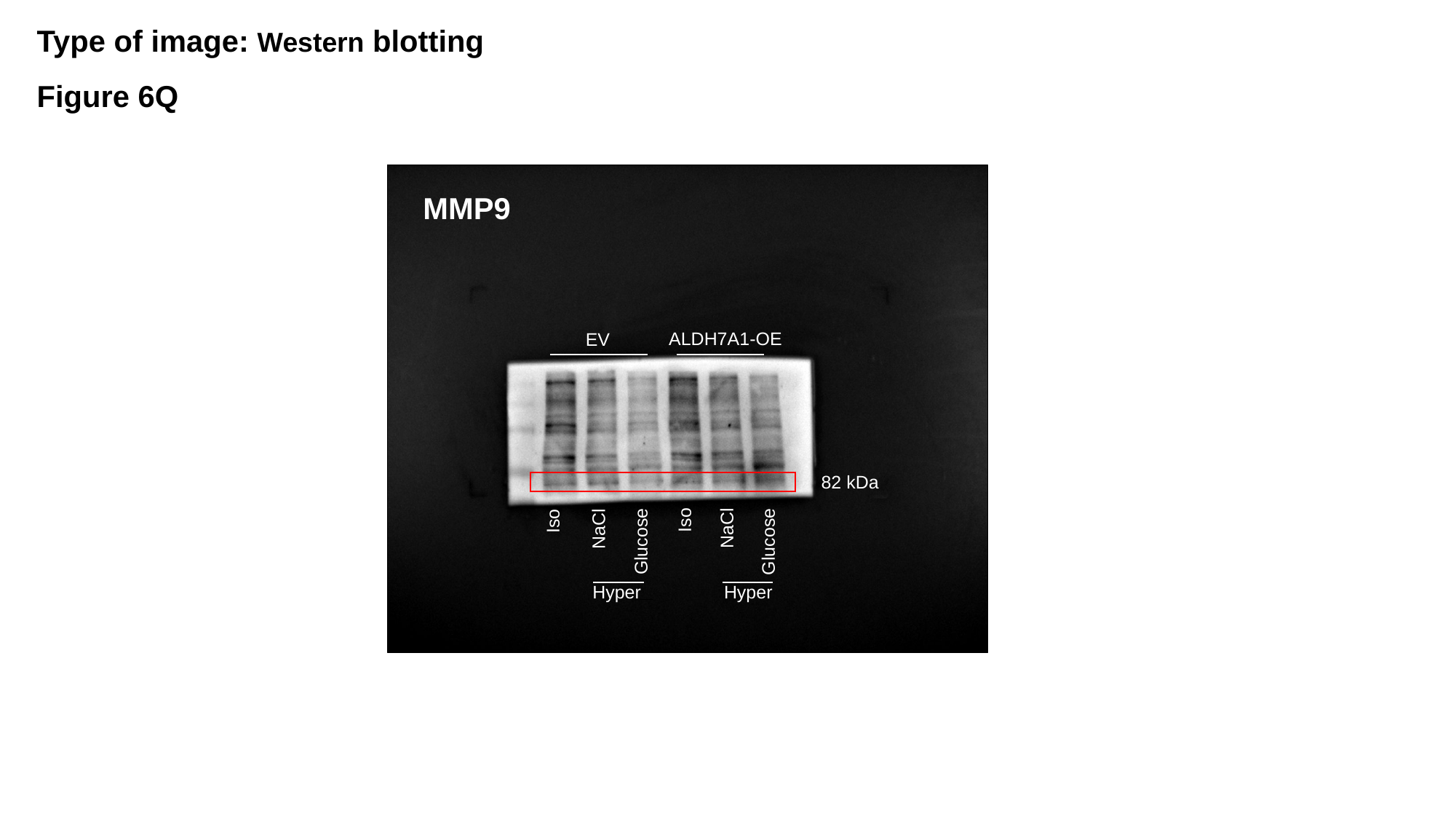

Type of image: Western blotting
Figure 6Q
MMP9
ALDH7A1-OE
EV
82 kDa
Iso
Iso
NaCl
NaCl
Glucose
Glucose
Hyper
Hyper

## Slide 25
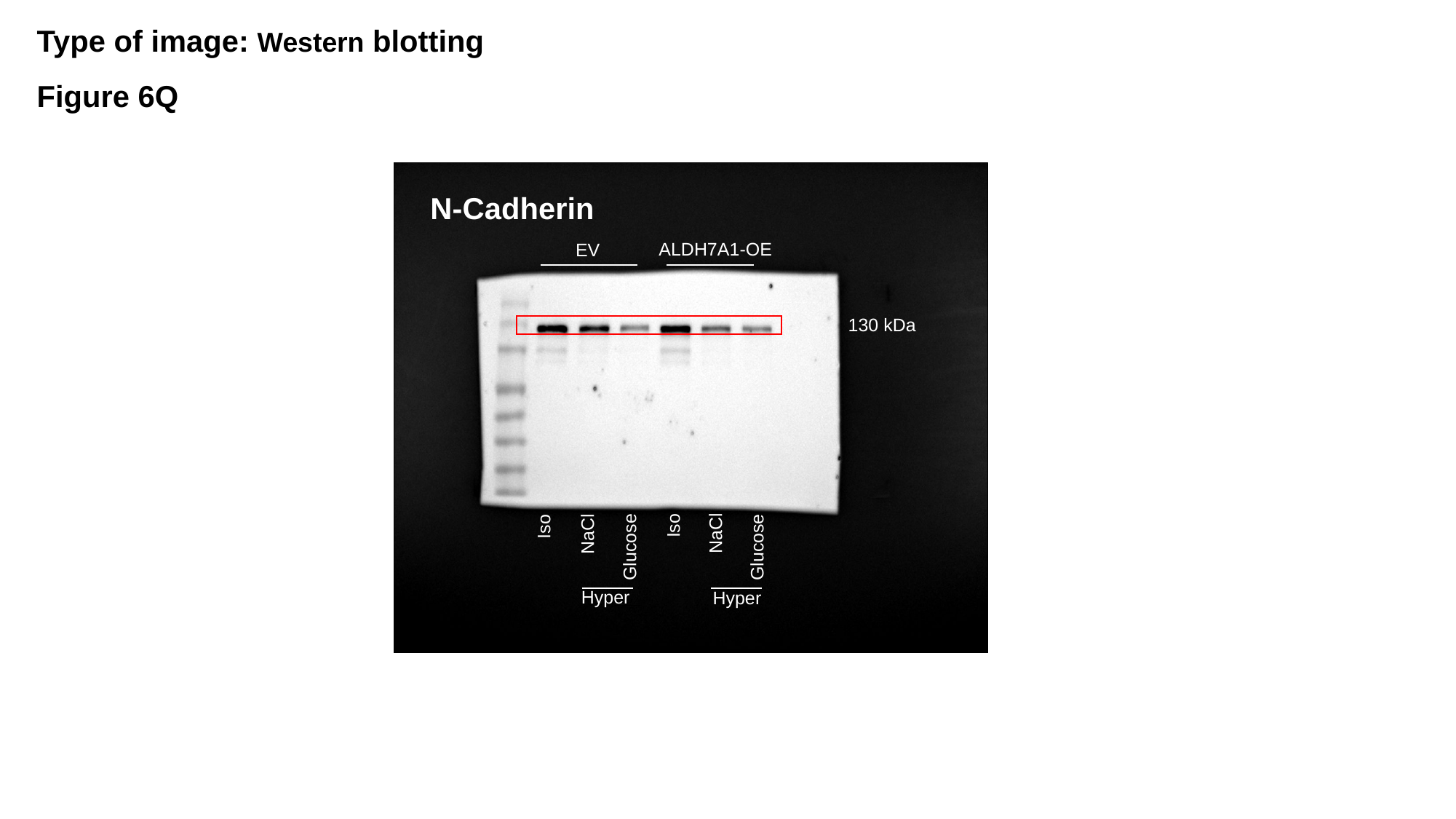

Type of image: Western blotting
Figure 6Q
N-Cadherin
ALDH7A1-OE
EV
130 kDa
Iso
Iso
NaCl
NaCl
Glucose
Glucose
Hyper
Hyper

## Slide 26
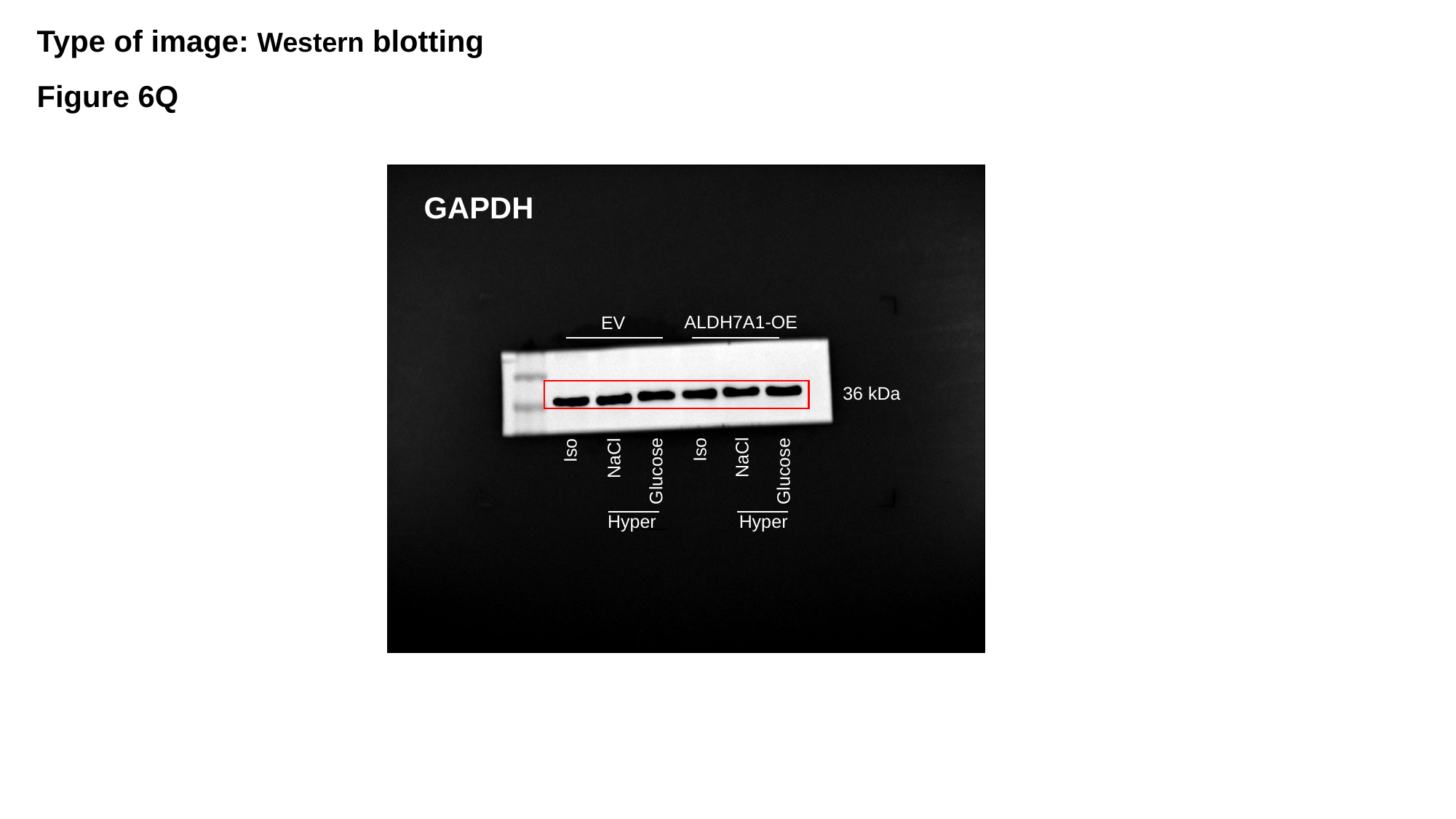

Type of image: Western blotting
Figure 6Q
GAPDH
ALDH7A1-OE
EV
36 kDa
Iso
Iso
NaCl
NaCl
Glucose
Glucose
Hyper
Hyper

## Slide 27
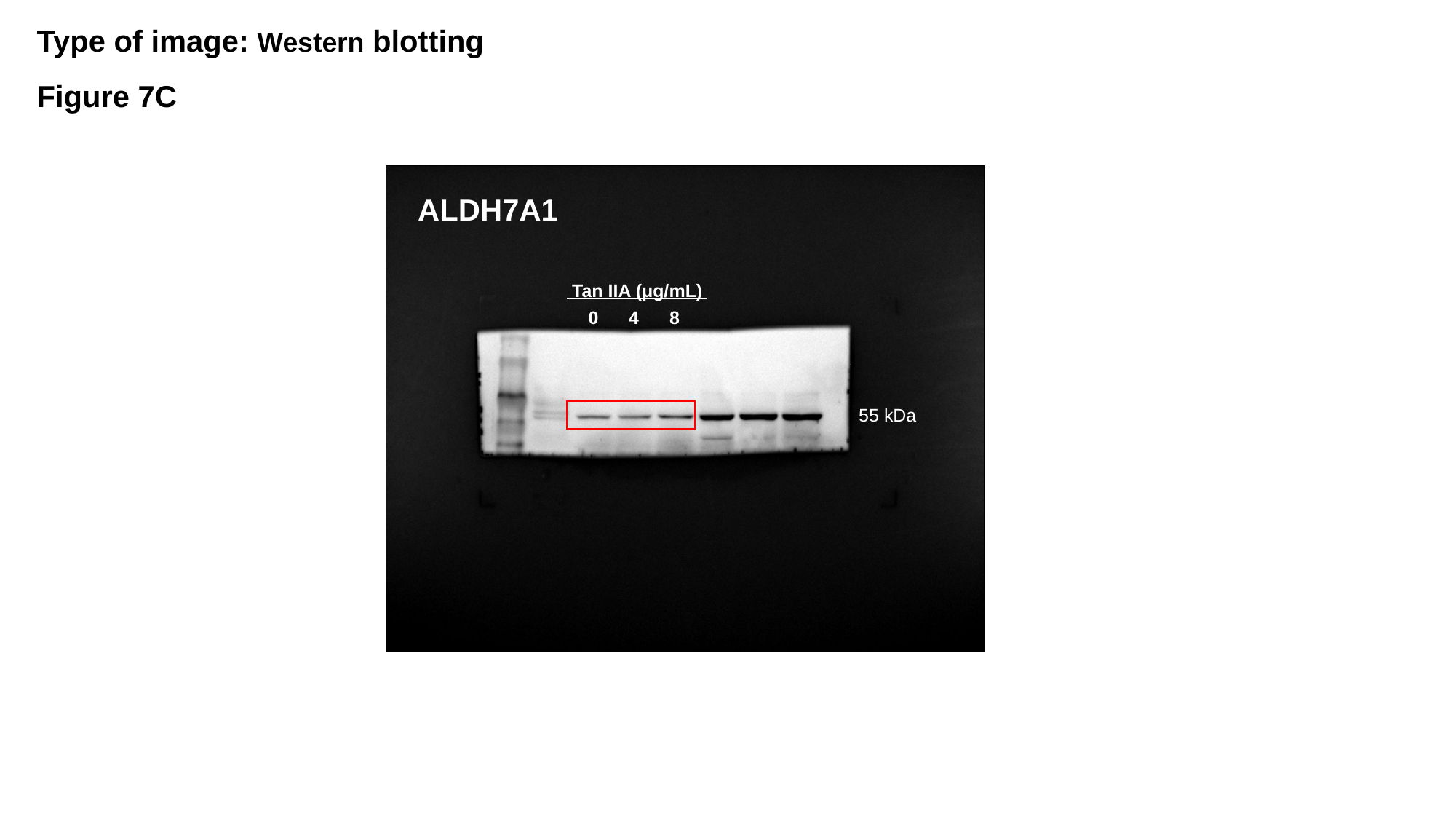

Type of image: Western blotting
Figure 7C
ALDH7A1
 Tan IIA (μg/mL)
 0 4 8
55 kDa

## Slide 28
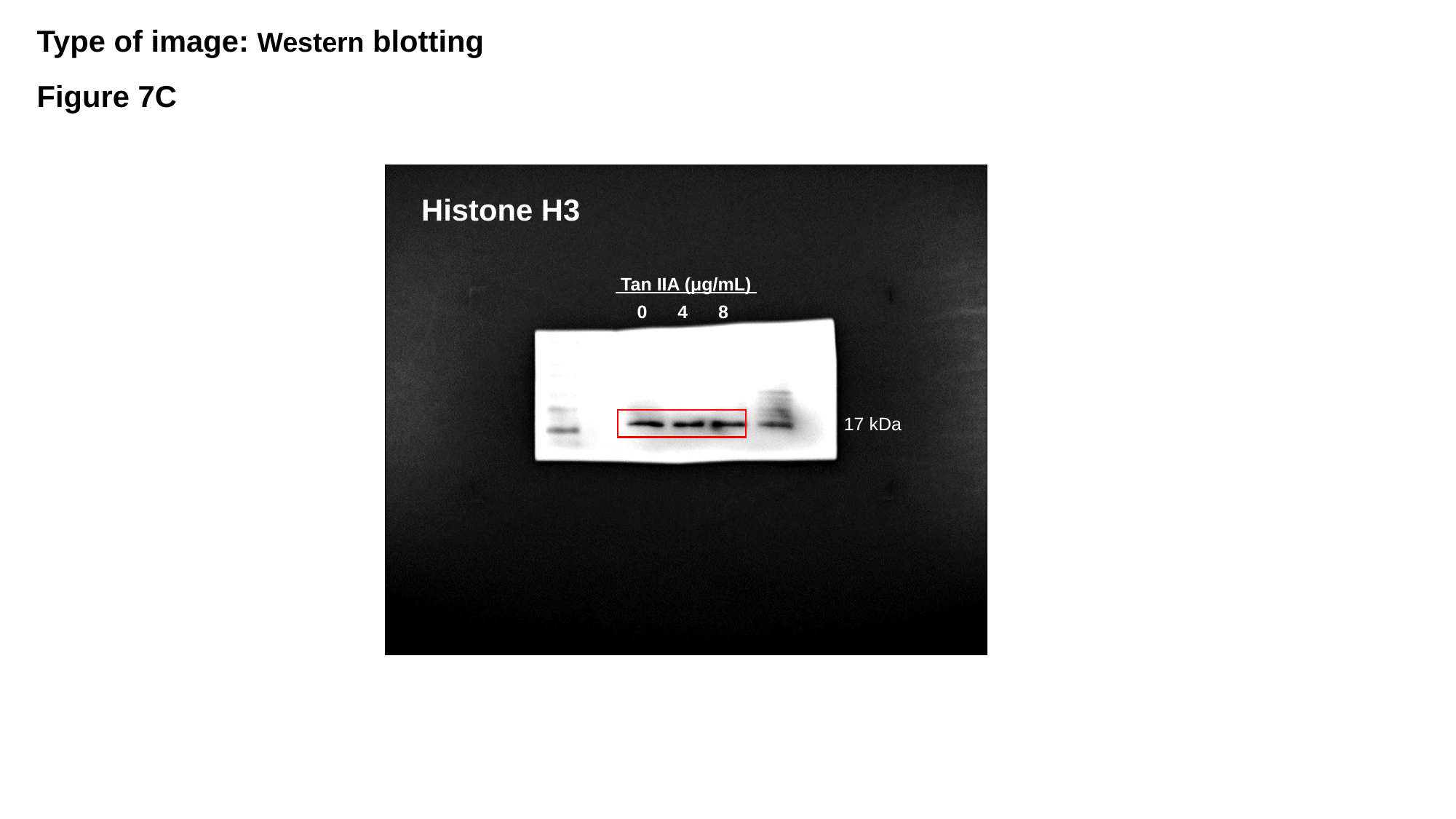

Type of image: Western blotting
Figure 7C
Histone H3
 Tan IIA (μg/mL)
 0 4 8
17 kDa

## Slide 29
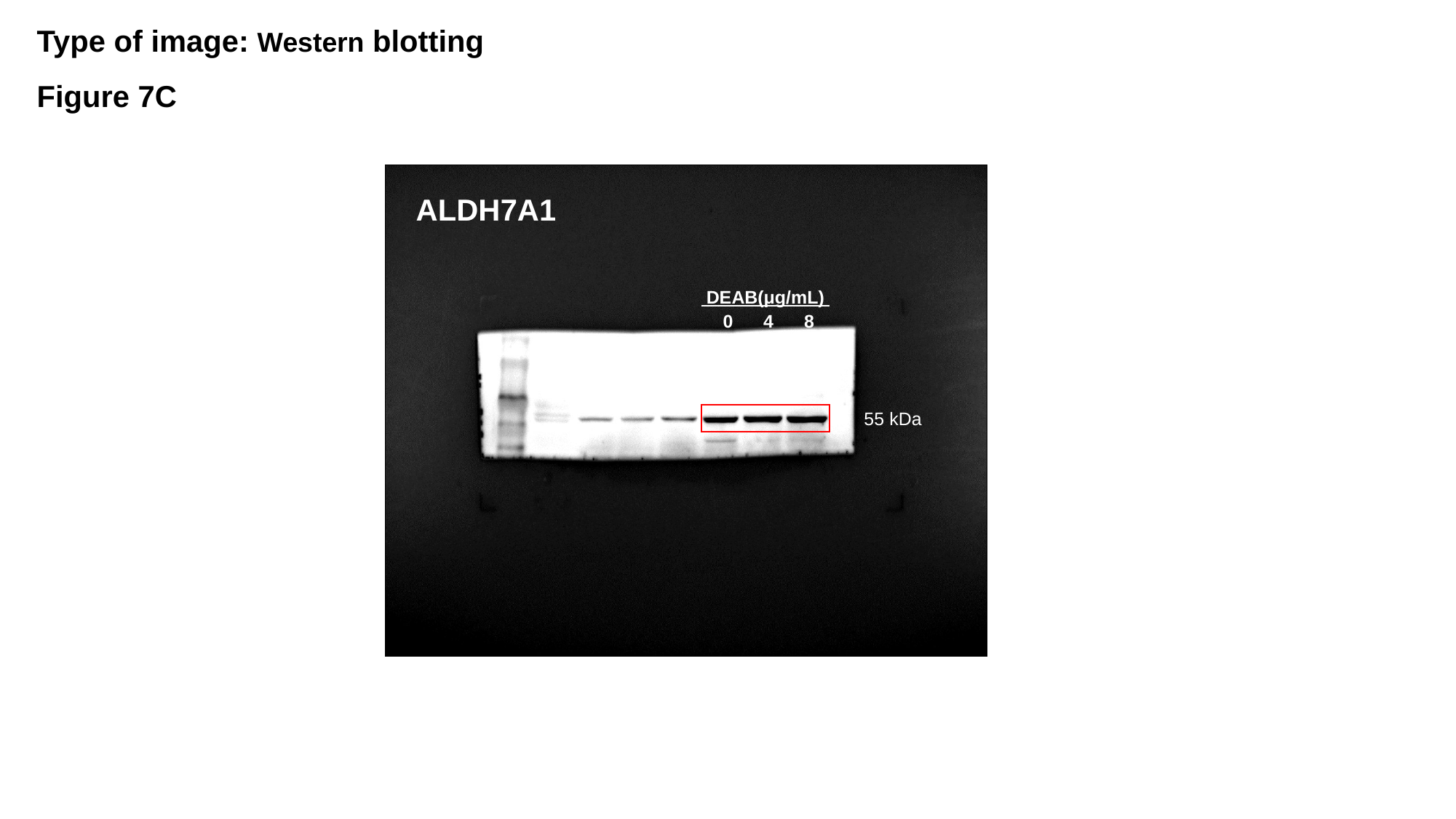

Type of image: Western blotting
Figure 7C
ALDH7A1
 DEAB(μg/mL)
 0 4 8
55 kDa

## Slide 30
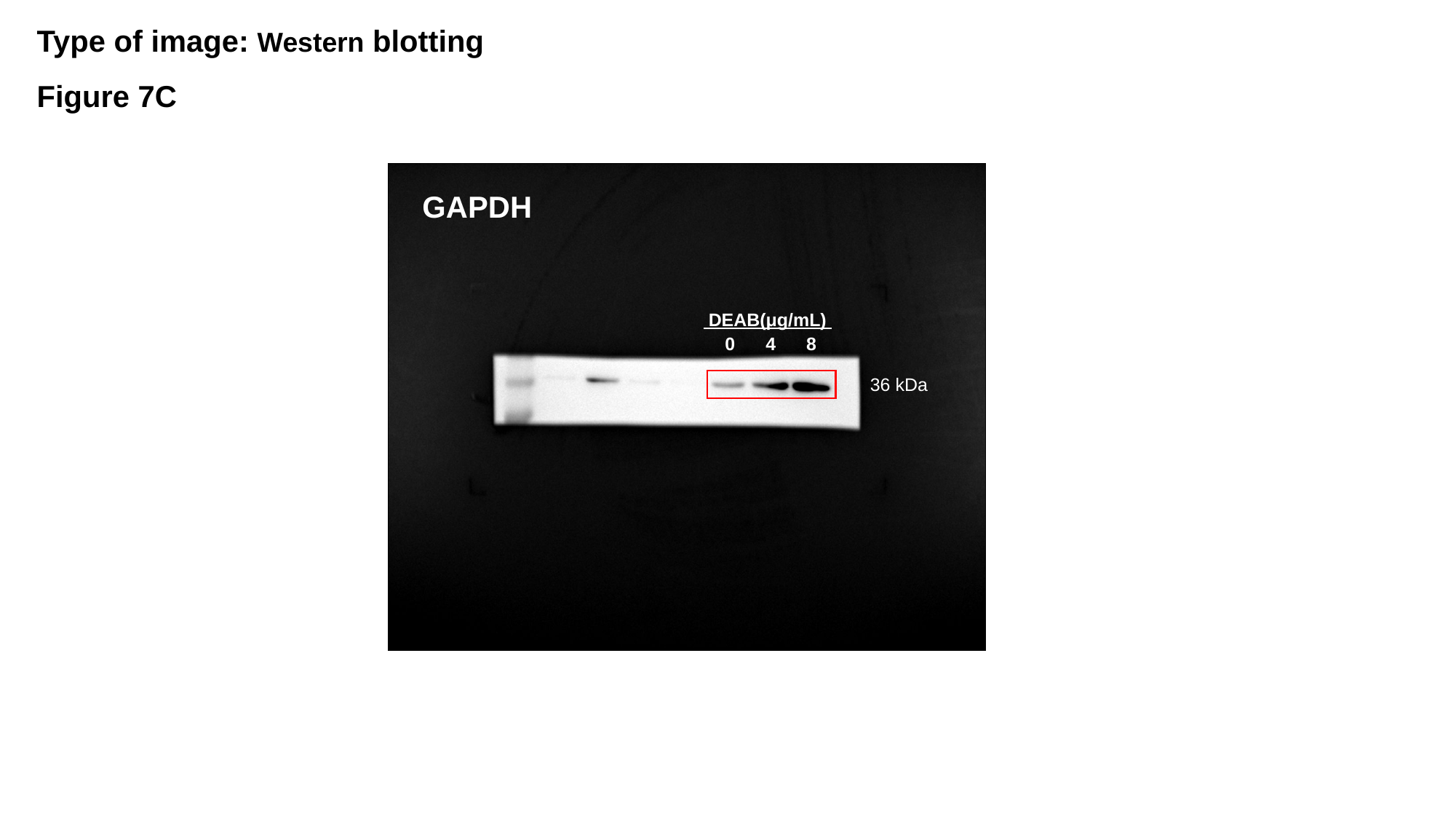

Type of image: Western blotting
Figure 7C
GAPDH
 DEAB(μg/mL)
 0 4 8
36 kDa

## Slide 31
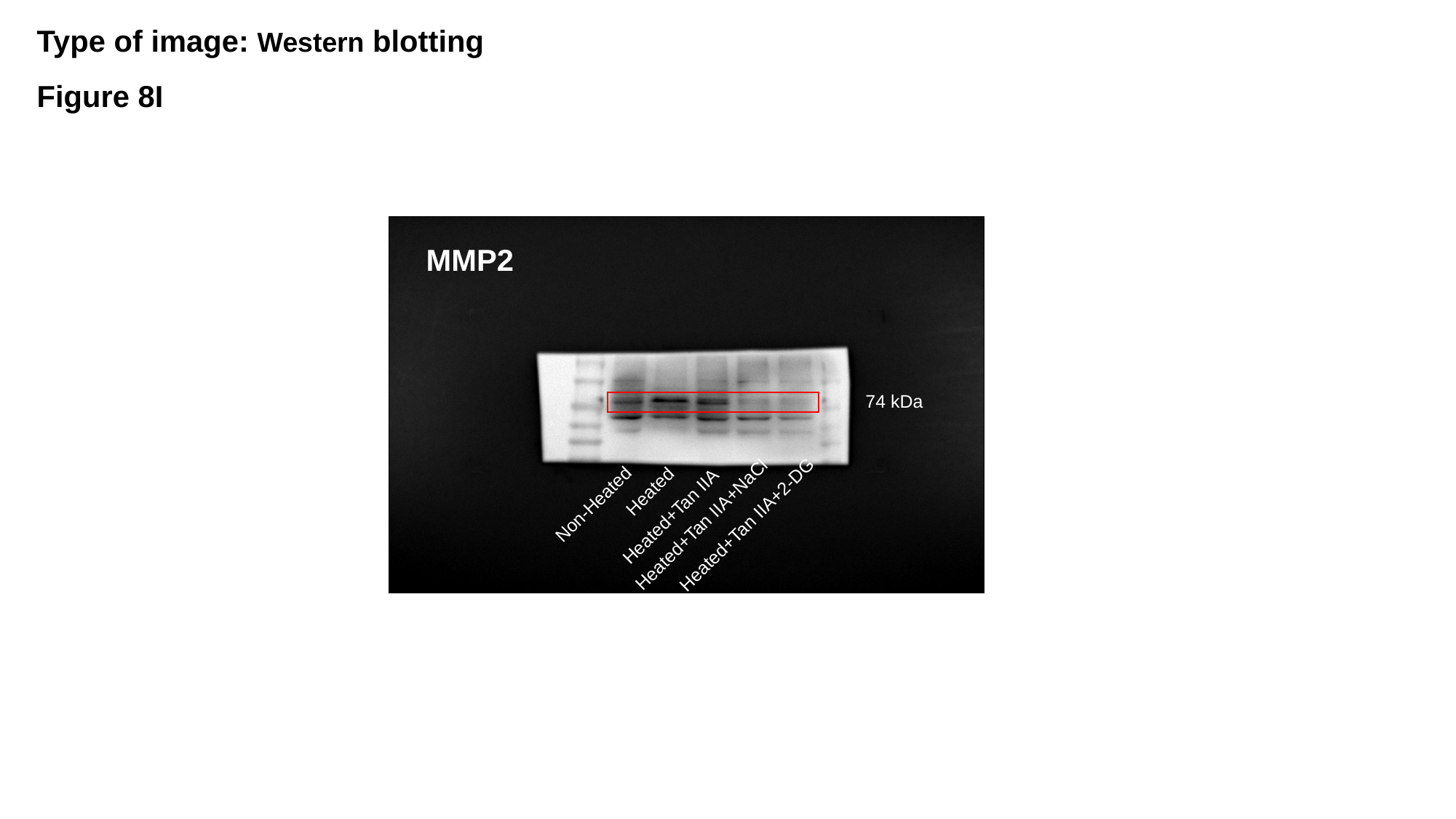

Type of image: Western blotting
Figure 8I
MMP2
74 kDa
Heated
Non-Heated
Heated+Tan IIA
Heated+Tan IIA+NaCl
Heated+Tan IIA+2-DG

## Slide 32
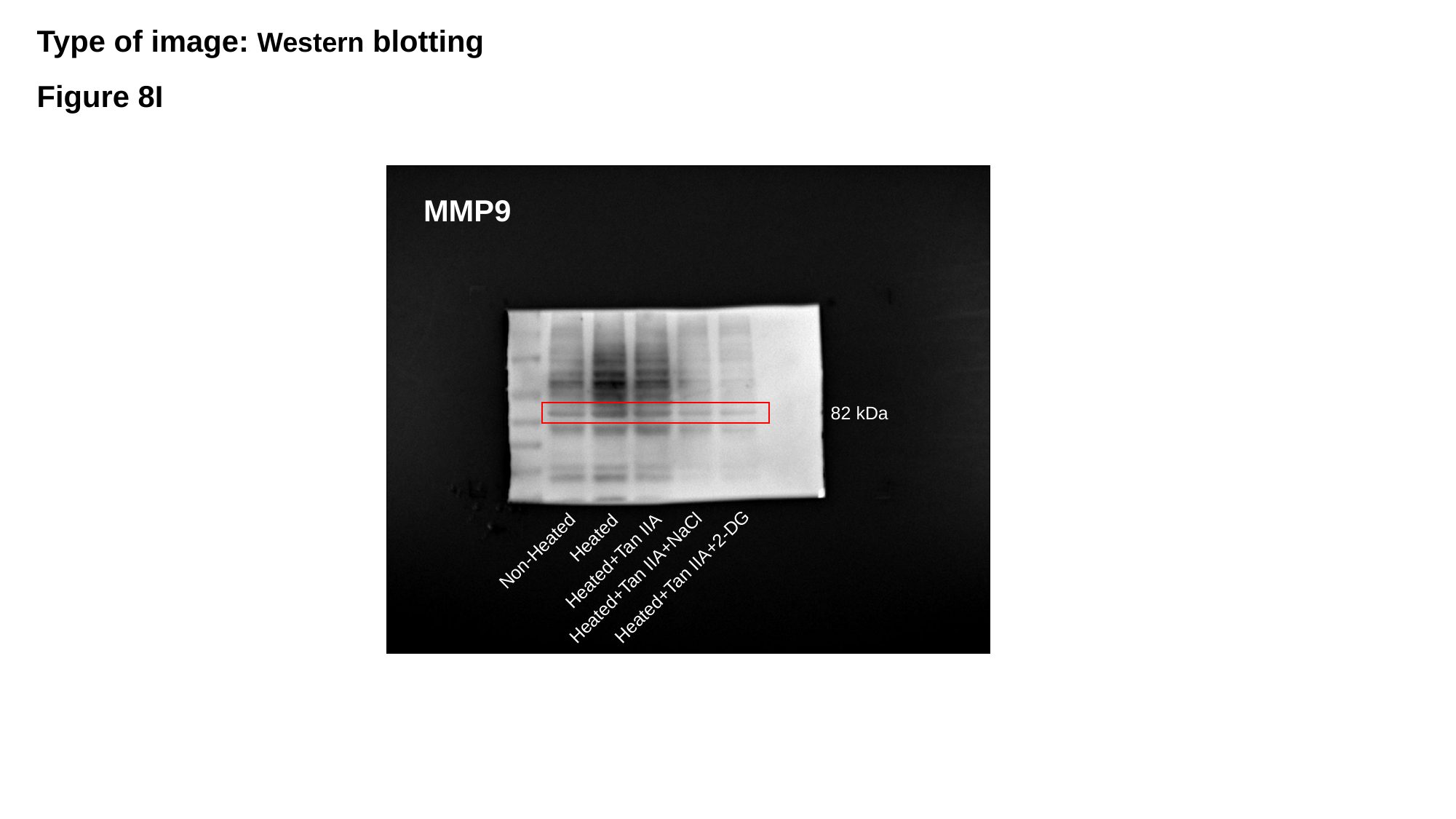

Type of image: Western blotting
Figure 8I
MMP9
82 kDa
Heated
Non-Heated
Heated+Tan IIA
Heated+Tan IIA+NaCl
Heated+Tan IIA+2-DG

## Slide 33
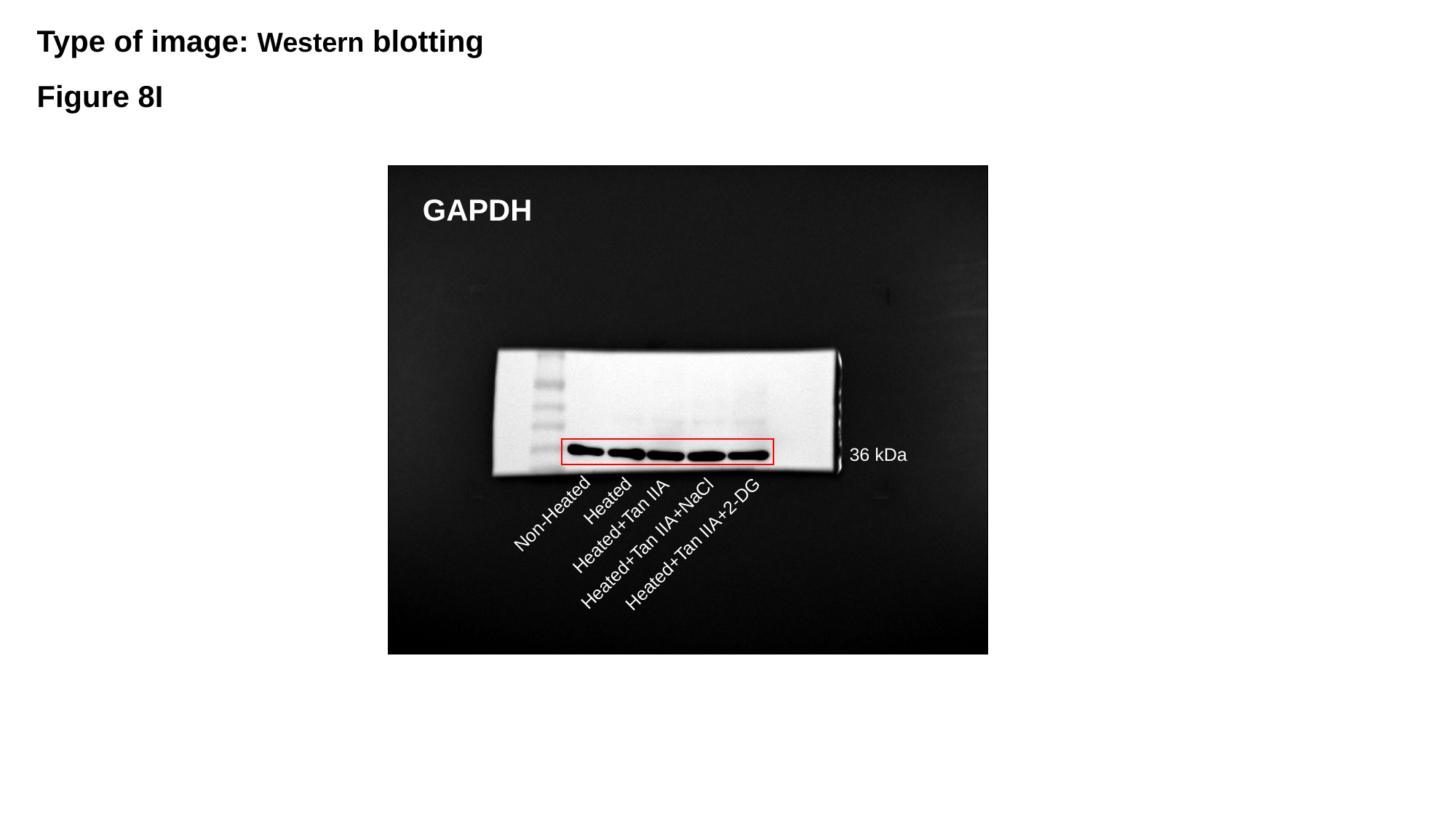

Type of image: Western blotting
Figure 8I
GAPDH
36 kDa
Heated
Non-Heated
Heated+Tan IIA
Heated+Tan IIA+NaCl
Heated+Tan IIA+2-DG
